# Fatigue-Resistant Dithienylarene Photoswitches with Acid-Regulated Thermal Ring Opening

**DOI:** 10.1021/acsomega.6c01422

**Published:** 2026-05-18

**Authors:** Attila Kunfi, Barnabás Zsignár-Nagy, D. Sravanakumar Perumalla, Bo Durbeej, Gábor London

**Affiliations:** † Institute of Organic Chemistry, 280964 HUN-REN Research Centre for Natural Sciences, Magyar tudósok krt. 2, Budapest 1117, Hungary; ‡ Hevesy György PhD School of Chemistry, Eötvös Loránd University, Pázmány Péter sétány 1/A, Budapest 1117, Hungary; § Division of Theoretical Chemistry, IFM Linköping University, Linköping SE-58183, Sweden; ∥ Department of Applied Sciences and Humanities, 471415 Sasi Institute of Technology & Engineering, Tadepalligudem 534 101, India

## Abstract

Dithienylarene photoswitches are an emerging class of diarylethenes, in which photochemical ring closure is accompanied by pronounced changes in aromaticity. This distinctive feature enables new opportunities for applications, including molecular solar thermal energy (MOST) storage, a promising approach within renewable energy technologies. Molecular switches used as MOST systems are designed to absorb solar energy, store it in chemical bonds, and release it as heat. However, fast and efficient on-demand release of energy poses a significant challenge. Herein, we present the synthesis of a pyridine-appended, biphenylene-bridged dithienylarene switch, **BPPyr**, and demonstrate that its protonation state exerts a major influence on the kinetics of its thermal ring opening. Specifically, protonation of the pyridine units (**[BPPyr-H_2_]^2+^**) drastically reduces the thermal half-life of the ring-closed isomer from 2.9 h to 15 s in EtOH at 25 °C but, crucially, without significantly deteriorating the fatigue resistance upon repeated isomerization cycles. The parent **BPPyr** form with its original properties could be recovered via deprotonation; furthermore, *N*-methylation of **BPPyr** produced a water-soluble, ionic compound (**[BPPyr-Me_2_]^2+^**) switchable by visible light. Leveraging its unique properties also in a confined environment possibly akin to that of a future device, **[BPPyr-Me_2_]^2+^** was incorporated in a gelatin matrix to form a sunlight-responsive hydrogel. Complementing the experimental results, quantum chemical calculations were performed to explain the mechanism by which protonation/methylation of **BPPyr** lowers the free-energy barrier for thermal ring opening. Altogether, this work advances the understanding of structure–property relationships for diarylethenes and provides a novel design for on-demand energy release by MOST systems.

## Graphical abstract

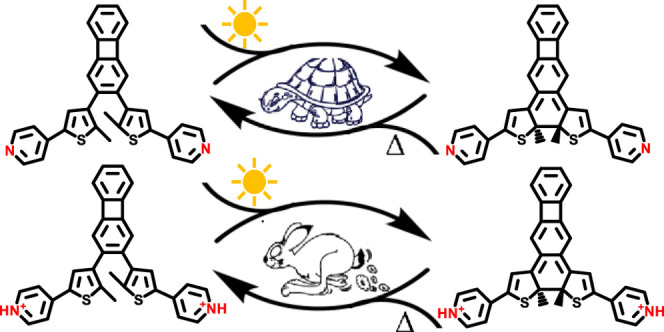

## 1 Introduction

Dithienylethenes^1–3^ are among the most widely studied diarylethene-type photochromic switches that are able to undergo reversible, light-induced isomerization between their open and closed forms (Figure 1a). The origin of their popularity is the thermal stability of both isomers and the generally high fatigue resistance upon repeated isomerization cycles. Due to these advantages, dithienylethenes are commonly used for the reversible photochemical control of catalytic function,^4^ material properties,^5,6^ and biological phenomena.^6,7^ Complementary to dithienylethenes, dithienyl­(hetero)­arenes (Figure 1b) are emerging structures^8–15^ in which pronounced changes in aromaticity are induced by the photochemical ring closing (cyclization), while their ring opening can proceed in a thermal fashion (T-type photochromism).^10,11,16–19^ These features of dithienyl­(hetero)­arenes offer new possibilities for applications. For example, as the aromaticity changes have major energetic consequences, switches of this type have been proposed to be suitable chromophores for the design of molecular solar thermal energy (MOST) storage systems, with thermally triggered release of the stored solar energy.^15,16,18,20–22^ Furthermore, the fast and thermally reversible changes of conjugation in diarylbenzenes have been shown to produce efficient photomultiplication in organic photodiodes.^23,24^

**Figure 1:**
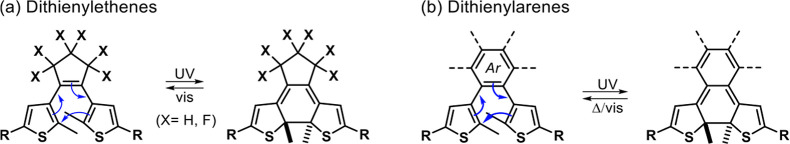
Structures and reversible isomerization of (a) dithienylethene- and (b) dithienylarene-type photoswitches.

Among the many challenges facing practical applications of molecular photoisomers,^23–31^ dithienylarenes excel in efficient photocyclization, thanks to the onset of antiaromaticity in the photoactive excited state.^14,16,21^ Due to the loss of aromaticity in their closed isomers, they furthermore offer high energy-storage densities^18,19,21^ for potential MOST applications and facilitate efficient electron transfer in organic photodetectors.^23,24^ Beyond efficient photocyclization, for many uses, it is also desirable to be able to tune the ring opening process. This holds true especially for MOST applications,^20,32,33^ where ring opening is responsible for releasing the stored solar energy. Notably, progress has been made toward catalyzing the energy release from both norbornadiene- and azaborine-based MOST systems with transition-metal complexes like cobalt phthalocyanine^32^ and the Wilkinson catalyst,^34^ respectively. While such catalysis certainly provides an efficient route for the energy release from these systems, for both dithienylethenes^20,35^ and dithienylarenes,^22^ a cheaper and simpler strategy has emerged where this step instead is accelerated through the addition of acid. Recently, the groups of Nakatani and Métivier reported^22^ a dithienylheteroarene photoswitch having pyridine substituents on the thienyl units. They showed that the ring opening of its closed isomer can be accelerated by the addition of acid, which can be applied for on-demand energy release. However, while, from the perspective of using dithienylarenes as MOST systems, this possibility is very appealing and forgoes the need to use an expensive transition-metal catalyst, the corresponding study^22^ focused much less on the issue of fatigue resistance, which is a key issue for the operation of any molecular switch.^36^ In fact, given that the acid-catalyzed energy release by the dithienylarene in question is believed to operate through a destabilization of the ring-closed isomer upon protonation,^22^ it could well be that a protonated dithienylarene exhibits much worse fatigue resistance than a neutral one, as both photochemical and thermal side reactions influence the fatigue resistance.^37–41^

Against this background, in the current work, besides studying a different dithienylarene to confirm and extend the previous findings^22^ on acid-catalyzed energy release from the ring-closed form of these switches, we investigate and compare the fatigue resistance of both protonated and neutral forms of dithienylarenes. Interestingly, we find that the fatigue resistance of the protonated form compares rather well to that of the neutral form, which is very good news for the future possibility to exploit acid catalysis in the operation of MOST systems based on dithienylarenes. Moreover, looking to demonstrate the potential of our dithienylarene design for practical applications, which likely will require reversible isomerization to be feasible in a confined environment,^42–44^ we show that a methylated version of our design does have this ability also when embedded in a gelatin hydrogel. Finally, by performing quantum chemical calculations, we shed new light on the mechanism for the acid catalysis.

## 2 Results and Discussion

In the following, we first describe the synthesis of a pyridine-appended, biphenylene-based dithienylarene switch, **BPPyr**, and then investigate its photochemical and thermal properties by UV–vis and NMR spectroscopy. Next, we describe the effect of the added acid on its switching performance. Along with the protonated species **[BPPyr-H_2_]^2+^**, the properties of the bis­(*N*-methylated) derivative**[BPPyr-Me_2_]^2+^** are also studied. Finally, we rationalized our findings computationally.

### 2.1 Synthesis

**BPPyr** and **[BPPyr-Me_2_]^2+^** switches were prepared through a convergent synthesis strategy (Figure 2). First, a boronic acid moiety was introduced onto a pyridine-substituted 2-methylthiophene ring (**4**).^45^ In parallel, the biphenylene unit **9** was accessed via a cobalt-catalyzed [2 + 2 + 2] cycloaddition of bisacetylene **7** and bis­(trimethylsilyl)­acetylene (BTMSA), using BTMSA as the solvent.^17^ Substitution of the TMS groups of **8** by iodine using *N*-iodosuccinimide provided **9**. BPPyr was then synthesized via a Suzuki coupling reaction of diiodobiphenylene **9** and boronic acid **4**. *N*-Methylation of the pyridine rings of **BPPyr** with MeI gave **[BPPyr-Me_2_]^2+^**.

**Figure 2:**
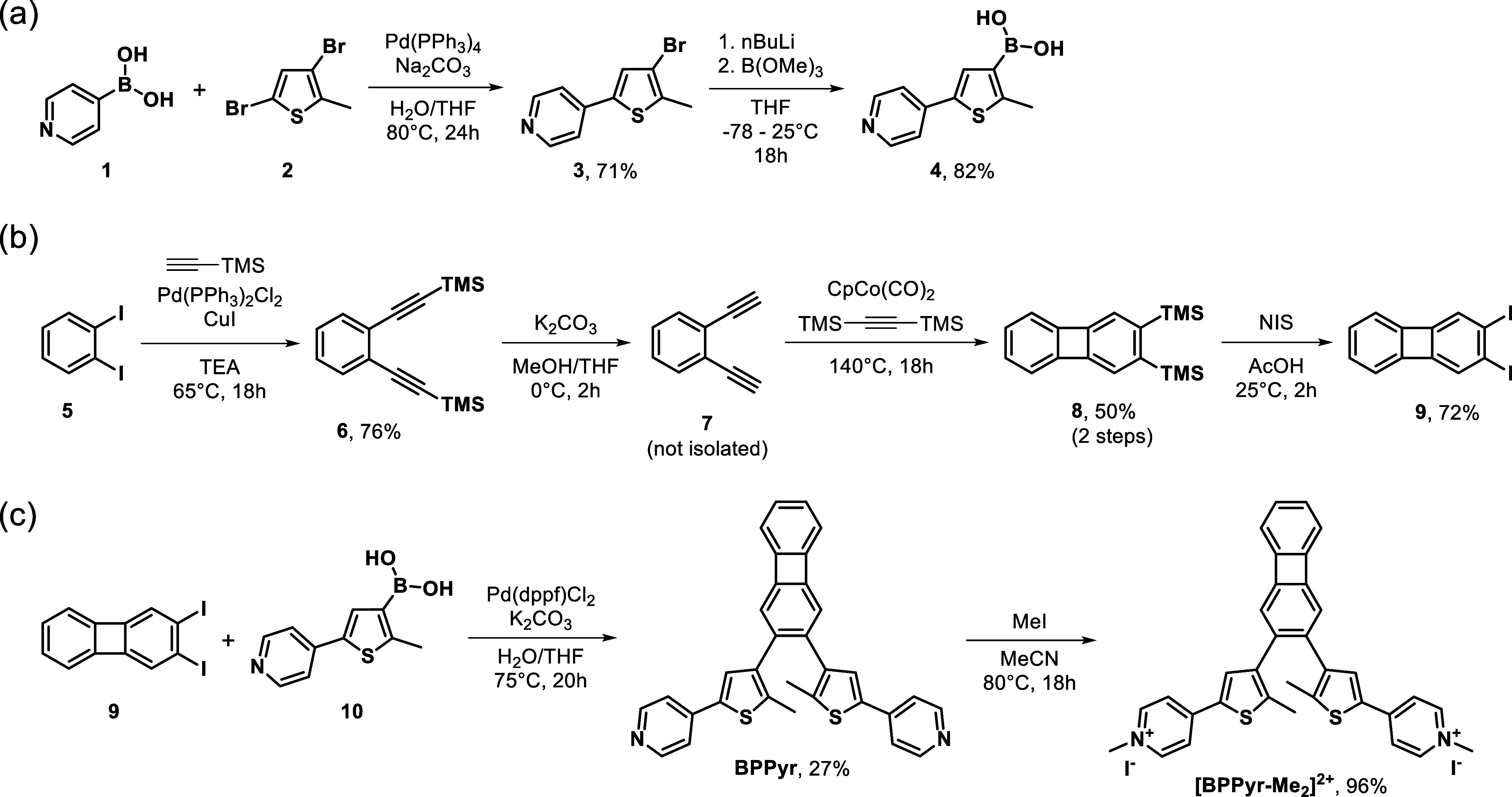
Synthesis of the (a) thienyl subunit, (b) biphenylene subunit, and (c) final **BPPyr** and **[BPPyr-Me_2_]^2+^** photoswitch structures.

### 2.2 Spectroscopic Properties of BPPyr

The optical properties of the synthesized neutral **BPPyr** switch were first characterized by UV–vis spectroscopy. The ring open isomer, **BPPyr-o**, showed absorptions below 400 nm in MeCN solution. Upon irradiation with 365 nm light (10 W LED; for the corresponding photon flux values, see Section S1, Supporting Information), **BPPyr-o** underwent photocyclization to the ring-closed isomer, **BPPyr-c**, within 30 s (Figure 3a). The light-induced structural change was indicated by the appearance of two new absorption bands around 410 and 670 nm. The photochemical ring closing was found to be reversible, as irradiation of the solutions of **BPPyr-c** with 620 or 440 nm light regenerated the original spectrum attributed to **BPPyr-o** (see also Figure S1, Supporting Information). The irradiation process was tested in a range of solvents (Figure 3b), but no pronounced solvent effect was observed. Notably, by replacing the 10 W light source with a 30 W device, the closed form could be generated within 10 s. Quantum yields (Φ) of the light-induced ring closing (Φ_RC_) and ring opening (Φ_RO_) reactions for **BPPyr** were determined^46,47^ to be Φ_RC_ = 0.12 and Φ_RO_ = 0.0043, respectively (see also Section S2.2, Supporting Information).

**Figure 3:**
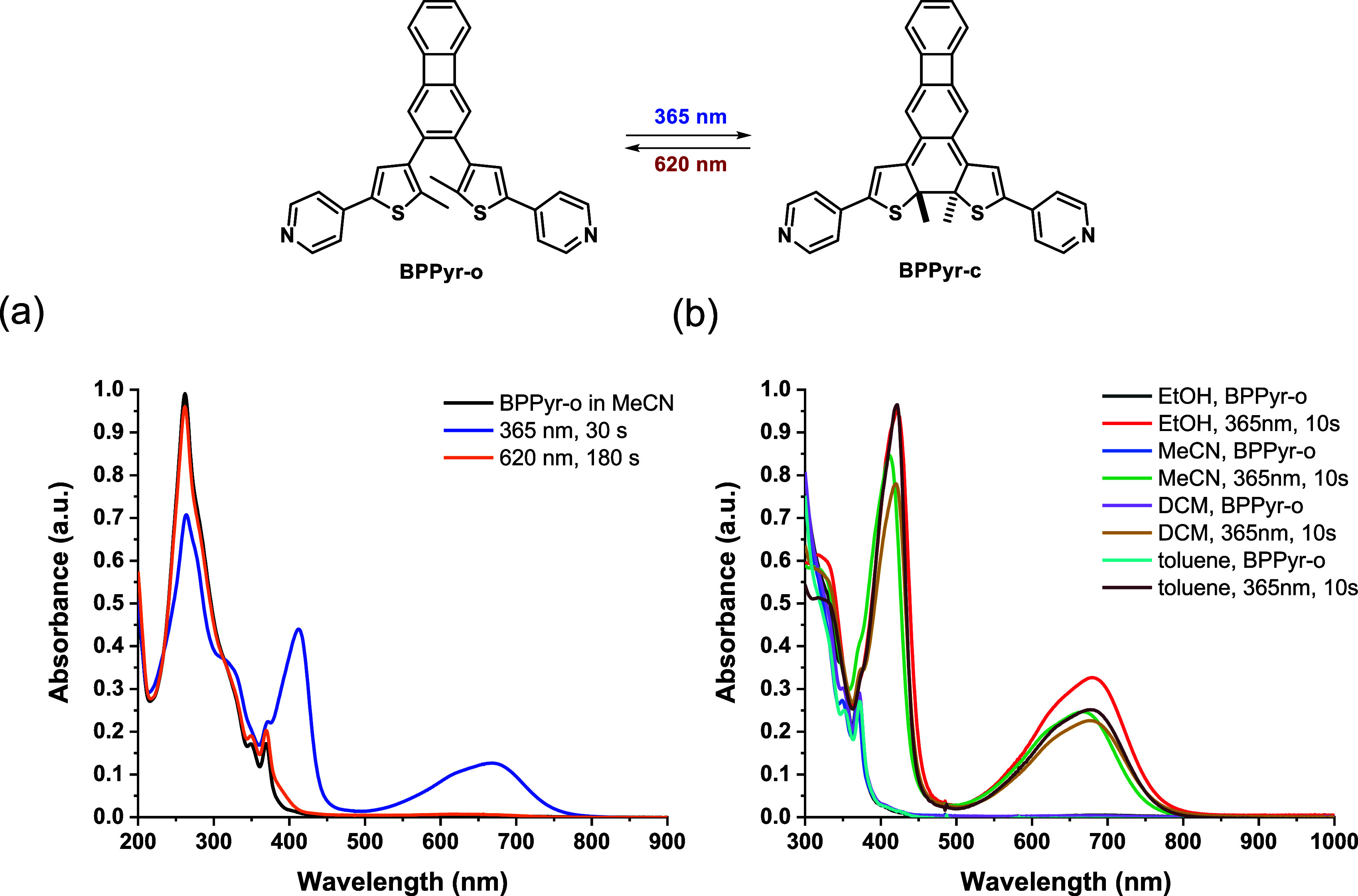
Changes in the UV–vis absorption spectrum of **BPPyr** (*c* = 5 × 10^–5^ M) (a) in MeCN after UV (365 nm) and visible light (620 nm) irradiation with a 10 W LED and (b) in different solvents upon irradiation with a 30 W, 365 nm LED.

The thermal stability of **BPPyr-c** was also investigated. In contrast to the photochemical transformations, in this case pronounced solvent effects were observed. Specifically, the half-life times (*t*_1/2_) of the thermal ring opening process of **BPPyr-c** were found to be quite different in different solvents (Table 1). More precisely, in strongly polar solvents, such as EtOH and MeCN, the *t*_1/2_ values were around 3 h at 25 °C, while in the less polar, aprotic solvents toluene and DCM, the *t*_1/2_ values decreased to about 1.2 h and 6 min, respectively. Notably, for a related system, similar thermal stability of the closed isomer was found in CH_3_CN.^22^ The deeper understanding of the solvent effect on thermal stability warrants further research.

**Table 1:** Spectroscopic Data of BPPyr Isomers, Half-Life Times (*t*_1/2_^25^°^C^), and Reaction Rate Constants (*k*) at 25 °C for the Thermal Ring Opening of BPPyr-c in Different Solvents^a^

entry	solvent	λ^o^_max_ (nm)	ε^o^_max_ (M^–1^ cm^–1^)	λ^*c*^_max_ (nm)	*k* (s^–1^)	*t*_1/2_^25^°^C^ (s)
1	EtOH	370	17,600	680	6.76 × 10^–5^	10,441
2	MeCN	369	16,494	667	6.42 × 10^–5^	10,805
3	DCM	371	17,441	678	1.90 × 10^–3^	364
4	toluene	372	16,273	677	1.65 × 10^–4^	4208

a λ^o^_max_ and λ^*c*^_max_ refer to the wavelengths of maximum absorbance of the open and closed isomers, respectively. ε^o^_max_ refers to the molar absorbance at λ^o^_max_.

^1^H NMR spectroscopy was used to confirm the reversible structural changes between **BPPyr-o** and **BPPyr-c**. **BPPyr-o** was irradiated (365 nm) in various solvents until no further changes were observed in the corresponding ^1^H NMR spectra (Figure 4 and Figures S2–S4, Supporting Information). Characteristic peaks of the pyridine rings that appeared at around 8.45 and 8.55 ppm for **BPPyr-o** and **BPPyr-c**, respectively, were used to determine the ratio of the two forms. The ratios at the photostationary state (PSS) in different solvents are listed in Table 2. (Note that about 2–3 min passed between the end of the irradiation and the recording of the spectra.) The highest ratio of **BPPyr-c** was observed in C_6_D_6_ (70%) and the lowest in CD_2_Cl_2_ (26%). The latter result is in accordance with the relatively fast thermal ring opening of **BPPyr-c** observed in DCM. Interestingly, for the photoinduced ring closing reaction of diarylcycloalkenes, previous studies have documented solvent effects in the form of differing UV–vis spectral intensities at the PSS.^48,49^ For the present system, however, the UV–vis spectra do not indicate any such effects (Figure 3).

**Figure 4:**
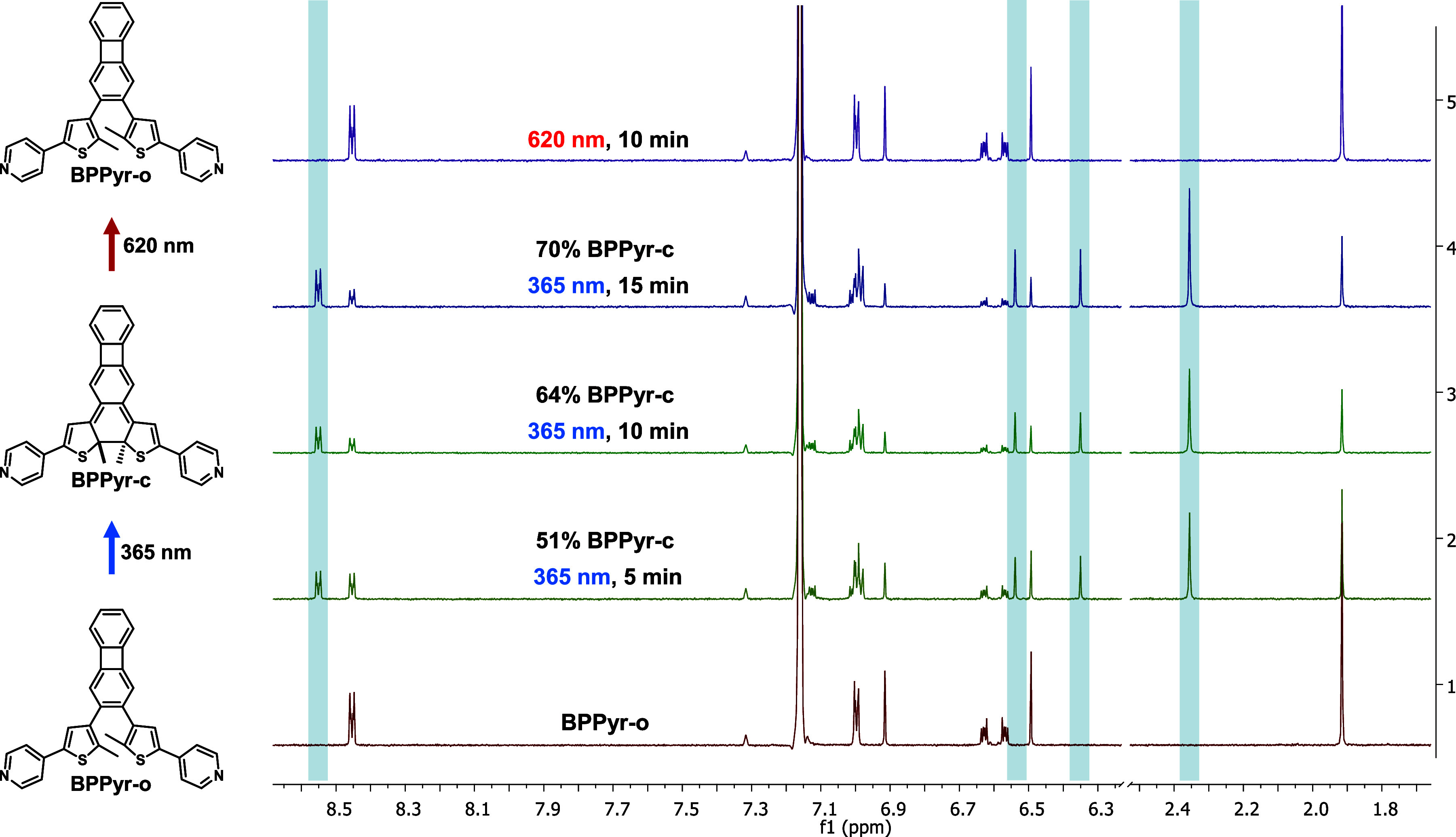
^1^H NMR (500 MHz) spectral changes of **BPPyr-o** upon 365 and 620 nm irradiation in C_6_D_6_ at 25 °C with 10 W LEDs.

**Table 2:** Distribution of BPPyr Isomers at Different Irradiation (365 nm, 10 W) Times in Various Deuterated Solvents (*c* = 5 mM) Derived from Their Experimental ^1^H NMR Spectra

entry	solvent	irradiation time (min)	conversion rate at PSS (%)
1	C_6_D_6_	5	51
2	C_6_D_6_	10	64
3	C_6_D_6_	15	70
4	MeOD	5	39
5	MeOD	10	53
6	MeOD	15	63
7	CD_3_CN	5	34
8	CD_3_CN	10	47
9	CD_3_CN	15	52
10	CD_2_Cl_2_	5	12
11	CD_2_Cl_2_	10	26
12	CD_2_Cl_2_	15	26

Illustrative ^1^H NMR spectra (500 MHz) showing the structural changes upon irradiation with 365 nm light in C_6_D_6_ at room temperature are displayed in Figure 4. Importantly, the spectra reveal a clean photochemical transformation of **BPPyr-o** into **BPPyr-c** **,** and similarly a clean reverse reaction upon irradiation with 620 nm light that regenerates the original ^1^H NMR spectrum of **BPPyr-o**. We tested both 10 and 30 W light sources during the irradiation process. The higher power LED enabled faster formation of **BPPyr-c** (about 70% in 3 min); however, the appearance of a side product was observed (about 5% in 3 min) over time in this case. On the basis of ^1^H NMR spectra, we assume this side product to be a bis­(dihydrothiopyran)-type species (Figure S5, Supporting Information).^37,41^

### 2.3 Effect of Protonation

To test the effect of protonation on the photochemical and thermal properties of **BPPyr**, we first added 10 equiv of methanesulfonic acid (MsOH) to a solution of **BPPyr-o** in EtOH. Organic solutions of MsOH can be conveniently prepared compared to volatile aqueous acids (e.g., HCl). The effect of the acid was apparent from the changes in the associated UV–vis spectrum. Compared to neutral **BPPyr-o**, the spectrum of the protonated derivative **[BPPyr-H_2_]^2+^-o** exhibited two distinct and somewhat red-shifted absorption bands below 450 nm (Figure 5a). Irradiation of **[BPPyr-H_2_]^2+^-o** with 365 nm light induced spectral changes similar to those observed for the neutral species, indicating the formation of the ring-closed protonated isomer **[BPPyr-H_2_]^2+^-c**. Specifically, new bands appeared in the visible region, however, at considerably longer wavelengths (472 and 762 nm in EtOH) compared to what was observed for neutral **BPPyr-c** (421 and 680 nm). Importantly, this change was reversible, as irradiation with 660 nm light regenerated the original UV–vis spectrum of **[BPPyr-H_2_]^2+^-o**. Qualitatively similar observations were made in all solvents considered (EtOH, DCM, MeCN, and toluene) (Figure S6, Supporting Information). Due to the red-shifted absorption of **[BPPyr-H_2_]^2+^-o**, irradiation with a 405 nm visible light also produced **[BPPyr-H_2_]^2+^-c** (Figure S6, Supporting Information).

**Figure 5:**
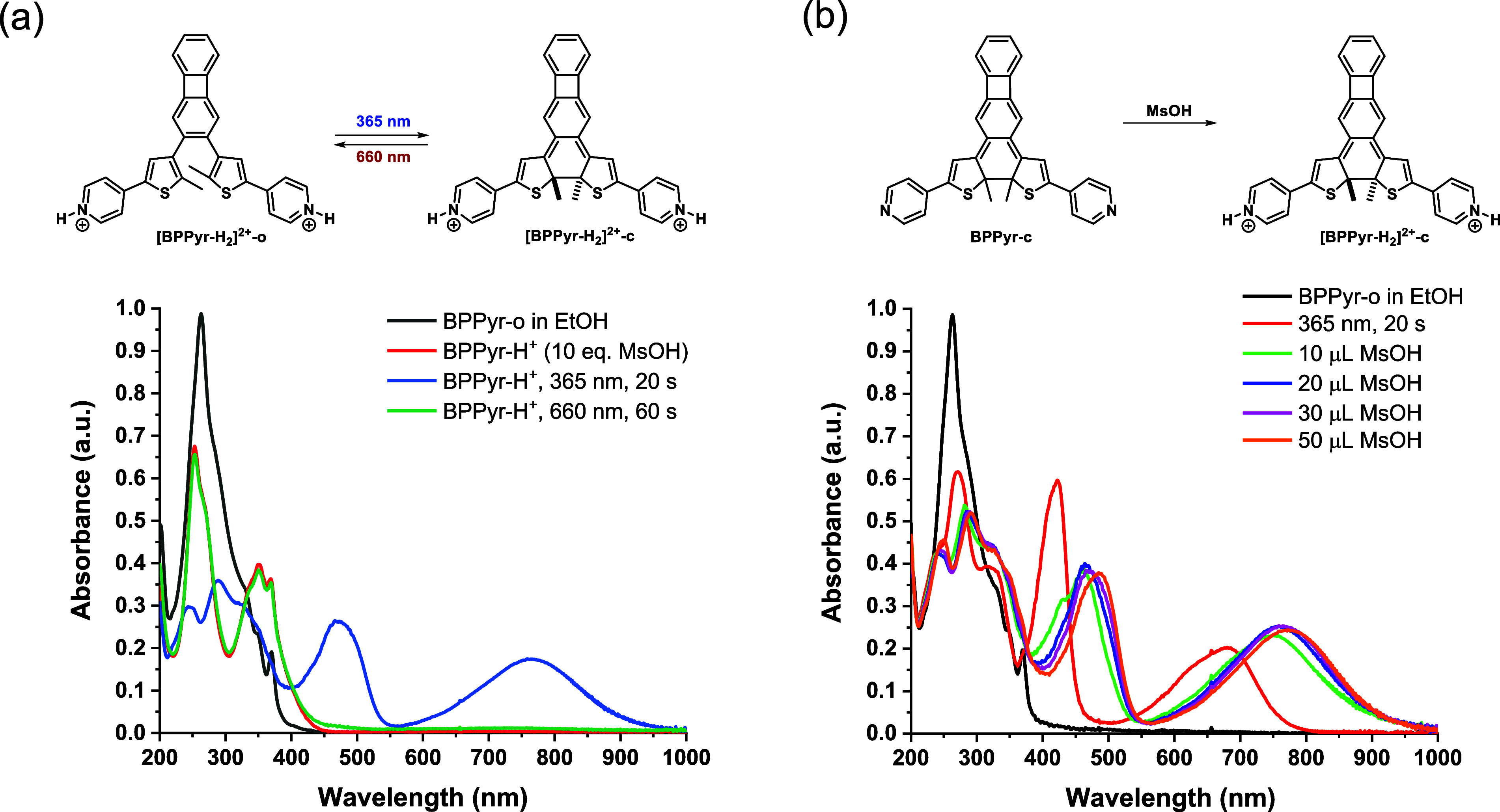
Changes in the UV–vis absorption of **BPPyr** in EtOH upon MsOH addition (*c* = 5 × 10^–5^ M). (a) Addition of 10 equiv. MsOH and (b) sequential addition of MsOH to **BPPyr-c** under UV irradiation.

The protonated **[BPPyr-H**_**2**_ **]** ^**2+**^ **-c** could also be generated from the neutral **BPPyr-c** by the addition of acid. The sequential addition of MsOH to a solution of **BPPyr-c** was followed by UV–vis spectroscopy (Figures 5b and S7, Supporting Information). A large red-shift of the absorption bands in the visible region occurred upon addition of 10 μL MsOH solution (3.3 equiv). This red-shift did not increase much upon further addition of acid. Next, ^1^H NMR spectroscopy provided structural insight into the protonation process. The addition of 1 equiv. MsOH to a solution of **BPPyr-o** in MeOD produced broad peaks in the aromatic region that are shifted downfield compared to the neutral species (Figure S8, Supporting Information). A somewhat smaller downfield shift was observed for the methyl substituents of the thiophene rings (Figure S9, Supporting Information). A further equivalent of acid led to further downfield shifts and somewhat sharper absorptions in the aromatic region that still contained “shoulders”. The addition of 3 equiv of acid led to sharp peaks that did not change further in the presence of 10 equiv. of acid. Based on these results, we conclude that 3 equiv. of MsOH is required to shift the protonation equilibrium toward the doubly protonated form of the switch.

A key finding from the protonation experiments is that protonation destabilizes the ring-closed isomer, which leads to much faster thermal ring opening than observed for the neutral compound. Thus, it further supports the possibility to use protonation as the energy-releasing mechanism for a MOST system based on a switch similar to **BPPyr**. Furthermore, the rate of the thermal ring opening of **BPPyr-c** was found to depend on the acid concentration. Specifically, kinetic investigations of this process in EtOH solution showed that already 1 equiv. MsOH reduces the *t*_1/2_ value considerably, from the original 2.9 h (at 25 °C) of the neutral species to about 14 min. Upon increasing the amounts of acid to 3 and 10 equiv., further reductions in the *t*_1/2_ value to 76 and 15 s, respectively, were observed (Figure 6 and Table S2, Supporting Information). This effect could be due to an associated increase in the polarity of the medium. However, alternative ring opening mechanisms not associated with pyridine protonation cannot be ruled out.^35^

**Figure 6:**
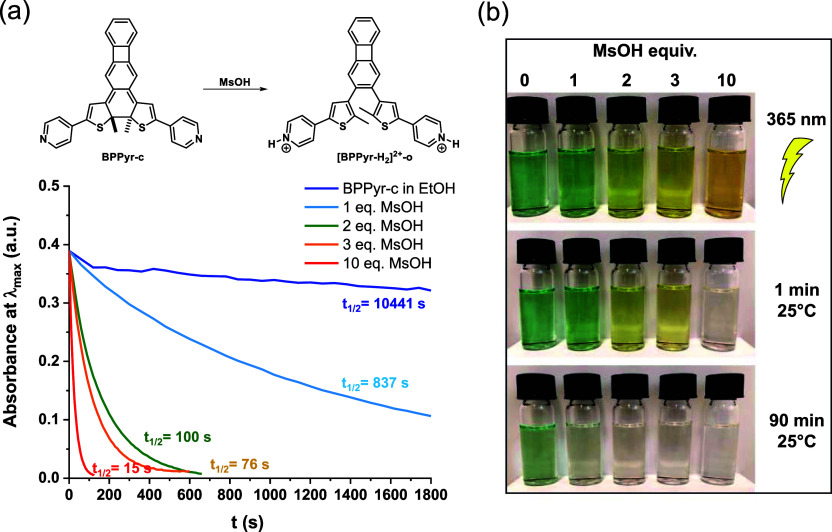
(a) Kinetics of the thermal ring opening of **BPPyr-c** in EtOH upon addition of MsOH at 25 °C and (b) color changes over time in the presence of different equivalents of MsOH following UV irradiation (365 nm, 10 s).

Finally, we note that the rate of the thermal ring opening of **[BPPyr-H**_**2**_ **]** ^**2+**^ **-c** showed only a moderate solvent dependence (Figure 7). Clearly, in the thermal destabilization of the closed isomer, the protonation is the dominant factor.

**Figure 7:**
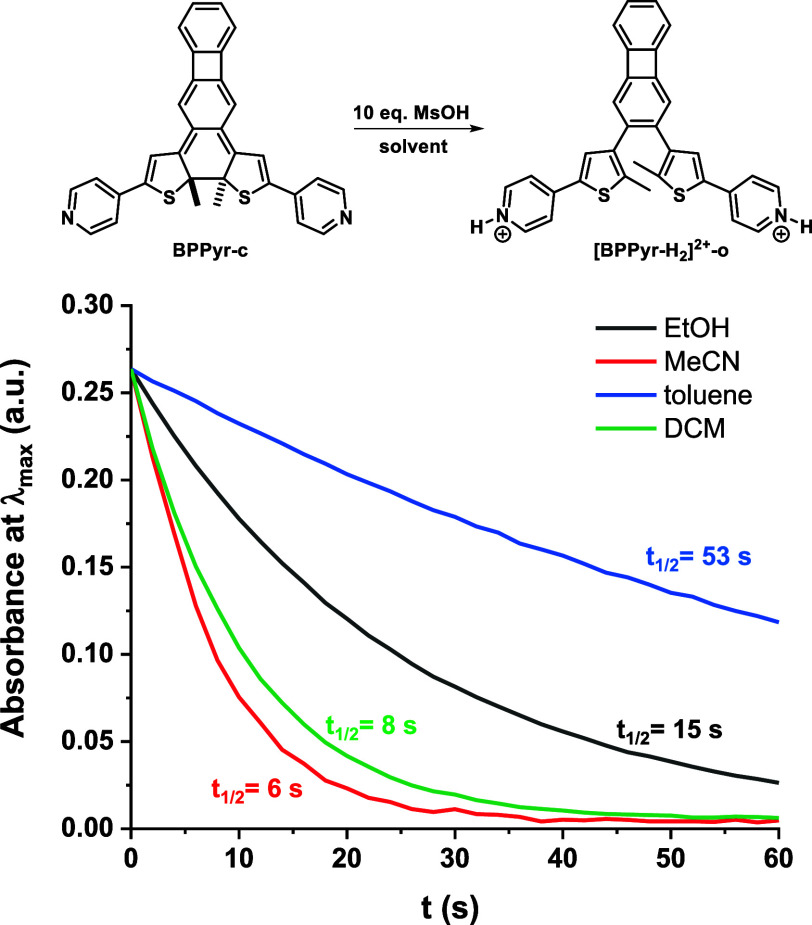
Solvent effect on the kinetics of the thermal ring opening of **[BPPyr-H**_**2**_ **]** ^**2+**^ **-c** at 25 °C.

### 2.4 Effect of Temperature on Thermal Ring Opening

Besides acid concentration and solvent effects, we also tested the influence of temperature on the stability of both **BPPyr-c** and **[BPPyr-H**_**2**_ **]** ^**2+**^ **-c** (Figure 8).

**Figure 8:**
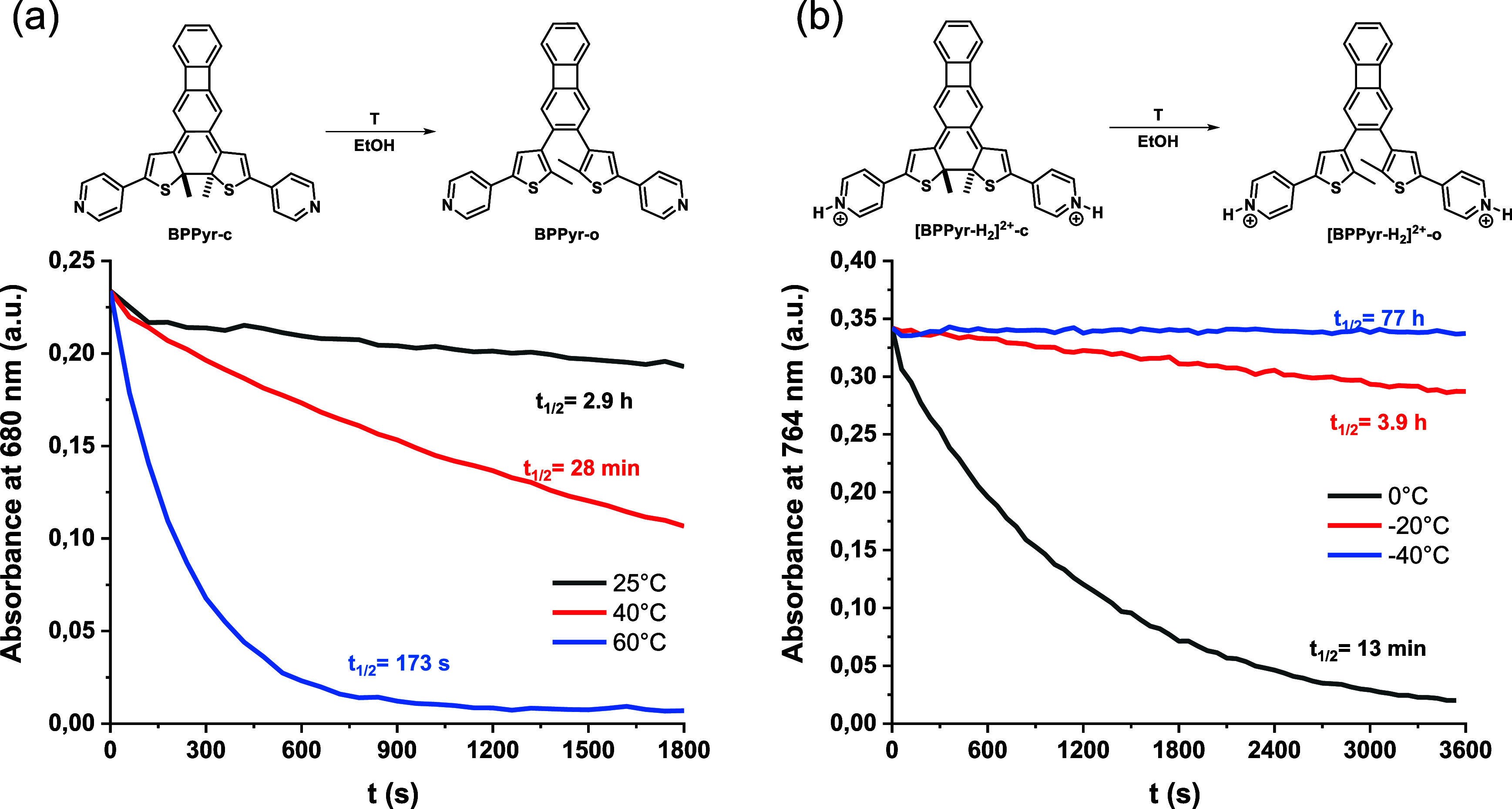
Temperature dependence of the thermal ring opening of (a) **BPPyr-c** and (b) **[BPPyr-H**_**2**_ **]** ^**2+**^ **-c** in EtOH.

Thereby, the *t*_1/2_ value of 2.9 h attributed to **BPPyr-c** in EtOH at 25 °C decreased to 28 and 3 min at 40 and 60 °C, respectively. Similarly, the *t*_1/2_ value of **[BPPyr-H**_**2**_ **]** ^**2+**^ **-c** could be increased by lowering the temperature. Specifically, its *t*_1/2_ value of 15 s in EtOH at 25 °C increased to 13 min, 3.9, and 77 h at 0, −20, and −40 °C, respectively. This shows that, apart from acid concentration and choice of solvent, temperature is another important factor through which the rate of thermal ring opening can be tuned.

### 2.5 Probing the Acid–Base Response

To further demonstrate the acid–base-responsive nature of **BPPyr**, a sequential irradiation/protonation/thermal relaxation/deprotonation experiment was conducted (Figure 9). The structural changes were monitored by UV–vis spectroscopy at 736 nm. At this wavelength, the intensities of the absorbances of **BPPyr-c** and **[BPPyr-H**_**2**_ **]** ^**2+**^ **-c** are equal. First, a solution of **BPPyr-o** in EtOH was heated up to 60 °C, which was practical for rapid thermal responses and for demonstrating the stability of the system at an elevated temperature. **BPPyr-o** was then irradiated with 365 nm light (30 s) to generate **BPPyr-c**. The formation of this isomer was apparent from the increasing absorption intensity at 736 nm. Subsequently, **BPPyr-c** was left in the dark for 60 s, which allowed for partial thermal relaxation to **BPPyr-o**, as indicated by the gradually decreasing absorption intensity. After 60 s in the dark, MsOH (10 equiv) was added to the solution. The resulting protonation destabilized the ring-closed form, which underwent rapid ring opening to form **[BPPyr-H**_**2**_ **]** ^**2+**^ **-o**. This process proceeded along with a loss of absorption at the monitoring wavelength. Subsequent UV irradiation/relaxation cycles led to rapid interconversion between **[BPPyr-H**_**2**_ **]** ^**2+**^ **-c** and **[BPPyr-H**_**2**_ **]** ^**2+**^ **-o**. The addition of excess Et_3_N (20 equiv) base to the solution converted **[BPPyr-H**_**2**_ **]** ^**2+**^ **-o** to **BPPyr-o**. Thereafter, repeated UV irradiation led to the thermally more stable **BPPyr-c**, as indicated by the slower decay of the absorption at 736 nm in the dark. With this step, the initial conditions of the experiment were reinstated. The rate of the thermal relaxation of **BPPyr-c** could be increased by acidifying the solution again.

**Figure 9:**
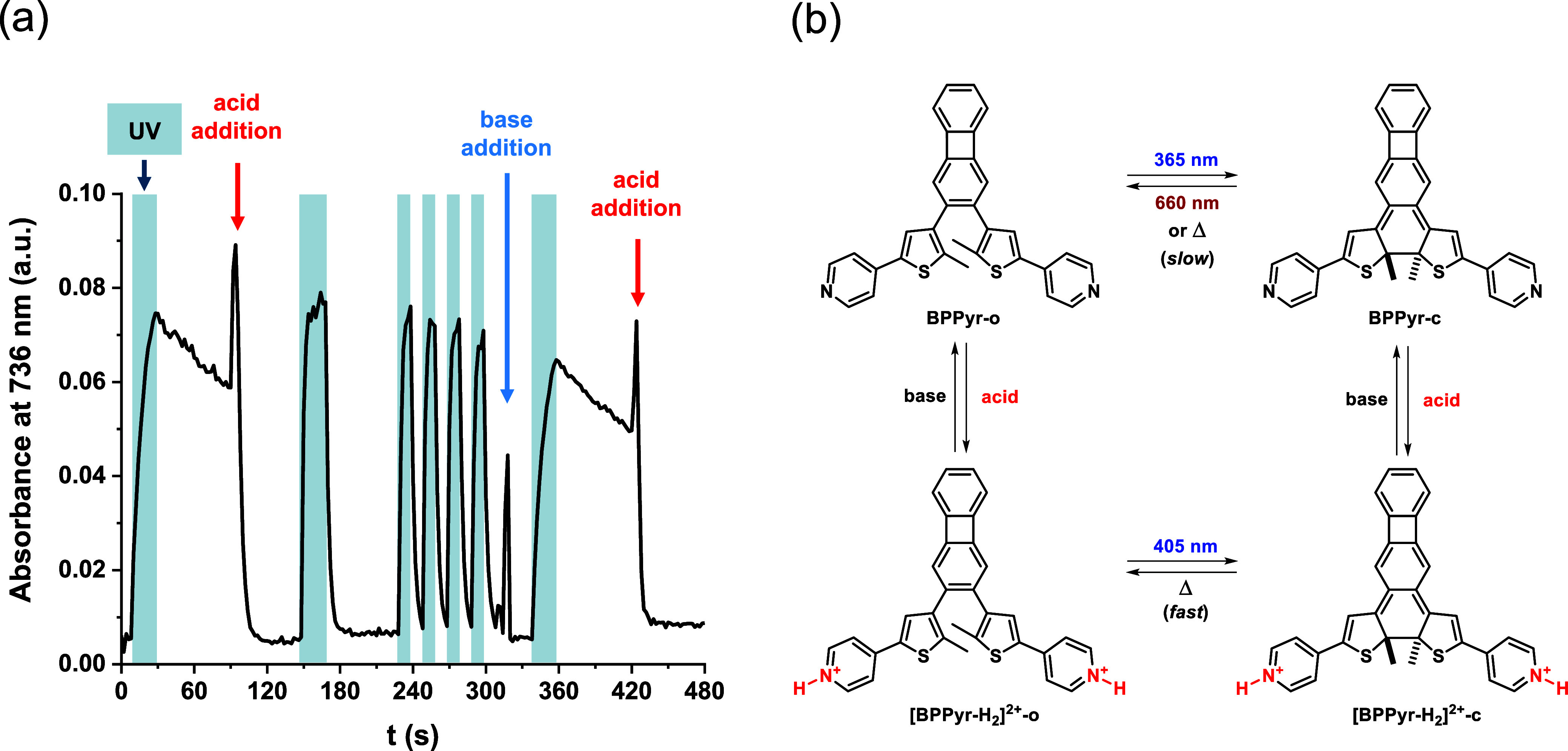
(a) Sequential 365 nm irradiation/protonation/thermal relaxation/deprotonation of **BPPyr** in EtOH at 60 °C. The addition of acid at 90 and 420 s corresponded to 10 and 50 equiv. of MsOH, respectively, and the addition of base corresponded to 20 equiv. of Et_3_N. (b) Summary of possibilities to control the multistimuli-responsive **BPPyr** system.

### 2.6 Fatigue Resistance of BPPyr and [BPPyr-H_2_]^2+^

Given that fatigue resistance is a key property for all practical applications of molecular switches,^36^ this property was explored for both **BPPyr** and **[BPPyr-H**_**2**_ **]** ^**2+**^. Upon alternating UV (365 nm, 10 s) and visible (620 nm, 20 s) irradiation of **BPPyr** (MeCN, 25 °C), or alternating UV irradiation (365 nm, 10 s) and dark relaxation (5 min) of **[BPPyr-H**_**2**_ **]** ^**2+**^ (EtOH, 25 °C), both compounds experienced only minor degradation within 20 isomerization cycles (Figure 10). To probe the limits of stability for both compounds, they were exposed to a continuous 365 nm irradiation, during which their absorbance changes were monitored by UV–vis spectroscopy (Figure S13–S17, Supporting Information). In these experiments, the combination of air and higher temperature (up to 50 °C) led to rapid degradation; however, this could be avoided under an inert atmosphere and a lower temperature. Based on data from these experiments, the degradation half-life time (*t* ^d^_1/2,_ the time elapsing under continuous irradiation before half of the molecules lose switchability) of **BPPyr** was calculated to be 10.1 min at 50 °C under aerobic conditions and 3.9 h at 20 °C under an argon atmosphere. Since 10 s of UV irradiation was found to be necessary for **BPPyr** to reach the PSS, a “switching half-life” (*s*_1/2_, the number of irradiation cycles after which half of the initially switchable molecules are lost) of 1399 cycles could be calculated under inert conditions (see also Section S2.1, Supporting Information). Protonation (10 equiv. MsOH) led to somewhat faster degradation upon continuous irradiation, as indicated by the decrease of the characteristic absorption band of **[BPPyr-H**_**2**_ **]** ^**2+**^ **-c** even under inert conditions (Figures S16 and S17, Supporting Information). With a *t* ^d^_1/2_ value of 1.98 h, an *s*_1/2_ value of 713 cycles could be calculated for **[BPPyr-H**_**2**_ **]** ^**2+**^ (Table S2, Supporting Information). Importantly, however, these values nonetheless agree quite well with those derived for **BPPyr**.

**Figure 10:**
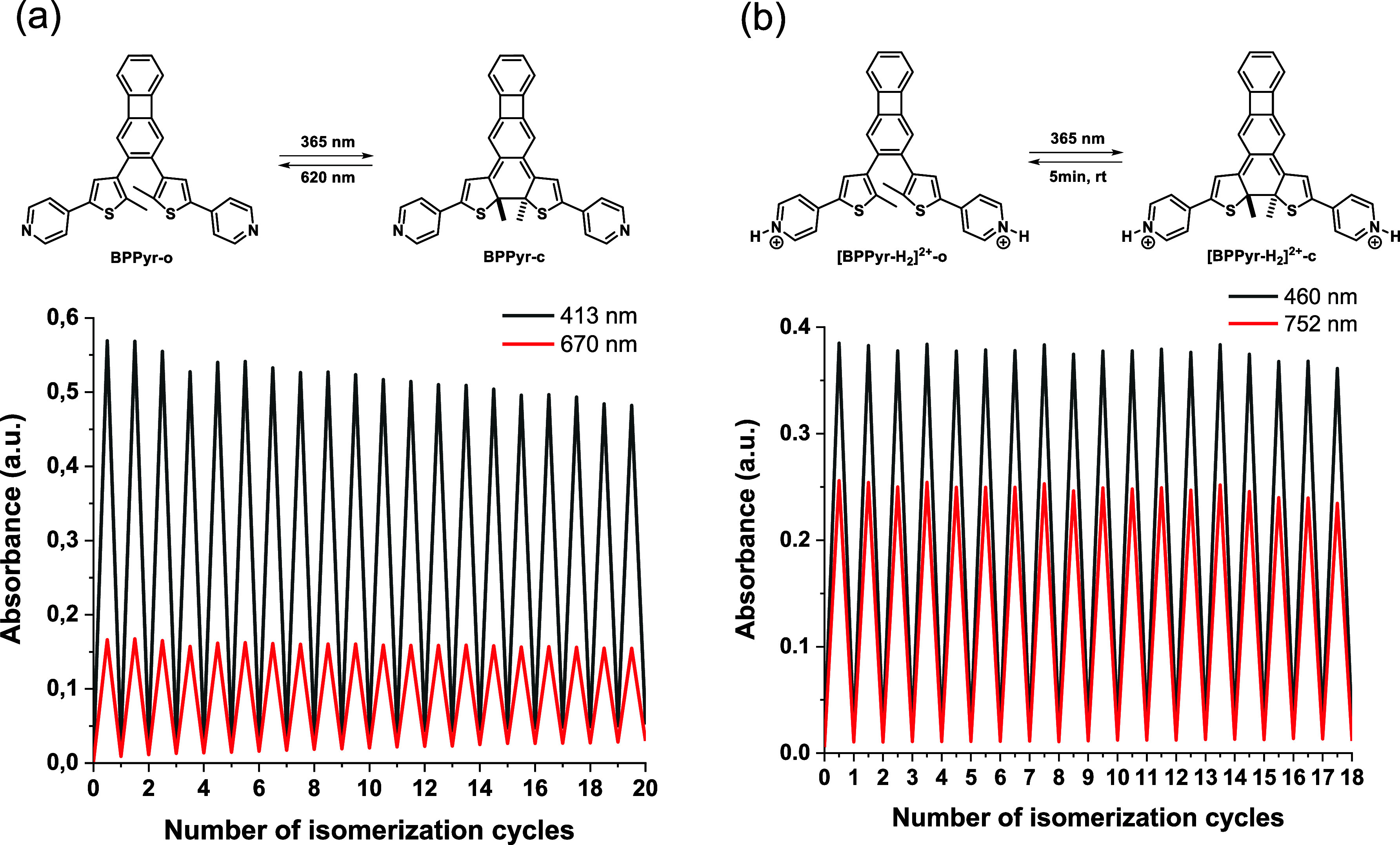
Fatigue resistance of (a) **BPPyr** (365 nm, 10 s/620 nm, 20 s, MeCN, 25 °C) and (b) **[BPPyr-H**_**2**_ **]** ^**2+**^ (365 nm, 10 s/dark 5 min, EtOH, 25 °C) monitored at the characteristic wavelengths.

### 2.7 Effects of *N*-Methylation

As a complementary structure, we prepared the bipyridinium salt of **BPPyr** by *N*-methylation of its pyridine rings. The resulting compound, **[BPPyr-Me**_**2**_ **]** ^**2+**^, showed similar properties as the protonated derivative **[BPPyr-H**_**2**_ **]** ^**2+**^. Reversible photoswitching was observed in this case as well; however, for both isomers (**[BPPyr-Me**_**2**_ **]** ^**2+**^ **-o)** and (**[BPPyr-Me**_**2**_ **]** ^**2+**^ **-c**), the absorption peaks were slightly bathochromically shifted compared to the corresponding protonated species (Figure 11a). Similarly to **[BPPyr-H**_**2**_ **]** ^**2+**^ **-o**, **[BPPyr-Me**_**2**_ **]** ^**2+**^ **-o** responded to 405 nm irradiation, reaching maximum absorption intensity at λ_max_ in 5 s in EtOH. The *t*_1/2_ value for the thermal ring opening of **[BPPyr-Me**_**2**_ **]** ^**2+**^ **-c** was found to be 13 s in EtOH, 6 s in MeCN, and 3 s in DCM at 25 °C (Table S3, Supporting Information). Notably, these estimates are virtually identical to those obtained for **[BPPyr-H**_**2**_ **]** ^**2+**^ **-c** in the presence of 10 equiv. of MsOH.

**Figure 11:**
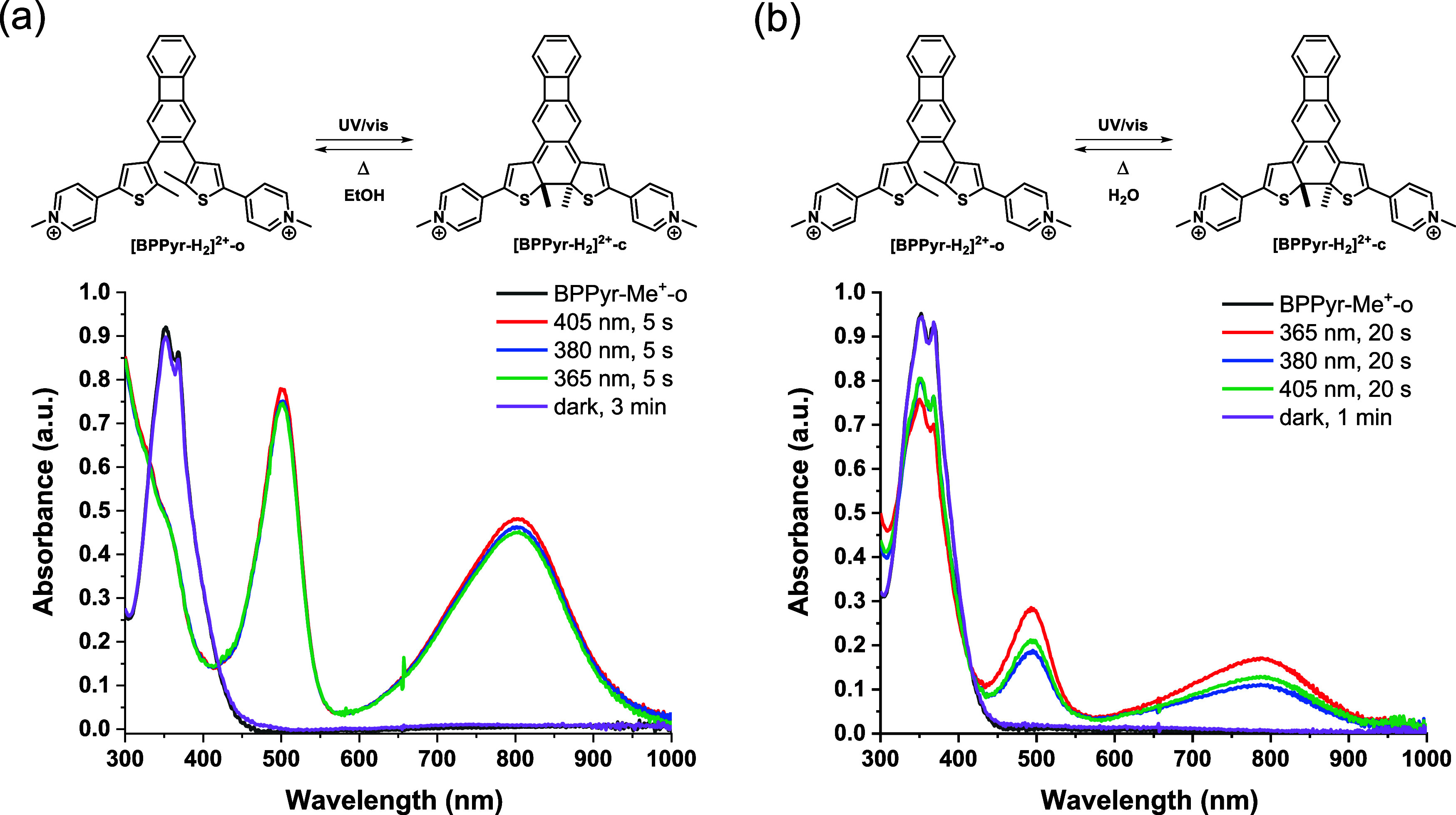
Molar absorbance spectra of **[BPPyr-Me**_**2**_ **]** ^**2+**^ in (a) EtOH and (b) water before and after light irradiation at 25 °C.

It should be highlighted that **[BPPyr-Me**_**2**_ **]** ^**2+**^ is water-soluble and preserves its visible-light switchability in aqueous media (Figure 11b). This is in contrast to **[BPPyr-H**_**2**_ **]** ^**2+**^, which underwent degradation when the organic solvents were replaced with water (Figure S18, Supporting Information). **[BPPyr-Me**_**2**_ **]** ^**2+**^ requires an irradiation time of 20 s to reach the PSS in water, and the comparably lower-intensity absorption peak around 789 nm suggests a smaller amount of the ring-closed form at the PSS than what **[BPPyr-H**_**2**_ **]** ^**2+**^ achieves. The *t*_1/2_ value for **[BPPyr-Me**_**2**_ **]** ^**2+**^ **-c** in water was found to be 4 s at 25 °C.

Interestingly, **[BPPyr-Me**_**2**_ **]** ^**2+**^ showed fatigue resistance comparable to that of **[BPPyr-H**_**2**_ **]** ^**2+**^ (Figures S11, S15 and S16c,d, Supporting Information). Specifically, continuous UV irradiation (365 nm) in EtOH under argon at 25 °C yielded a *t* ^d^_1/2_ value of 1.7 h. However, using 405 nm irradiation, an improved *t* ^d^_1/2_ value of 4.8 h was obtained, from which an *s*_1/2_ value of 3472 cycles could be derived (here, the irradiation time was 5 s). In the context of future MOST applications, the higher fatigue resistance achieved through irradiation with visible light is a very pertinent property of **[BPPyr-Me**_**2**_ **]** ^**2+**^.

To investigate whether **[BPPyr-Me**_**2**_ **]** ^**2+**^ can also be reversibly isomerized between its open and closed forms in a confined environment, which is likely a requirement for it to function in an actual device implementing the MOST concept,^42–44^ **[BPPyr-Me**_**2**_ **]** ^**2+**^ was embedded in a gelatin hydrogel. Gelatin,^50^ a biopolymer prepared via partial hydrolysis of collagen, consists of oligopeptide chains and has been tested for a variety of energy-storage applications,^51,52^ among many other potential areas of applications.^53,54^ First, a photopatterning experiment was conducted at room temperature using a 5 mW laser light source emitting at 405 nm. A benzene ring was hand-drawn in the gel, which gradually disappeared within 2 min, after which the original light-yellow color of the gel was recovered (Figure 12a). The same experiment was repeated by using a mask on top of the gel, while natural sunlight through a glass window was used as a light source (Figure 12b). Notably, the rate of thermal ring opening appeared slower in the viscous hydrogel (Figure 12c) compared to the previously described solution-based experiments (*t*_1/2_ = 4 s in water). Nevertheless, both the laser and sunlight induced fast and reversible photoisomerization of the hydrogel-embedded **[BPPyr-Me**_**2**_ **]** ^**2+**^ molecules, which demonstrate their potential to sustain continuous energy transformation within a confined polar environment.

**Figure 12:**
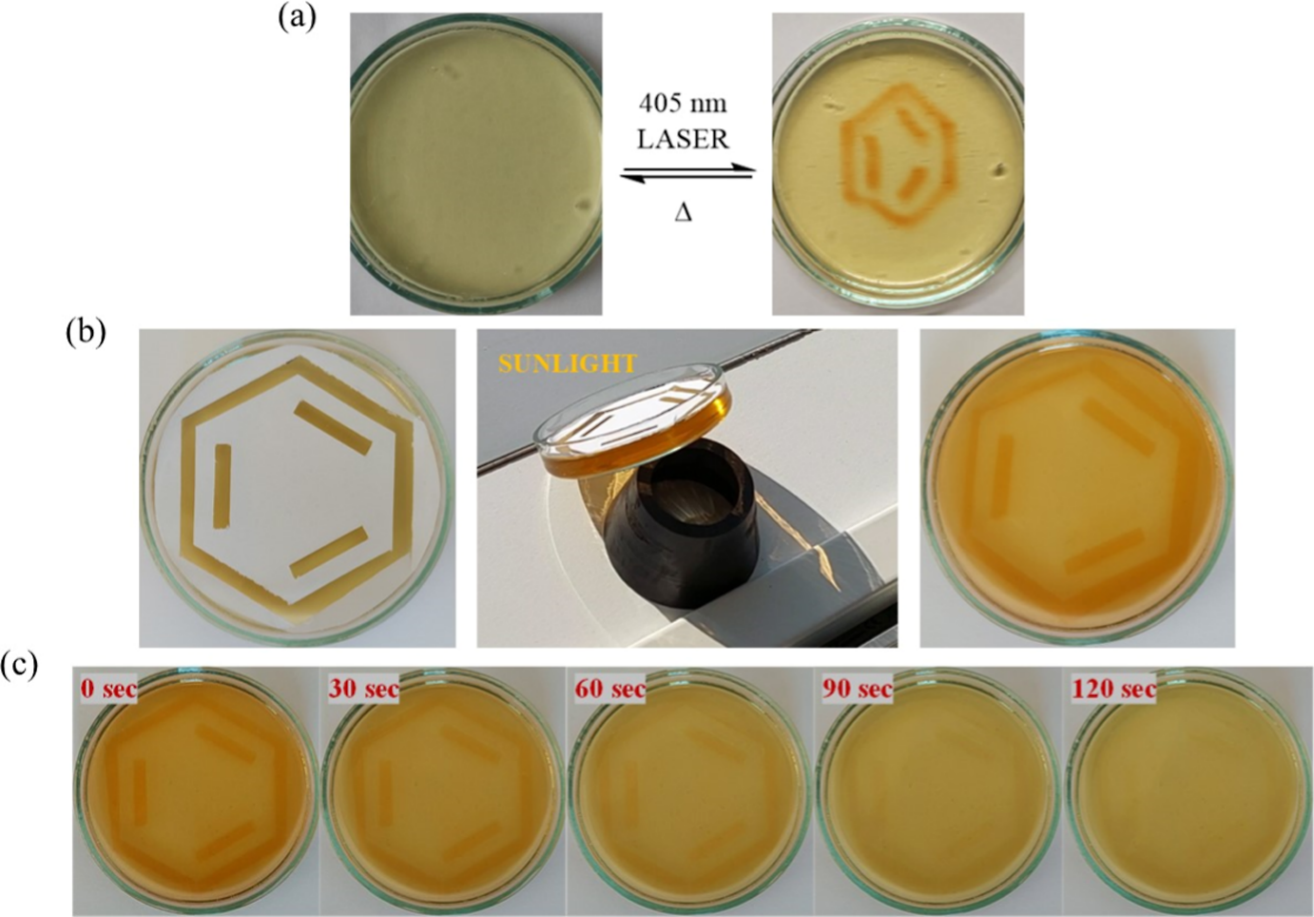
Photopatterning experiments with **[BPPyr-Me**_**2**_ **]** ^**2+**^ in a gelatin hydrogel. (a) Photopatterning of the hydrogel with a 405 nm laser. (b) Photopatterning of the hydrogel through a mask using natural sunlight (the scattered light causes a slight coloration of the covered part of the gel). (c) Thermal ring opening of **[BPPyr-Me**_**2**_ **]** ^**2+**^ **-c** in the hydrogel. The patterning experiment could be repeated multiple times without apparent loss of photoresponse.

### 2.8 Quantum Chemical Calculations

The experiments in Figure 6 show that the barrier for the thermal ring opening of **BPPyr-c** decreases when the likelihood for protonation of the pyridine units increases. This finding, which is supported by the observation that the half-life time for ring opening of **[BPPyr-Me**_**2**_ **]** ^**2+**^ **-c** is as small (13 s in EtOH) as that of **BPPyr-c** in the presence of 10 equiv. of MsOH (15 s in EtOH), further highlights the possibility^20,22,35^ to release the energy stored in the ring-closed isomer through acid catalysis. Thus, switches like **BPPyr** hold one key advantage over many other types of photoswitches employed for MOST applications in that the energy-releasing step may be triggered without using an expensive transition-metal catalyst. In this light, it is natural to try to gain an understanding of this mode of catalysis in simple, quantitative terms, which is possible through quantum chemical modeling. To this end, density functional theory (DFT) methods were used to calculate free-energy barriers for the thermal ring opening in the current **BPPyr** switches, as well as in other biphenylene-based dithienylarene switches appended by different groups than pyridine. All switches for which this was done are shown in Figure 13. Here, it may be noted that, with greater ease than experiments would offer, the calculations allow us to distinguish changing the protonation states of both groups added to the thienyl units, from changing just one of the protonation states. The technical details of the calculations, performed with the Gaussian 16 suites of programs,^55^ are given in Section S1 of the Supporting Information, which also includes a motivation why the calculations were carried out with the M06-2X^56^ and ωB97X-D^57^ hybrid density functionals.

**Figure 13:**
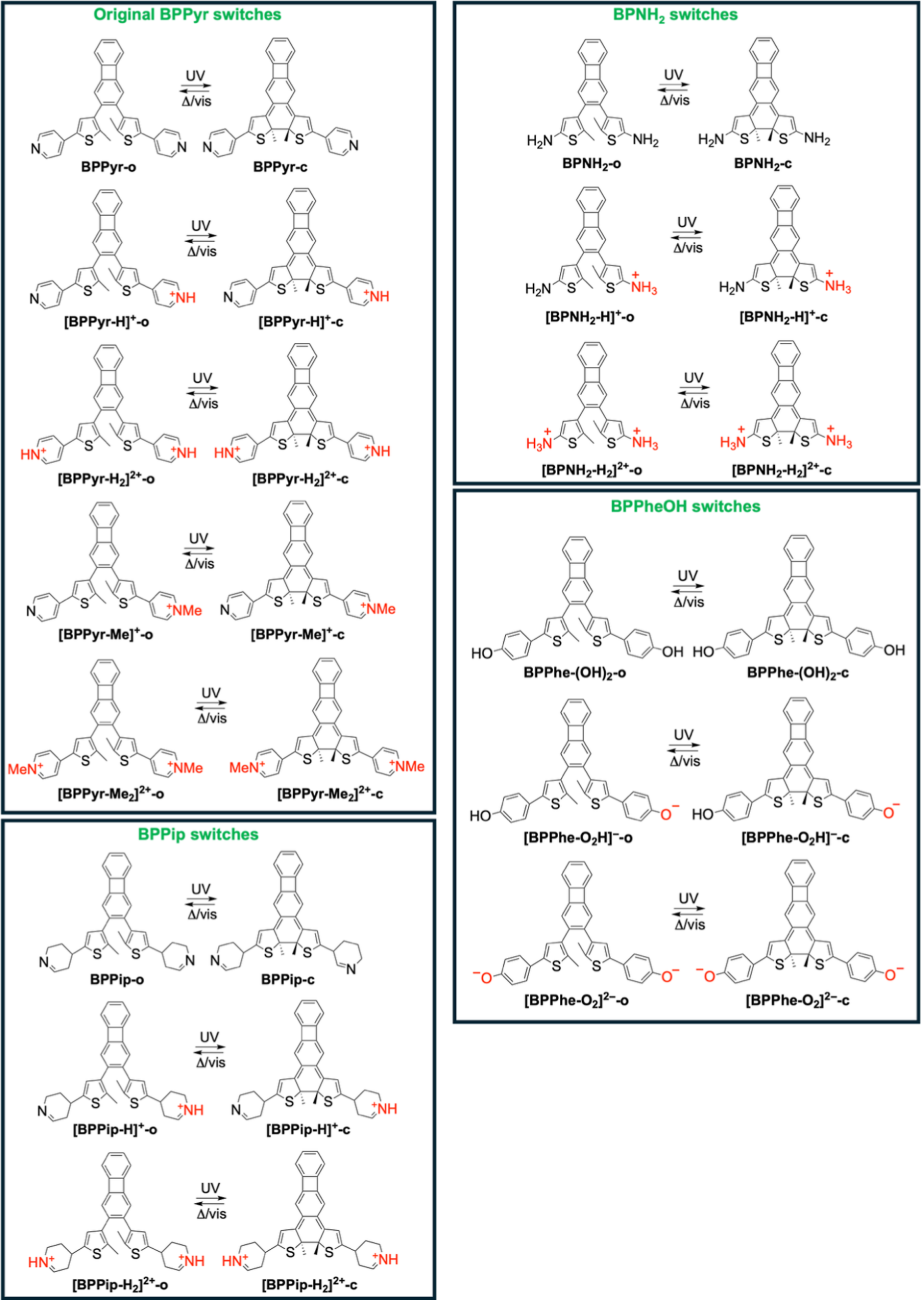
Open and closed forms of all switches for which DFT calculations were performed.

Using a continuum description^58^ of an EtOH solvent, the results of the calculations are summarized in Table 3. Starting with the original **BPPyr** switches, it is pleasing to note that the corresponding calculations reinforce the experimental observations by showing that protonation/methylation lowers the barrier for ring opening quite significantly. Specifically, when both pyridine units are protonated or methylated, the barrier is reduced by 25–29 (M06-2X) or 19–23 kJ mol^–1^ (ωB97X-D). As a possible explanation for this effect, it has been proposed that it may reflect a destabilization of the ring-closed isomer when electron-withdrawing capability is incorporated in conjugation with the photochromic core.^22^ In order to test this hypothesis quantitatively, calculations were also performed on switches (denoted **BPPip**) in which the pyridines are replaced by 1-piperideines and, consequently, the conjugation is broken. For these switches, protonating both rings now reduces the barrier by a negligible amount, 2–7 kJ mol^–1^, which is a clear indication that the hypothesis is correct. Further support for this scenario comes from two additional sets of calculations. First, replacing the pyridines with amino groups instead of 1-piperideines (yielding the **BPNH**_**2**_ switches in Figure 13 and Table 3) results in a substantial 30–31 kJ mol^–1^ reduction of the barrier when protonation of both groups now introduces electron-withdrawing capability directly at the photochromic core. Second, replacing the pyridines with phenols (the **BPPheOH** switches in Figure 13 and Table 3) conversely raises the barrier by 15–17 kJ mol^–1^ when deprotonation of these groups instead provides electron-donating character in conjugation with the photochromic core.

**Table 3:** Free-Energy Barriers for Thermal Ring Opening (Δ*G*_b_^‡^, kJ mol^–1^) of Different Switches and Their Thermal Electrocyclization Reaction Free Energies (Δ*G*_r_, kJ mol^–1^) and Energy-Storage Densities (Δ*G*_s,_ MJ kg^–1^) Calculated Using the M06-2X and ωB97X-D Density Functionals^a^

switch	M06-2X				ωB97X-D			
	Δ*G*_b_^‡^	Δ*G*_f_^‡^	Δ*G*_r_	Δ*G*_s_	Δ*G*_b_^‡^	Δ*G*_f_^‡^	Δ*G*_r_	Δ*G*_s_
original **BPPyr** switches								
**BPPyr**	94.3	194.3	100.0	0.20	84.0	187.3	103.3	0.21
**[BPPyr-H]** ^**+**^	76.0		101.3	0.20	69.9		107.8	0.22
**[BPPyr-H**_**2**_ **]** ^**2+**^	65.1		106.9	0.21	61.0		119.1	0.24
**[BPPyr-Me]** ^**+**^	81.0		99.4	0.19	72.5		108.2	0.21
**[BPPyr-Me**_**2**_ **]** ^**2+**^	69.0		104.0	0.20	64.6		116.8	0.22
**BPPip** switches								
**BPPip**	121.6		86.4	0.17	105.8		97.9	0.19
**[BPPip-H]** ^**+**^	122.4		85.5	0.17	102.3		104.0	0.21
**[BPPip-H**_**2**_ **]** ^**2+**^	119.6		89.5	0.18	98.7		102.6	0.20
**BPNH**_**2**_ switches								
**BPNH**_**2**_	144.2	191.7	47.5	0.13	131.3	187.2	55.9	0.15
**[BPNH**_**2**_ **-H]** ^**+**^	–^b^		60.2	0.16	121.1		69.3	0.18
**[BPNH**_**2**_ **-H**_**2**_ **]** ^**2+**^	114.7		99.1	0.26	100.7		102.8	0.27
**BPPheOH** switches								
**BPPhe-(OH)**_**2**_	108.5		85.6	0.16	92.5		96.1	0.18
**[BPPhe-O**_**2**_ **H]** ^**–**^	115.0		68.1	0.13	–^b^		79.0	0.15
**[BPPhe-O**_**2**_ **]** ^**2–**^	123.2		58.7	0.11	109.7		69.0	0.13

a For the **BPPyr** and **BPNH**_**2**_ switches, free-energy barriers for thermal ring closing (Δ*G*_f_^‡^, kJ mol^–1^) are also given.

b A transition structure for thermal ring opening could not be located.

As evidence that the results in Table 3 are internally consistent, it may be noted that the changes in barrier heights when altering the protonation states of both pyridine/amino/phenol moieties are invariably larger than those when just one of the moieties is modified. Furthermore, it can also be seen that, without exception, M06-2X yields larger barriers than ωB97X-D, which is consistent with observations that a functional containing a large fraction of exact exchange (M06-2X: 54%) typically gives barriers exceeding those predicted by a functional containing a smaller fraction (ωB97X-D: 22% at short interelectronic distances).^59,60^

Having explored the possibility to trigger the thermal ring opening of **BPPyr-c** through acid catalysis, it is also of interest to assess the key MOST performance indicator that the calculated energy-storage densities of the different **BPPyr** switches constitute.^29^ Obtained as the ratio between the thermal electrocyclization reaction free energies and the molecular weights, these values are also included in Table 3. As can be seen, regardless of the protonation/methylation state, they fall in a narrow range of about 0.20–0.25 MJ kg^–1^, which is slightly lower than the often-quoted target value of 0.30 MJ kg^–1^ for MOST applications.^29^ Nonetheless, given that the 100–103 kJ mol^–1^ reaction energy calculated for the neutral **BPPyr** switch is very close to that of 101 kJ mol^–1^ previously calculated for the “parent” **BPMe** switch (which holds methyl groups instead of pyridines),^17^ the key factor for the lowering of the energy-storage density of **BPPyr** appears to be its high weight. Thus, it might be possible to improve this density by simply finding a smaller group than pyridine with the same functionality. Here, it should be noted from Table 3 that replacing the pyridines with amino groups is not a viable strategy in this regard, leading instead to a decrease in the density (from 0.20–0.21 MJ kg^–1^ in neutral **BPPyr** to 0.13–0.15 MJ kg^–1^ in neutral **BPNH**_**2**_), despite the associated reduction in weight. Consequently, it must hold that the amino groups either strongly destabilize the ring open isomer of **BPNH**_**2**_, or strongly stabilize the ring-closed isomer, or that a combined effect is at play. In order to distinguish between these possibilities, it is useful to compare the already-discussed ring opening barriers for **BPPyr** and **BPNH**_**2**_, with the corresponding ring closing barriers, which are also included in Table 3. Thereby, it can be seen that while the ring closing barriers for **BPPyr** and **BPNH**_**2**_ are similar to within 0–3 kJ mol^–1^, the ring opening barriers differ by a considerable 47–50 kJ mol^–1^ (with **BPNH**_**2**_ showing the larger ones). Thus, the main reason why the amino groups decrease the energy-storage density of **BPNH**_**2**_ relative to **BPPyr** appears to be that they stabilize the ring-closed isomer.

## 3 Conclusions

In summary, we have designed and synthesized a pyridine-appended, biphenylene-bridged dithienylarene switch, **BPPyr**, and explored a number of different aspects of its performance and suitability as an active component in future MOST systems. While the lower aromaticity of the benzene motifs of the biphenylene provides thermal stability to the closed form, as compared to when the thienyl units instead are bridged by just benzene itself, the appended pyridines are found to offer an efficient route toward thermal ring opening, and hence energy release, through protonation of their basic N atoms (**[BPPyr-H**_**2**_ **]** ^**2+**^). Furthermore, we demonstrate that the thermal stability of the closed form is also controllable through the choice of the solvent. Crucially, the negative effect on fatigue resistance exerted by protonation is marginal, which further supports the merits of the acid–base-controlled energy release by dithienylarene-based MOST systems. Methylation of the N atoms of the pyridines gives access to a water-soluble switch, **[BPPyr-Me**_**2**_ **]** ^**2+**^, that exhibits similarly good fatigue resistance and undergoes rapid thermal ring opening. Moreover, this compound is compatible with the polar environment within a gelatin hydrogel, in which it preserves its photoresponse even under natural sunlight irradiation. Given that MOST chromophores immobilized in soft materials are likely to be useful in the future development of novel device architectures for energy conversion and storage, this is an important result. Finally, by using DFT methods to calculate free-energy barriers for thermal ring opening in **BPPyr** switches with different levels of protonation/methylation, as well as for other biphenylene-based dithienylarene switches appended by other groups than pyridine, the experimental observations as to the effect of protonation/methylation are both reinforced and explained. Specifically, the lowering of the ring opening barrier upon protonation/methylation is attributed to a destabilization of the ring-closed isomer when an electron-withdrawing capability is incorporated in conjugation with the photochromic core of the switches.

## Supplementary Material







## Notes

Experimental details, additional spectroscopic and kinetic data, NMR spectra, and details of DFT calculations (PDF)
Laser (vid1) (MP4)
Solar-induced (vid2) photopatterning experiments of the photoresponsive gelatin (MP4)
