# Supplementary material for: Fatigue-Resistant Dithienylarene Photoswitches with Acid-Regulated Thermal Ring Opening
Source: ACS Omega. 2026 May 18;11(21):31357–70. doi: 10.1021/acsomega.6c01422 (PMC13234642; doi:10.1021/acsomega.6c01422)
Supplement: Supplementary file 1 [file ao6c01422_si_001.pdf]

Supporting Information

Fatigue-Resistant Dithienylarene Photoswitches with Acid-Regulated Thermal Ring Opening

Attila Kunfi,[a,*] Barnabás Zsignár-Nagy,[a] D. Sravanakumar Perumalla,[b,c,*] Bo Durbeej,[b,*] Gábor London[a,*]

[a] Institute of Organic Chemistry, HUN-REN Research Centre for Natural Sciences, 1117 Budapest, Magyar tudósok krt. 2, Hungary
[b] Division of Theoretical Chemistry, IFM Linköping University, SE-58183 Linköping, Sweden
[c] Department of Applied Sciences and Humanities, Sasi Institute of Technology & Engineering, Tadepalligudem-534 101, India

Email addresses:
attila.kunfi@ttk.hu (A.K.)
sravanakumar.perumalla@sasi.ac.in (D.S.P.)
bo.durbeej@liu.se (B.D.)
london.gabor@ttk.hu (G.L.)

Table of Contents

S1 General Information 2
S2 Complementary Spectroscopic and Kinetic Data 4
S2.1 Calculation of the thermal half-life times, the degradation half-life times, and the switching half-lives of the photoswitches 15
S2.1.1 Kinetics studies of the thermal ring opening reaction 15
S2.1.2 Thermal ring opening of **BPPyr-c** to **BPPyr-o** at different temperatures in EtOH 16
S2.1.3 Thermal ring opening of **[BPPyr-H$_2$]$_2$$^+$-c** to **[BPPyr-H$_2$]$^{2+}$-o** at different temperatures in EtOH 17
S2.1.4 Ring opening kinetics of **[BPPyr-H$_2$]$^{2+}$-c** to **[BPPyr-H$_2$]$^{2+}$-o** at different H^+ equivalents in EtOH at 25°C 18
S2.2 Quantum yield determination for the light-induced ring closing and ring opening reactions of BPPyr 18
S3 Synthetic Procedures 20
S4 NMR Spectra 24
S5 References 32
S6 Electronic Energies and Cartesian Coordinates of Calculated Structures 33

S1 General Information

Commercial reagents, solvents, and catalysts (Sigma-Aldrich, Fluorochem, VWR) were purchased as reagent grade and used without further purification. Solvents for extraction or column chromatography were of technical quality. Organic solutions were concentrated by rotary evaporation at 40 °C. Thin-layer chromatography (TLC) was carried out on 'Merck silica gel 60 F_{254}' or 'Merck aluminium oxide 60 F_{254} neutral' type UV-active silica or alumina sheets. Column chromatography was performed using a Teledyne Isco CombiFlash® Rf+ automated flash chromatography system with 'RediSep R_f Gold®' silica gel filled column or with 'RediSep Gold® C18 Reversed-Phase' column at 25(±1) °C. The cartridge was filled with Zeochem® 'ZEOprep 60 25-40 µm' silica gel.

Analytical RP-HPLC-UV/Vis-MS measurements were carried out using a Shimadzu LCMS-2020 instrument applying a Gemini C18 column (100 x 2.00 mm I.D.) in which the stationary phase is 5 µm silica with a pore size of 110 Å. The chromatograms were detected by a UV–vis diode array (190-800 nm) and an ESI-MS detector. The following linear gradient elution profile was applied: 0 min 0 % B; 6.5 min 100% B; 7 min 0% B; 8 min 0% B, with eluent A (2% HCOOH, 5% CH_3CN and 93% water) and B (2% HCOOH, 80% CH_3CN and 18% water) at a flow rate of 0.8-mL/min at 40 °C. Room temperature (r.t.) refers to 25(+/-1)°C.

NMR spectra were acquired on a Varian 500 (^{1}H 500 MHz, ^{13}C 126 MHz) and a Varian 300 (^{1}H 300 MHz, ^{13}C 75 MHz) NMR spectrometer. The residual solvent peaks were used as the internal reference. Chemical shifts (δ) are reported in ppm. The following abbreviations are used to indicate the multiplicity in ^{1}H NMR spectra: s, singlet; d, doublet; t, triplet; and m, multiplet. ^{13}C NMR spectra were acquired on a broad-band decoupled mode. All NMR spectra were recorded at 30 °C unless stated otherwise.

UV–Vis absorption spectroscopy was executed on a PerkinElmer Lambda 465 spectrophotometer. Data were collected from 1100 to 190 nm using a photodiode array detector. Hellma Analytics high-precision quartz cuvettes were used with optical path length of 1.0 cm. Solutions were prepared in the concentration range of $1 – 5 \times 10^{-5}$ M at r.t. Baseline correction was used for every solvent. Temperature controlled UV–Vis measurements were conducted using an Unisoku CoolSpeK USP-203 cryostat.

High-resolution mass spectrometry measurements were performed on a Sciex TripleTOF 5600+ high-resolution tandem mass spectrometer equipped with a DuoSpray ion source. APCI or ESI ionization was applied in the positive ion detection mode. Samples were dissolved in acetonitrile and flow injected into the acetonitrile/water 1:1 flow. The flow rate was 0.2 mL/min. The resolution of the mass spectrometer was 35000.

Irradiation of samples were carried out with 10 W and 30 W COB LED light sources, placed 2 cm from the samples and operated at a current of 1.0 A unless stated otherwise. The photon fluxes of the LEDs at the sample were the following: 10 W 365 nm, 65 mW/cm^2; 30 W 365

nm, 175 mW/cm^2; 30 W 380 nm, 113 mW/cm^2; 30 W 405 nm, 146 mW/cm^2; 30 W 620 nm, 198 mW/cm^2. Samples for irradiation experiments were prepared under dimmed lighting conditions and kept in a light excluding container until each measurement. The laser pen used in the photopatterning experiments was a 10 mW, 405 nm commercially available standard laser pointer.

The free-energy barriers for the thermal ring opening in the different switches in Figure 13 of the main text were calculated by optimizing the geometries of the open and closed forms of each switch, and the transition structure connecting the two forms, using the M06-2X[1] and ωB97X-D[2] hybrid density functionals. The optimizations were carried out with the cc-pVTZ basis set and by employing the integral equation formulation of the polarizable continuum model (PCM)[3] to simulate an EtOH solvent. Based on the resulting geometries, frequency calculations were performed at the same levels of theory to derive Gibbs free energies at room temperature, and to verify that any given optimized structure corresponds to either a potential-energy minimum (having real vibrational frequencies only) or a transition structure (having one imaginary vibrational frequency). All calculations were done with the Gaussian 16 suites of programs.[4]

The choice to perform the calculations with the M06-2X and ωB97X-D hybrid density functionals was informed by several factors. For example, it is generally a good idea to use functionals from different categories, because in the event that the chosen functionals support the same set of conclusions, which is the case in this study, it is then less likely that the conclusions are biased by the specific choice. In this regard, the fact that M06-2X and ωB97X-D have very different key features is a major advantage. Specifically, M06-2X is a so-called global hybrid meta-GGA that includes a fixed 54% fraction of exact exchange, as well as a dependence on the kinetic energy density.[1] ωB97X-D, in turn, is a so-called range-separated hybrid GGA that includes a variable fraction of exact exchange, from 22% at short interelectronic distances to 100% at large.[2] Another reason for using M06-2X and ωB97X-D is their proven advantage over other functionals in describing dispersion interactions,[1,2,5] such as those likely to be present between the two thienyl units of the **BPPyr** and related switches studied herein. Moreover, M06-2X and ωB97X-D have also been shown to perform well in predicting barrier heights,[5] which is particularly pertinent for the purposes of this work.

S2 Complementary Spectroscopic and Kinetic Data

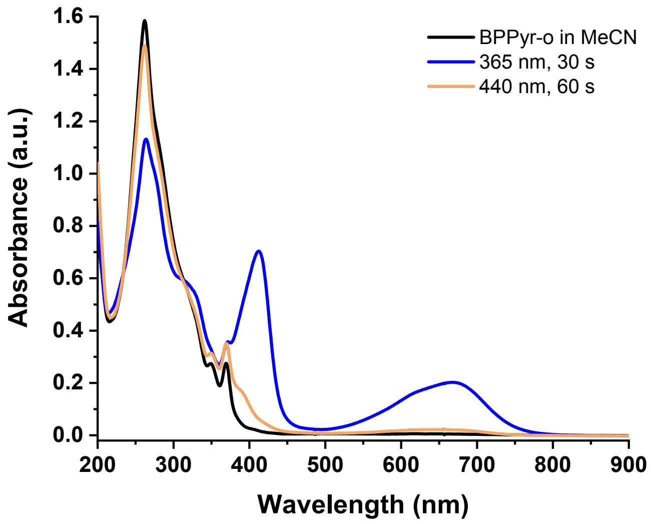

Figure S1. UV-vis spectra of **BPPyr** in MeCN before and after irradiation with different wavelengths of light. The measurements were conducted under aerobic conditions using 10 W COB LED light sources. The presence of air resulted in a noticeable degree of degradation as indicated by a new band around 400 nm after 440 nm light irradiation.

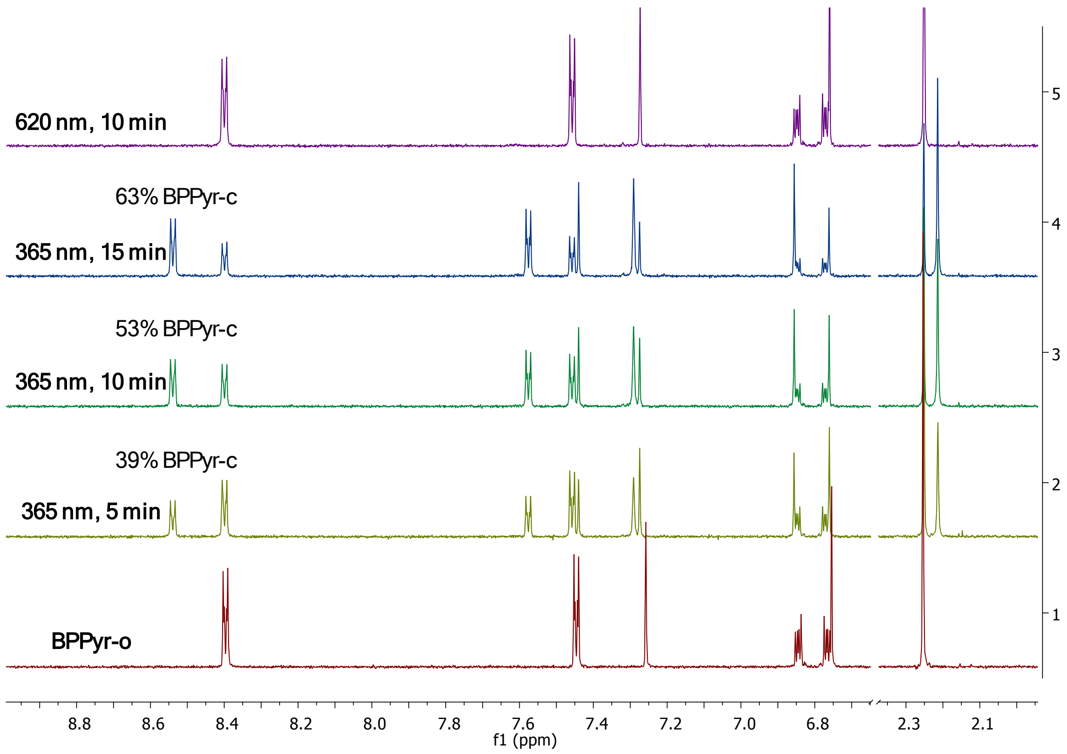

Figure S2. ^{1}H-NMR (500 MHz) spectra of **BPPyr-o** before and after UV (365 nm) and visible (620 nm) light irradiations (10 W LED) in MeOD at 25°C.

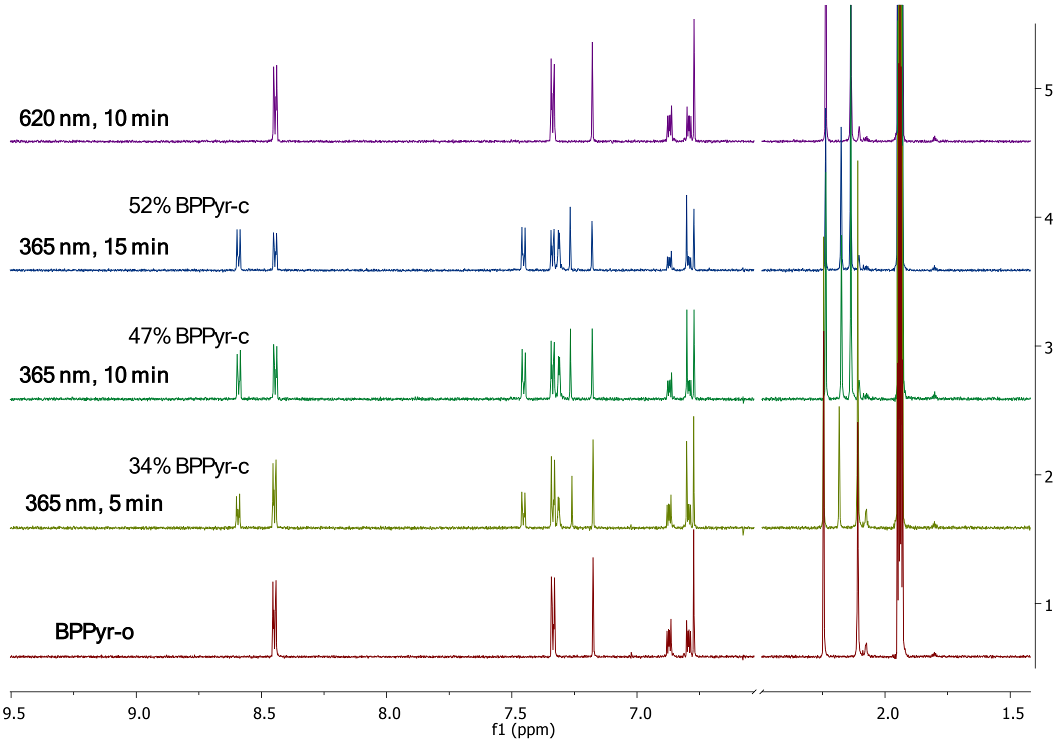

Figure S3. ^{1}H-NMR (500 MHz) spectra of **BPPyr-o** before and after UV (365 nm) and visible (620 nm) light irradiations (10 W LED) in CD_3CN at 25°C.

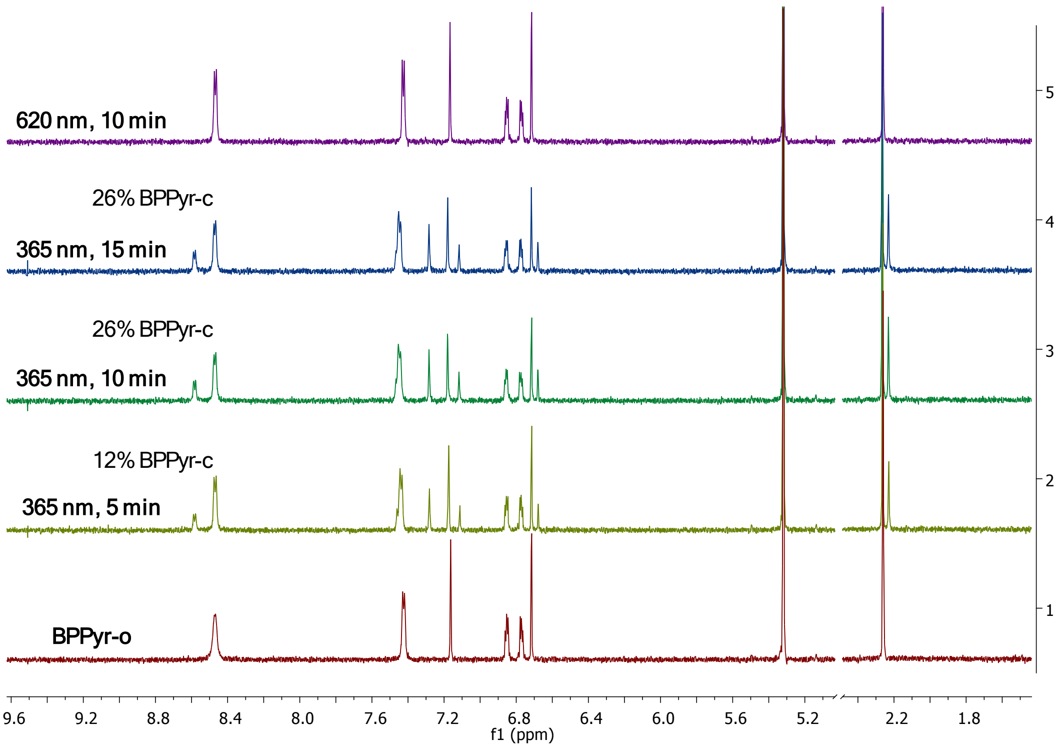

Figure S4. ^{1}H-NMR (500 MHz) spectra of **BPPyr-o** before and after UV (365 nm) and visible (620 nm) light irradiations (10 W LED) in CD_2Cl_2 at 25°C.

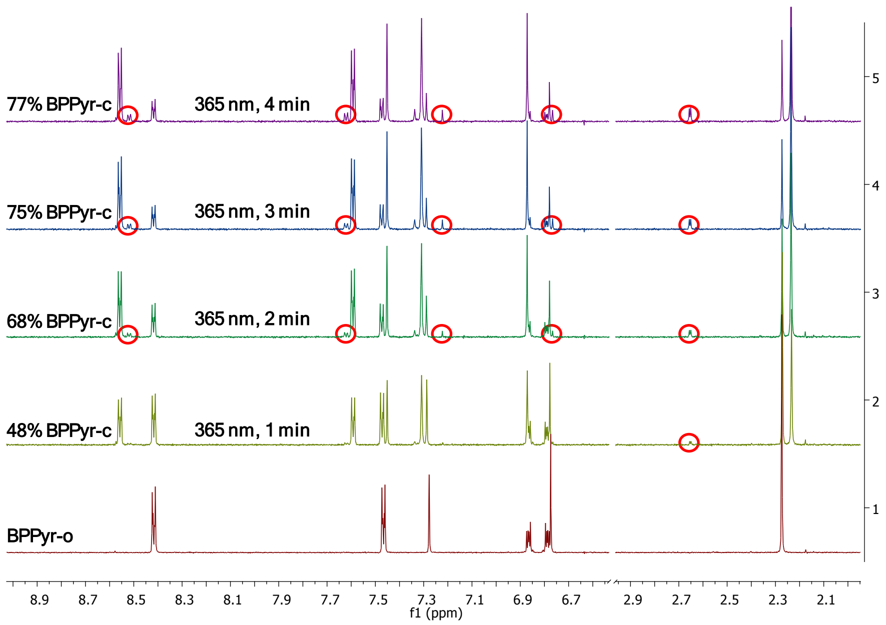

Figure S5. ^{1}H-NMR (500 MHz) spectra of **BPPyr-o** before and after UV (365 nm) irradiation using a 30 W LED light source in MeOD. The NMR tube was kept in a 25°C water bath during the irradiation. New peaks that correspond to an irreversible side-product are indicated with red circles.

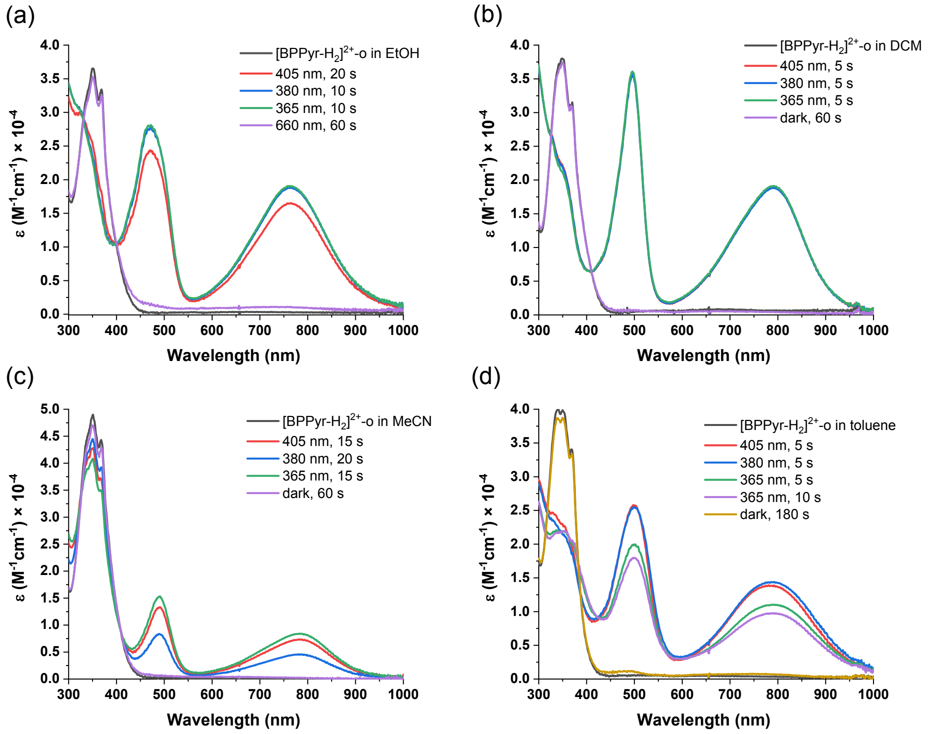

Figure S6. (a)-(d) UV-vis spectra of **$[BPPyr\text{-}H_2]^{2+}$** (in the presence of 10 eq. $MeSO_3H$) in different solvents before and after irradiation with specified wavelengths of light. The duration of the irradiation was set to reach the PSS (no further spectral change upon irradiation).

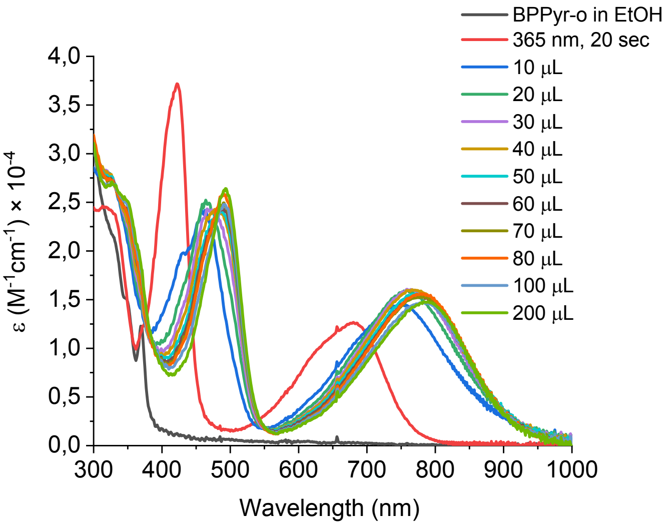

Figure S7. Effect of the amount of acid on the absorption spectrum of **BPPyr-c** in EtOH. A volume of 30 µL of 1.044 × 10^{-2} M $MeSO_3H$ solution equals 10 eq H^+. The sample solution was irradiated with 365 nm light (30 W LED) for 20 s after each acid addition to reach the PSS for the measurement.

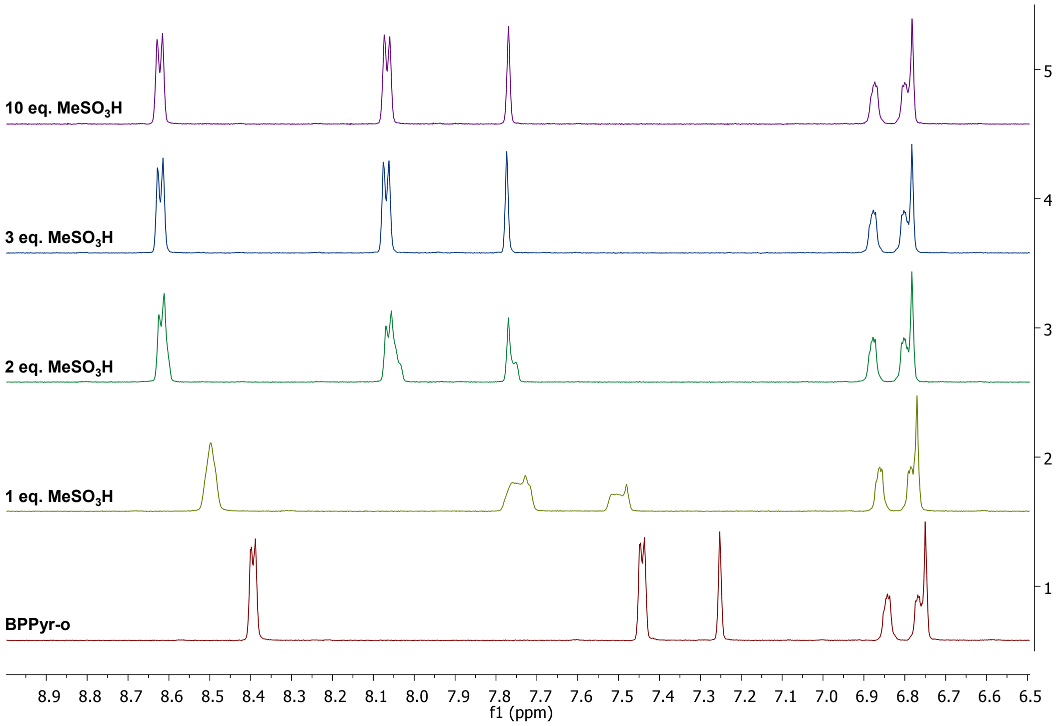

Figure S8. Changes in the aromatic region of the ^{1}H-NMR (500 MHz, MeOD) spectra of **BPPyr-o** upon addition of different amounts of $MeSO_3H$.

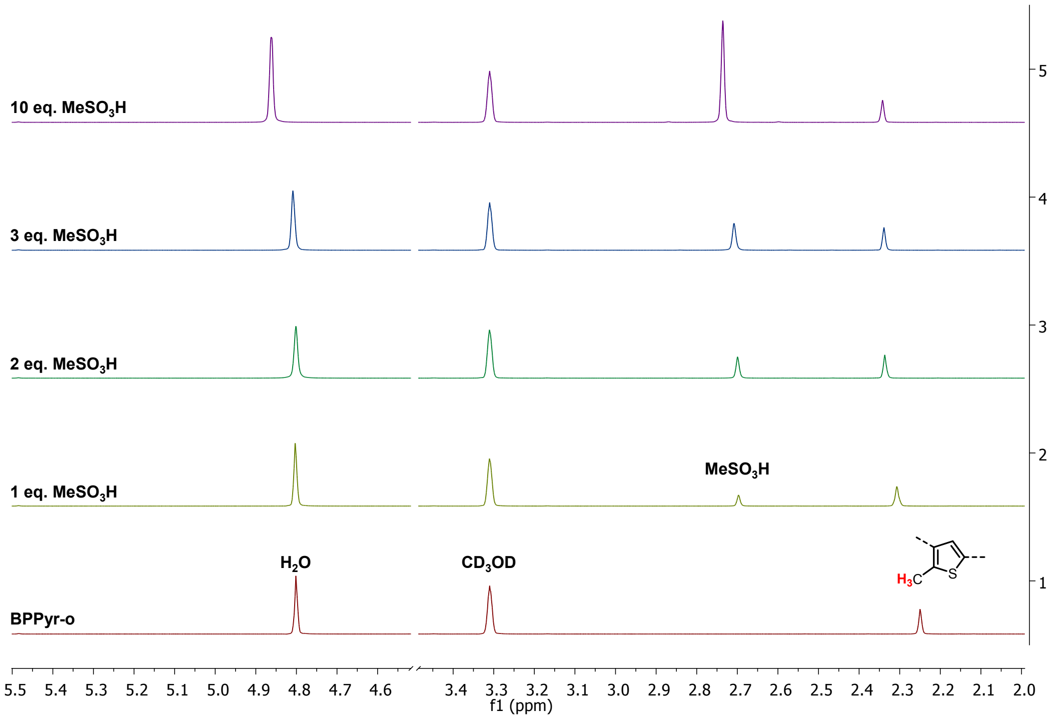

Figure S9. Changes in the alkyl region of the ^{1}H-NMR (500 MHz, MeOD) spectra of **BPPyr-o** upon addition of different amounts of $MeSO_3H$.

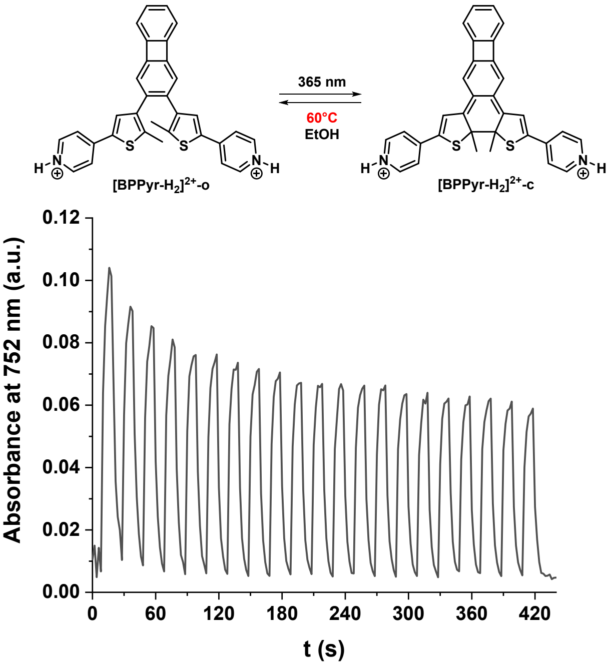

Figure S10. Absorbance changes at 752 nm during switching cycles of **$[BPPyr-H_2]^{2+}$** (365 nm for 10 s, then thermal ring opening at 60°C in EtOH).

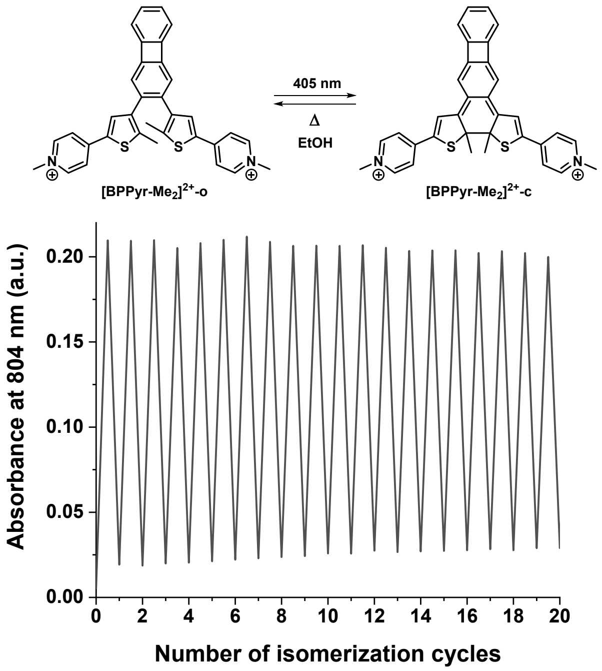

Figure S11. Absorbance changes at 804 nm during switching cycles of **$[BPPyr\text{-}Me_2]^{2+}$** (405 nm for 5 s, then thermal ring opening in the dark for 60 s at 25°C in EtOH; measurements were carried out under Ar atmosphere).

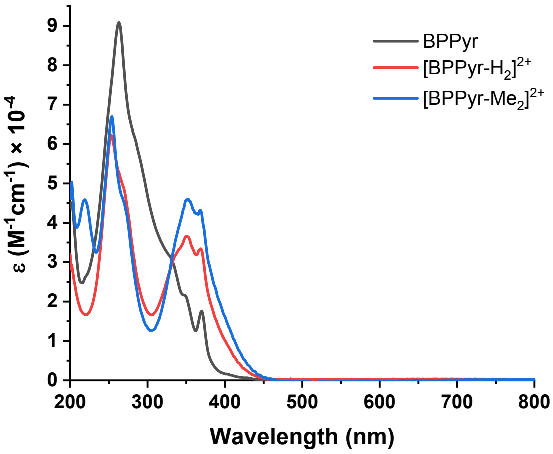

Figure S12. Molar absorbance spectra of **BPPyr-o**, **$[BPPyr\text{-}H_2]^{2+}$-o**, and **$[BPPyr\text{-}Me_2]^{2+}$-o** in EtOH.

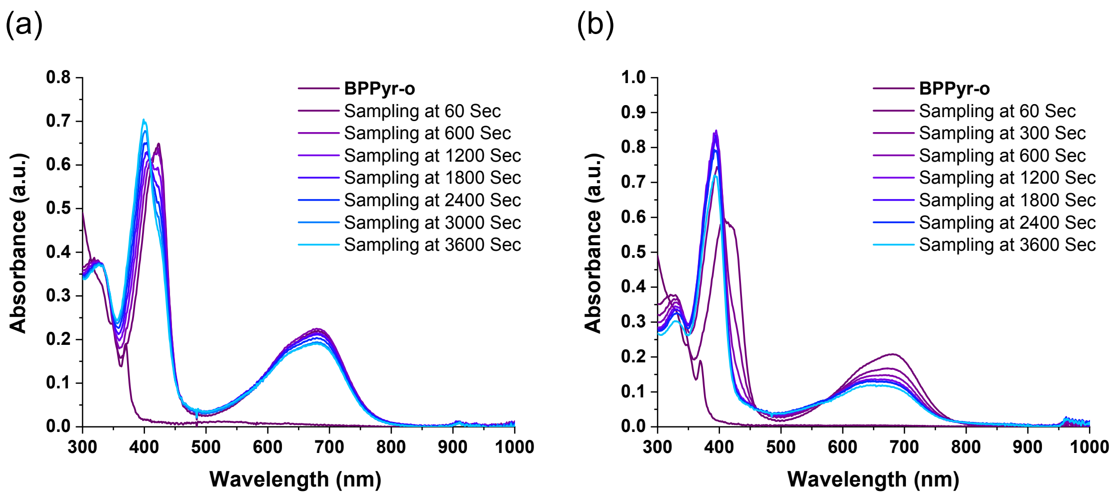

Figure S13. Monitoring the changes in the UV-vis spectrum of **BPPyr** during continuous irradiation under different conditions. (a) Ar atmosphere, 20°C, 365 nm, EtOH; (b) Air, 50°C, 365 nm, EtOH.

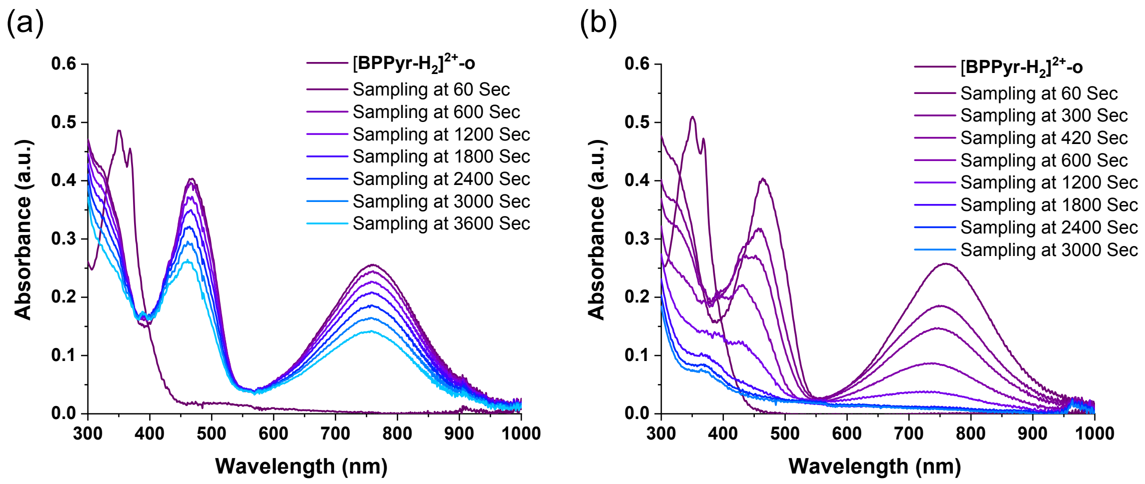

Figure S14. Monitoring the changes in the UV-vis spectrum of **$[BPPyr\text{-}H_2]^{2+}$** during continuous irradiation under different conditions. (a) Ar atmosphere, 20°C, 365 nm, EtOH; (b) Air, 50°C, 365 nm, EtOH.

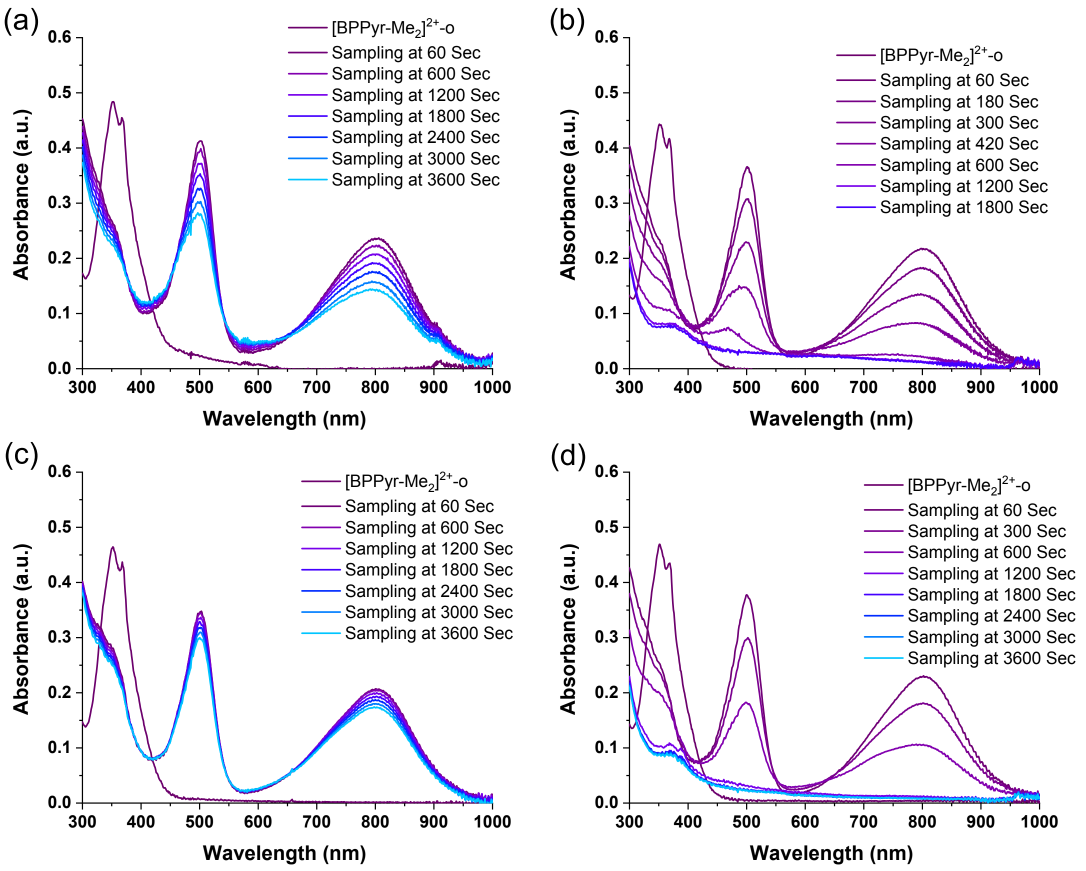

Figure S15. Monitoring the changes in the UV-vis spectrum of **[BPPyr-Me$_2$]$^{2+}$** during continuous irradiation under different conditions. (a) Ar atmosphere, 20°C, 365 nm, EtOH; (b) Air, 50°C, 365 nm, EtOH; (c) Ar atmosphere, 20°C, 405 nm, EtOH; (d) Air, 50°C, 405 nm, EtOH.

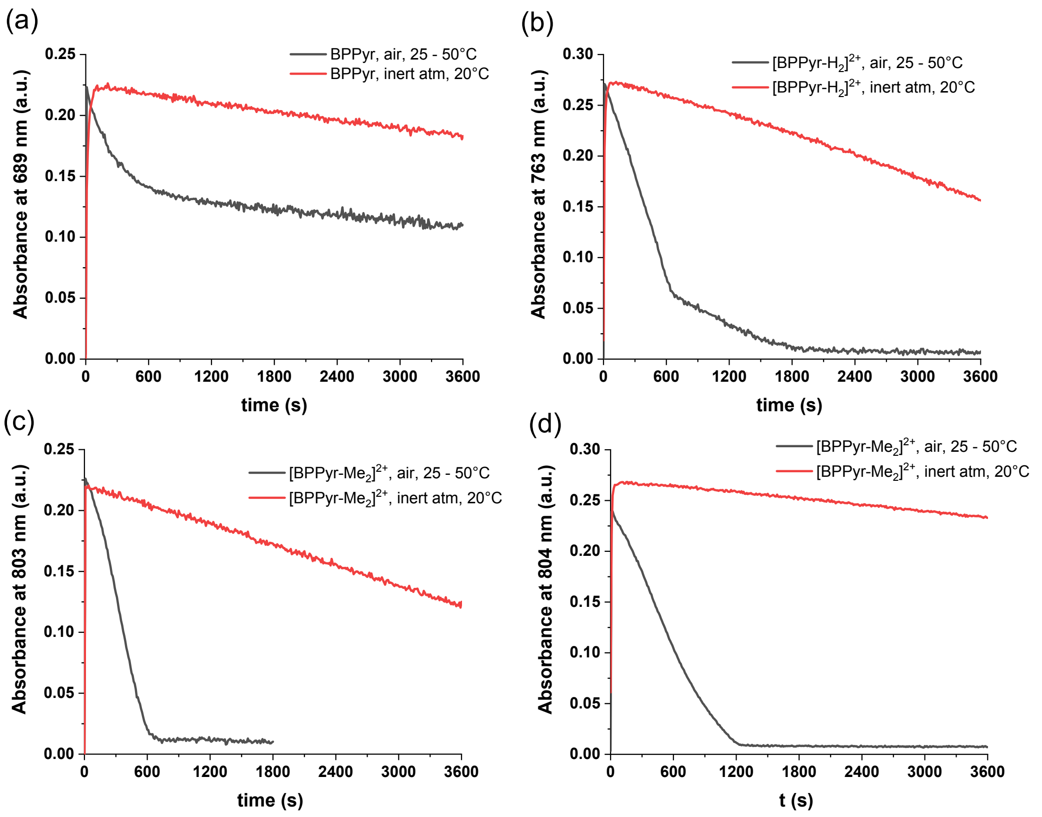

Figure S16. Absorbance changes at characteristic wavelengths during continuous 365 nm irradiation of the solutions of (a) **BPPyr**, (b) **[BPPyr-H$_2$]$^{2+}$**, (c) [**BPPyr-Me$_2$]$^{2+}$** in EtOH; and (d) continuous irradiation of [**BPPyr-Me$_2$]$^{2+}$** with 405 nm light under aerobic conditions and inert atmosphere. Note, that black curves also represent cases, when no temperature control was used, therefore the sample temperature reached 50°C within a few minutes from the beginning of irradiation due to the heating effect of the light source.

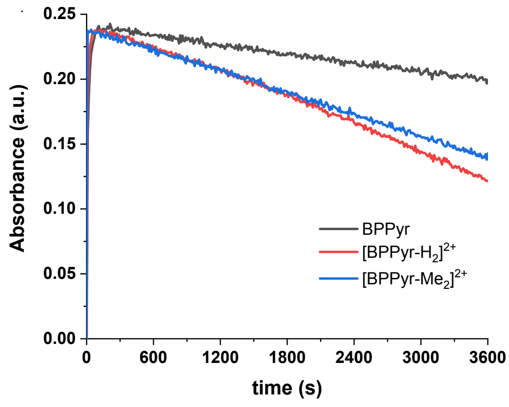

Figure S17. Changes at the long-wavelength absorption maxima of the different switches upon continuous 365 nm light irradiation. (EtOH was used as the solvent, the samples were purged with argon before the experiment, and the sample temperature was regulated to 20°C during irradiation.)

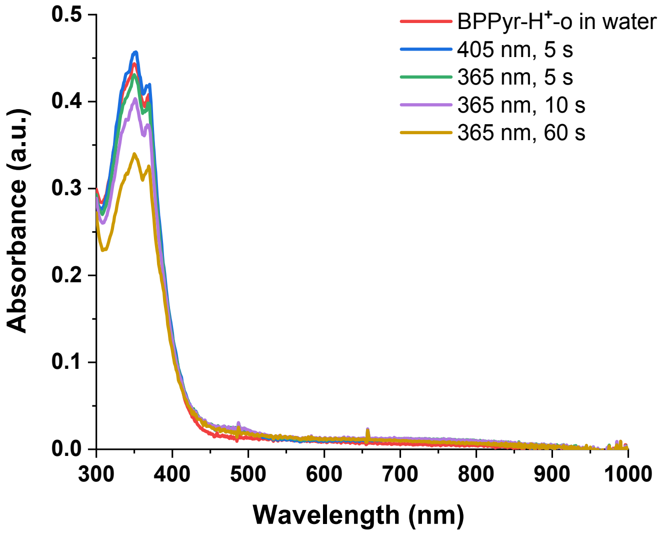

Figure S18. Loss of photoswitchability of **[BPPyr-H$_2$]$^{2+}$** in water. **BPPyr-o** was dissolved in DCM, 10 equiv. MsOH was added, and the organic solvent was removed at 25°C under vacuum. The resulting compound was dissolved in water. Its UV-vis spectrum was identical to the spectrum of **[BPPyr-H$_2$]$^{2+}$-o**, however no sign of photoisomerization was detected under 405 and 365 nm irradiation.

Table S1. Spectroscopic and kinetic data of **BPPyr** in different solvents.

Entry	Solvent	λ^{o}_{max} (nm)	ε^{o}_{max} ($M^{-1}cm^{-1}$)	λ^{c}_{max} (nm)	k (s^{-1})	$t_{1/2}^{25°C}$ (s)
1	EtOH	370	17600	421, 680	6.76×10^{-5}	10441
2	MeCN	369	16494	412, 667	6.42×10^{-5}	10805
3	DCM	371	17441	420, 678	1.90×10^{-3}	364
4	toluene	372	16273	422, 677	1.65×10^{-4}	4208

Table S2. Spectroscopic and kinetic data of **$[BPPyr\text{-}H_2]^{2+}$** in different solvents.

Entry	Solvent	λ^{o}_{max} (nm)	ε^{o}_{max} ($M^{-1}cm^{-1}$)	λ^{c}_{max} (nm)	k (s^{-1})	$t_{1/2}^{25°C}$ (s)
1	EtOH	351, 369	36535, 33408	472, 762	4.62×10^{-2}	15
2	MeCN	351, 368	49013, 44323	491, 783	1.24×10^{-1}	6
3	DCM	350, 370	37989, 31519	496, 792	9.03×10^{-2}	8
4	toluene	350, 369	39877, 34058	498, 792	1.31×10^{-2}	53

Table S3. Spectroscopic and kinetic data of **$[BPPyr\text{-}Me_2]^{2+}$** in different solvents.

Entry	Solvent	λ^{o}_{max} (nm)	ε^{o}_{max} ($M^{-1}cm^{-1}$)	λ^{c}_{max} (nm)	k (s^{-1})	$t_{1/2}^{25°C}$ (s)
1	water	352, 369	38082, 37110	493, 789	1.82×10^{-1}	4
2	EtOH	352, 368	46029, 43189	502, 804	5.18×10^{-2}	13
3	MeCN	350, 368	45830, 43341	459, 793	1.07×10^{-1}	6
4	DCM	355, 367	47383, 43776	531, 851	2.21×10^{-1}	3

Table S4. Spectroscopic and kinetic data of photoswitches under various conditions. The measurements were conducted under continuous irradiation of the ethanolic solutions of the photoswitches.

Entry	Photoswitch	Conditions (atm, T)	Wavelength (nm)	$t_{1/2}^{d}$ (s)	$t_{1/2}^{d}$ (min)	$t_{1/2}^{d}$ (h)	t_{PSS} (s)	$s_{1/2}$ (cycles)
1	BPPyr	Ar, 20°C	365	13990	233.16	3.89	10	1399
2	$[BPPyr\text{-}H_2]^{2+}$	Ar, 20°C	365	7128	118.81	1.98	10	713
3	$[BPPyr\text{-}Me_2]^{2+}$	Ar, 20°C	365	6037	100.62	1.68	5	1207
4	BPPyr	Air, 50°C	365	605	10.09	0.17	10	61
5	$[BPPyr\text{-}H_2]^{2+}$	Air, 50°C	365	361	6.02	0.10	10	36
6	$[BPPyr\text{-}Me_2]^{2+}$	Air, 50°C	365	245	4.09	0.07	5	49
7	$[BPPyr\text{-}Me_2]^{2+}$	Ar, 20°C	405	17359	289.31	4.82	5	3472
8	$[BPPyr\text{-}Me_2]^{2+}$	Air, 50°C	405	406	6.77	0.11	5	81

S2.1 Calculation of the thermal half-life times, the degradation half-life times, and the switching half-lives of the photoswitches

The kinetics of the thermal ring opening reactions were studied by monitoring the UV-vis absorbance changes at the long-wavelength maxima of the closed forms over time and at different temperatures. From these data and assuming first-order processes, the rate constant (k) was obtained from

$$ln \frac{A_{\mathrm{f}} - A}{A_{\mathrm{f}} - A_0} = -kt$$

where A_{f} is the final absorbance, A is the absorbance at a specific time t, and A_0 is the initial absorbance at the observed wavelength.

Thermal half-life times ($t_{½}$) of the closed isomeric forms of the switches were calculated using (the same equation was also used for determining degradation half-life times $t_{½}^{\mathrm{d}}$)

$$t_{½} = \frac{ln2}{k}$$

The number of switching cycles under which half of the molecules degrade irreversibly, which we denote switching half-life ($s_{½}$), was calculated as

$$s_{½} = \frac{t_{½}^{\mathrm{d}}}{t_{\mathrm{PSS}}}$$

where $t_{½}^{\mathrm{d}}$ is the degradation half-life time under continuous irradiation and t_{PSS} is the time required to reach the PSS.

S2.1.1 Kinetics studies of the thermal ring opening reaction

All kinetics studies were based on the Eyring equation (with all symbols having their usual meaning)

$$k = k_B \frac{T}{h} e^{-\frac{\Delta G_b{}^{‡}}{RT}}$$

where

$$\Delta G_b{}^{‡} = \Delta H^{‡} - T\Delta S^{‡}$$

and, consequently,

$$ln\frac{k}{T} = -\frac{\Delta H^{\ddagger}}{RT} + ln\frac{k_B}{h} + \frac{\Delta S^{\ddagger}}{R}$$

By monitoring the thermal ring opening at different temperatures and plotting $ln\frac{k}{T}$ against $\frac{1}{T}$, the slope $-\Delta H^{\ddagger}/R$ of the resulting straight line then provided the enthalpy of activation, whereas the intercept $ln\frac{k_B}{h} + \frac{\Delta S^{\ddagger}}{R}$ yielded the entropy of activation.

S2.1.2 Thermal ring opening of BPPyr-c to BPPyr-o at different temperatures in EtOH

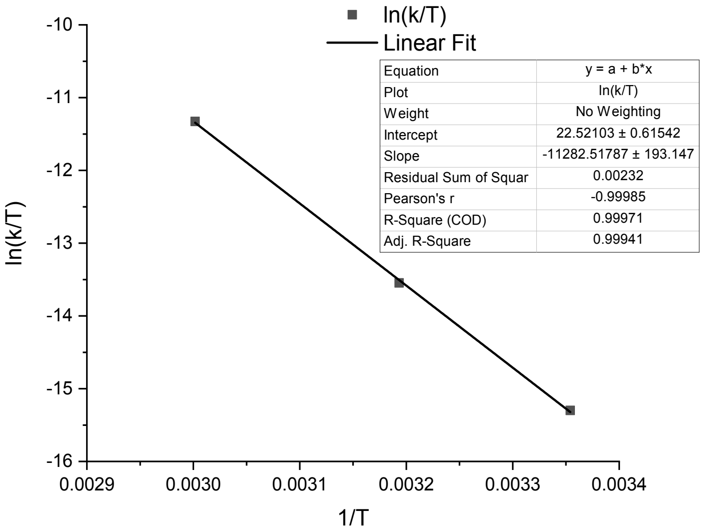

Figure S19. Eyring plot for the thermal ring opening of **BPPyr-c**.

Table S5. Kinetic data of the thermal ring opening of **BPPyr-c** in EtOH.

T (ºC)	T (K)	k (s^{-1})	$ln(k/T)$	$1/T$
25	298.15	6.76×10^{-5}	-15.29934	0.003354
40	313.15	4.09×10^{-4}	-13.54738	0.003193
60	333.15	4.01×10^{-3}	-11.32724	0.003002

$\Delta H^{\ddagger}$ = 93.8 kJ mol^{-1}
$\Delta S^{\ddagger}$ = –10.3 $Jmol^{-1}K^{-1}$
$t_{½}^{0°C}$ = 364998 s = 4.22 days

S2.1.3 Thermal ring opening of [BPPyr-H_2]$_2^+$-c to [BPPyr-H_2]$^{2+}$-o at different temperatures in EtOH

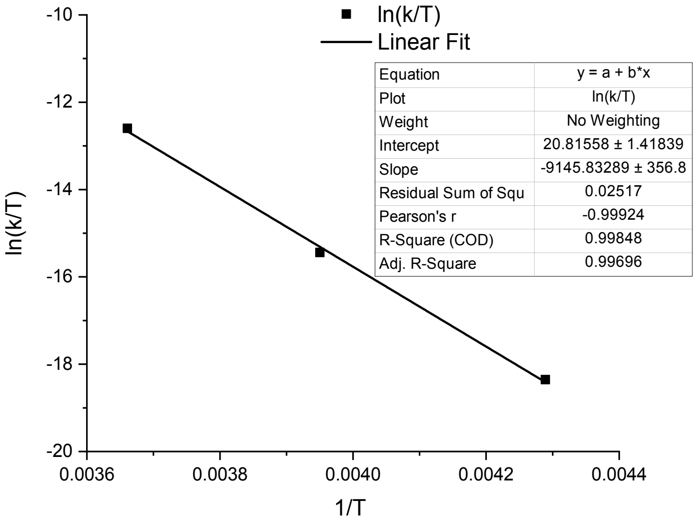

Figure S20. Eyring plot for the thermal ring opening of **[BPPyr-H^2]$^{2+}$-c**.

Table S6. Kinetic data of the thermal ring opening of **[BPPyr-H_2]$^{2+}$-c** in EtOH.

T **(ºC)**	***T*** **(K)**	***k*** **(s^{-1})**	***ln*(*k*/*T*)**	**1/*T***
0	273.15	9.23×10^{-4}	-12.597435	0.003661
-20	253.15	4.98×10^{-5}	-15.441933	0.00395
-40	233.15	2.50×10^{-6}	-18.352083	0.004289

$\Delta H^\ddagger$ = 76.0 kJ mol^{-1}
$\Delta S^\ddagger$ = –24.5 Jmol^{-1}K^{-1}
$t_{½}^{60°C}$ = 1.6 s

S2.1.4 Ring opening kinetics of $[BPPyr-H_2]^{2+}$-c to $[BPPyr-H_2]^{2+}$-o at different H^+ equivalents in EtOH at 25°C

Table S7. Change in the activation free energy of the thermal ring opening of **$[BPPyr-H_2]^{2+}$-c** in EtOH at 25°C compared to the neutral (0 eq H^+) form.

[H+] (eq)	k (s^{-1})	$ln(k_n/k_0)$	$\Delta\Delta G^{\ddagger}{}_{b}{}^{a}$
0	1.04×10^{-4}	-	-
1	7.23×10^{-4}	1.944	-4.8×10^{3}
2	6.94×10^{-3}	4.205	-1.0×10^{4}
3	9.19×10^{-3}	4.486	-1.1×10^{4}
10	3.92×10^{-2}	5.938	-1.5×10^{4}

^{a} $\Delta\Delta G_b{}^{\ddagger} = -RT\ln(k_n/k_0)$, where k_n is the reaction rate in the presence of a specific H^+ equivalent, k_0 is the reaction rate in the absence of H^+.

S2.2 Quantum yield determination for the light-induced ring closing and ring opening reactions of BPPyr

Quantum yields were determined according to the reports by Stranius and Börjesson[6] and Drapała and co-workers.[7] Samples were irradiated using a Prizmatix multi-wavelength fiber-coupled LED light source equipped with a 365 nm diode, providing a photon flux of 1.40×10^{16} s^{-1}, and a Chanzon 660 nm LED providing a photon flux of 2.33×10^{18} s^{-1}.

An l = 1 cm cuvette was loaded with a concentrated sample solution (1.70×10^{-3} M) in ethanol-d_6 and placed in the light beam. Due to the high concentration, transmittance was assumed to be zero and was excluded from the calculations.[6] The ring closing reaction was monitored only at the early, linear stage of the transformation. In the case of the ring opening reaction, samples (40 μL) were taken from the concentrated solution at 2 s irradiation intervals and diluted to 2 mL with ethanol (to a final concentration of 3.41×10^{-5} M) for UV-vis spectrum acquisition. The distribution of isomers was determined via the ^{1}H NMR spectrum of a sample taken directly from the cuvette after prolonged irradiation to reach a closed isomer rich state. The molar absorbance of the closed form was calculated according to the following equation:

$$A_c^{\lambda} = \varepsilon_c^{\lambda} \cdot c_0 \cdot l \cdot P(t) \qquad (1)$$

where A_c^λ and ε_c^λ are the absorbance and molar absorbance of the closed form at a specified wavelength, respectively, c_0 is the initial concentration of the solution, l is the optical path length, and $P(t)$ is the conversion at a given time.

In our case, at 680 nm the closed form was the solely absorbing isomer, therefore, the changes in absorbance were monitored at this wavelength. ^{1}H NMR and UV-vis analysis of the same sample gave $\varepsilon_c^{680\,nm}$ = 17699 $M^{-1}cm^{-1}$. The concentration of the closed form was calculated for each point of measurement and plotted as the function of irradiation time.

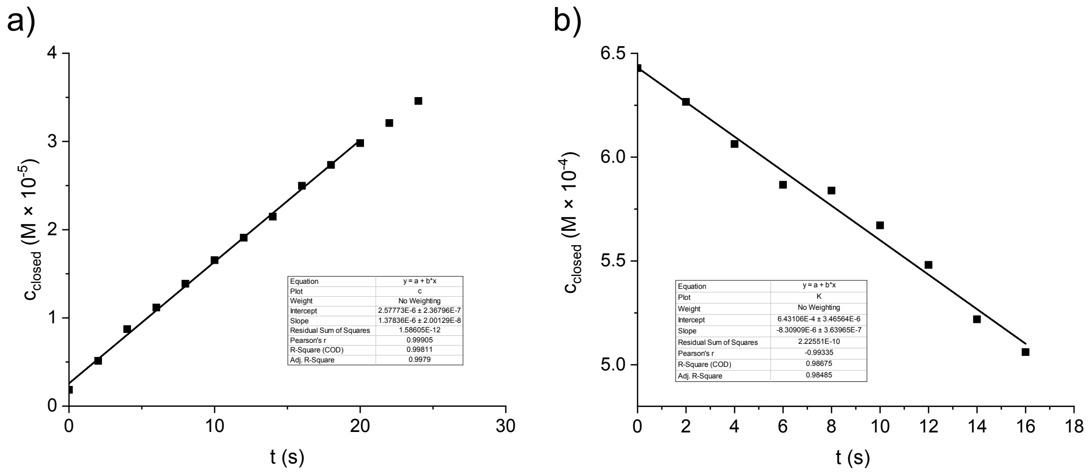

Fig. S21. Concentration changes of the closed isomer in the function of irradiation time in the ring-closing (a) and the ring-opening (b) reactions.

Photochemical quantum yields (*Φ*) were calculated according to the following equation:

$$\Phi = \frac{{}^{dc}/_{dt} \cdot V \cdot N_A}{I_{abs}} \qquad (2)$$

where *dc/dt* is the slope of the linear fit to the initial region of the c_{closed} vs. *t* plot, *V* is the volume of the irradiated sample, N_A is Avogadro's number, I_{abs} is the intensity of absorbed photons.

The obtained quantum yields are Φ_{RC} = 0.12 and Φ_{RO} = 0.0043, respectively, for the ring opening and ring closing reactions.

S3 Synthetic Procedures

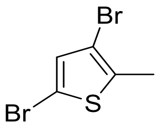

3,5-Dibromo-2-methylthiophene (2). 2-Methylthiophene (5.00 g, 50.94 mmol) was dissolved in 50 mL glacial acetic acid and N-bromosuccinimide (19.04 g, 107.0 mmol) was added slowly at rt and the reaction mixture was stirred for 18 h. Half of the solvent was removed by rotary evaporation and the remaining acidic solution was poured into water (150 mL). The crude product was extracted with DCM (2×30 mL) and the organic phase was washed with aqueous $NaHCO_3$ (1M, 2×50 mL). The solvent was removed by rotary evaporation, and the residue was purified by flash column chromatography on silica gel using gradient elution (hexane to hexane/ethyl acetate 5%) to obtain **2** as a light-yellow oil (11.09 g, 85%). ^{1}H NMR (500 MHz, $CDCl_3$) δ = 6.86 (s, 1H), 2.34 (s, 3H) ppm. ^{13}C NMR (75 MHz, $CDCl_3$) δ = 136.15, 132.07, 108.81, 108.61, 14.97 ppm.

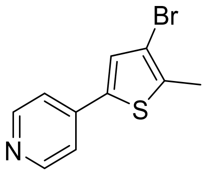

4-(4-Bromo-5-methylthiophen-2-yl)pyridine (3). Tetrakis(triphenylphosphine)palladium ($Pd(PPh_3)_4$) (12.4 mg, 10.7 µmol) and aqueous Na_2CO_3 (207 mg, 1.95 mmol in 2 mL water) was added to a stirred solution of 3,5-dibromo-2-methylthiophene **2** (500 mg, 1.95 mmol) and pyridin-4-ylboronic acid hydrate (275 mg, 1.95 mmol) in 5 mL THF at rt, and the reaction mixture was stirred at reflux temperature (75 °C) for 18 h under argon atmosphere. The cooled solution was poured onto water (20 mL) and extracted with ethyl acetate (2×20 mL). The combined organic layer was dried over $MgSO_4$, and the solvent was removed by rotary evaporation. The residue was purified by flash column chromatography on silica gel using gradient elution (hexane to hexane/ethyl acetate 50%) to obtain **3** as a yellow solid (350 mg, 71%). ^{1}H NMR (500 MHz, $CDCl_3$) δ = 8.57 (dd, *J* = 4.6, 1.5 Hz, 2H), 7.35 (dd, *J* = 4.6, 1.6 Hz, 2H), 7.29 (s, 1H), 2.43 (s, 3H) ppm. ^{13}C NMR (126 MHz, $CDCl_3$) δ = 150.61, 140.50, 137.98, 136.56, 128.04, 119.29, 110.76, 15.18 ppm.

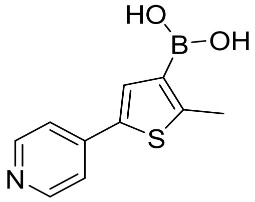

(2-methyl-5-(pyridin-4-yl)thiophen-3-yl)boronic acid (4). To a stirred solution of **3** (350 mg, 1.377 mmol) in 10 mL dry THF, *n*BuLi (1.033 mL, 1.653 mmol, 1.6M in hexane) was

added slowly at -78 °C under argon atmosphere. After 15 min reaction time trimethylborate (230 µL, 2.066 mmol) was added dropwise and the mixture was allowed to warm to rt and the reaction was stirred for an additional 16 h. After completion most of the THF was removed by rotary evaporation, water (100 mL) and NaOH solution (1 M, 100 mL) were added, and the resulting aqueous phase was washed with DCM (2 × 20 mL). The aqueous phase was neutralized with ccHCl. The resulting thick suspension was extracted with EtOAc (2 × 100 mL) and the combined organic phase was dried on $MgSO_4$, filtered, and the solvent was removed by rotary evaporation. The resulting product was used in the next step without further purification (246 mg, 82%). ^1H NMR (500 MHz, CD_3OD) δ = 8.44 (dd, *J* = 4.8, 1.5 Hz, 2H), 7.64 (s, 1H), 7.57 (dd, *J* = 4.9, 1.4 Hz, 2H), 2.57 (s, 3H) ppm. ^{13}C NMR (126 MHz, CD_3OD) δ = 150.32, 144.20, 132.14, 132.06, 121.01, 120.13, 120.09, 16.20 ppm.

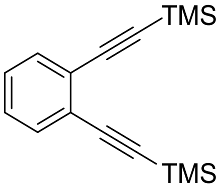

1,2-bis((trimethylsilyl)ethynyl)benzene (6). 1,2-Diiodobenzene (15.0 g, 45.467 mmol), trimethylsilyl acetylene (14.5 mL, 104.6 mmol, 2.3 eq), CuI (43 mg, 277 µmol, 0.5 mol%), and $PdCl_2(PPh_3)_2$ (319 mg, 455 µmol, 1 mol%) were added to TEA (100 mL) under argon atmosphere. The reaction mixture was stirred at 65°C for 5 h. After completion Et_2O (100 mL) was added and the resulting suspension was filtered through a pad of Celite. The solvents were removed by rotary evaporation, water (150 mL) was added, and the aqueous mixture was extracted with Et_2O (2 × 50 mL). The combined organic phase was washed with brine (20 mL) and dried over $MgSO_4$. After filtration the solvent was removed by rotary evaporation and the crude product was purified by flash column chromatography on silica gel using hexane as an eluent to obtain **6** as a yellow oil (9.3 g, 76%). ^1H NMR (500 MHz, $CDCl_3$) δ = 7.45 (dd, *J* = 5.8, 3.4 Hz, 2H), 7.23 (dd, *J* = 5.8, 3.3 Hz, 2H), 0.27 (s, 18H) ppm. ^{13}C NMR (126 MHz, $CDCl_3$) δ = 132.45, 128.17, 125.97, 103.43, 98.60, 0.21 ppm.

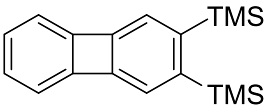

2,3-Bis(trimethylsilyl)biphenylene (8). To a solution of **6** (6.300 g, 23.29 mmol) in a mixture of THF (40 mL) and MeOH (40 mL) under argon atmosphere K_2CO_3 (4.828 g, 34.93 mmol, 1.5 eq) was added at 0°C, and the reaction was stirred for 2 h. After completion DCM (50 mL) was added, and the resulting suspension was filtered through a pad of Celite. The solvents were removed by rotary evaporation at 20°C and the residue was purified by flash column chromatography on silica gel using pentane as an eluent to obtain 1,2-bis(ethynyl)arene (**7**), which was immediately transferred into a syringe containing cyclopentadienylcobalt dicarbonyl ($CpCo(CO)_2$) (215 µL, 1.609 µmol, 7 mol%) in a mixture of 1,2-bis(trimethylsilyl)ethyne (BTMSA) (10 mL) and o-xylene (5 mL). The mixture was

added slowly to refluxing BTMSA (25 mL) under argon atmosphere over a period of 8 h using a syringe pump, and stirred for an additional 8 h at 140°C. The resulting solution was allowed to cool to rt and the excess BTMSA was removed under vacuum. The crude product was purified by flash column chromatography on silica gel using hexane as an eluent to obtain **8** as a red oil (1.588 g, 23%). ^{1}H NMR (500 MHz, $CDCl_3$) δ = 6.97 (s, 2H), 6.73 (dd, J = 4.8, 2.9 Hz, 2H), 6.67 (dd, J = 4.9, 2.8 Hz, 2H), 0.34 (s, 18H) ppm. ^{13}C NMR (126 MHz, $CDCl_3$) δ = 152.71, 150.65, 147.98, 128.29, 122.87, 117.81, 2.30 ppm.

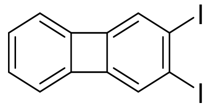

2,3-diiodobiphenylene (9). To a solution of **8** (1.00 g, 3.37 mmol) in glacial acetic acid (25 mL) N-iodosuccinimide (1.59 g, 7.08 mmol, 2.1 eq) was added in small portions and stirred at rt for 2 h. After completion most of the acetic acid was removed by rotary evaporation, DCM (50 mL) was added, and the resulting suspension was washed with $NaHSO_3$ solution (1M, 20 mL). The organic layer was dried over $MgSO_4$, and the solvent was removed by rotary evaporation. The crude product was purified by flash column chromatography on silica gel using hexane as an eluent to obtain **9** as a yellow solid (1.014 g, 74%). ^{1}H NMR (500 MHz, $CDCl_3$) δ = 7.15 (s, 2H), 6.81 (dd, J = 4.7, 2.9 Hz, 2H), 6.69 (dd, J = 4.7, 2.9 Hz, 2H) ppm. ^{13}C NMR (126 MHz, $CDCl_3$) δ = 151.79, 149.92, 129.27, 127.74, 118.96, 106.39 ppm.

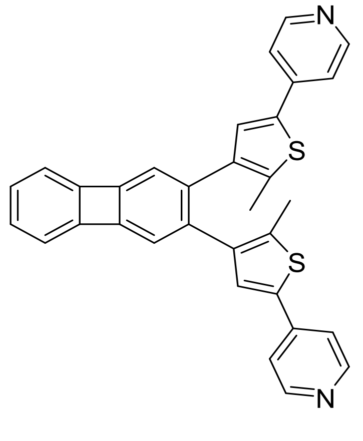

2,3-bis(2-methyl-5-(pyridin-4-yl)thiophen-3-yl)biphenylene (BPPyr). [1,1′-Bis(diphenylphosphino)ferrocene] dichloropalladium(II) ($Pd(dppf)Cl_2$) (37 mg, 46.4 µmol, 7.5 mol%) was added to a mixture of **8** (250 mg, 619 µmol) and **4** (339 mg, 1.547 mmol, 2.5 eq.) in THF (10 mL) and Na_2CO_3 (394 mg, 3.713 mmol, 6 eq.) in water (2.5 mL) and stirred at 75°C for 20 h under argon atmosphere. After completion, the reaction mixture was diluted with EtOAc (25 mL) and filtered through a pad of Celite. The solvents were removed by rotary evaporation and the residue was pre-purified by flash column chromatography on silica gel using gradient elution (from hexane/ethyl acetate = 9:1 to hexane/ethyl acetate = 1:1). The crude product was further purified using gradient elution flash column chromatography on reversed phase silica gel (from H_2O/MeCN = 95:5 to MeCN, in the presence of 0.1% TFA). After chromatography, most of the MeCN was removed by rotary evaporation, the pH was

adjusted to ~ 8 using aqueous Na_2CO_3 solution (1M) and the product was extracted with DCM (2 × 50 mL). The combined organic layer was dried over $MgSO_4$, and the solvent was removed by rotary evaporation, yielding **BPPyr** as a green solid (84 mg, 27%). ^{1}H NMR (500 MHz, CD_3CN) δ = 8.45 (dd, J = 4.5, 1.7 Hz, 4H), 7.34 (dd, J = 4.5, 1.7 Hz, 4H), 7.18 (s, 2H), 6.87 (dd, J = 4.9, 2.9 Hz, 2H), 6.79 (dd, J = 4.9, 2.9 Hz, 2H), 6.77 (s, 2H), 2.25 (s, 6H) ppm. ^{13}C NMR (126 MHz, CD_2Cl_2) δ = 151.18, 151.00, 150.85, 141.67, 140.27, 138.55, 136.99, 135.77, 129.36, 128.42, 120.14, 119.79, 118.50, 14.66 ppm. HRMS (ESI) m/z: $[M-H]^-$ calcd for $C_{32}H_{23}N_2S_2^-$: 499.1302; found: 499.1303.

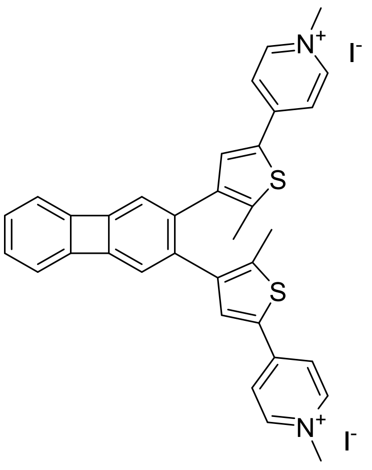

4,4'-(Biphenylene-2,3-diylbis(5-methylthiophene-4,2-diyl))bis(1-methylpyridinium) iodide ($[BPPyrMe_2]^{2+}$). To a solution of **BPPyr** (20 mg, 40 μmol) in dry MeCN (5 mL), iodomethane (40 μL, 640 μmol, 16 eq.) was added and the reaction mixture was stirred at 80°C for 18 h under argon atmosphere. After completion, all volatiles were removed by rotary evaporation yielding the clean **$[BPPyrMe_2]^{2+}$** as a yellow solid (30 mg, 96%). ^{1}H NMR (500 MHz, CD_3CN) δ = 8.49 (d, J = 6.9 Hz, 4H), 7.94 (d, J = 7.0 Hz, 4H), 7.68 (s, 2H), 6.90 (dd, J = 4.9, 2.9 Hz, 2H), 6.82 (dd, J = 4.9, 2.9 Hz, 2H), 6.76 (s, 2H), 4.20 (s, 6H), 2.30 (s, 6H) ppm. ^{13}C NMR (126 MHz, CD_3CN) δ = 152.07, 151.19, 149.39, 146.60, 145.98, 142.15, 135.46, 135.42, 133.48, 130.24, 122.68, 120.46, 119.42, 48.21, 15.07 ppm. HRMS (ESI) m/z: $[M-H]^-$ calcd for $C_{34}H_{28}N_2S_2I^-$: 655.0738; found: 655.0734.

Preparation of the $[BPPyrMe_2]^{2+}$ embedded gelatin gel

Gelatin powder (10 g) was mixed with cold water (10 mL) in a beaker. After hydration, hot water (90°C, 50 mL) was added to the slurry and stirred until complete dissolution. The formed foam was removed from the top of the solution. In a petri dish, 25 mL of warm gelatin solution was mixed with a solution of **$[BPPyrMe_2]^{2+}$** (10 mg) in DMSO (1 mL). The mixture was placed in the fridge and allowed to solidify overnight.

S4 NMR Spectra

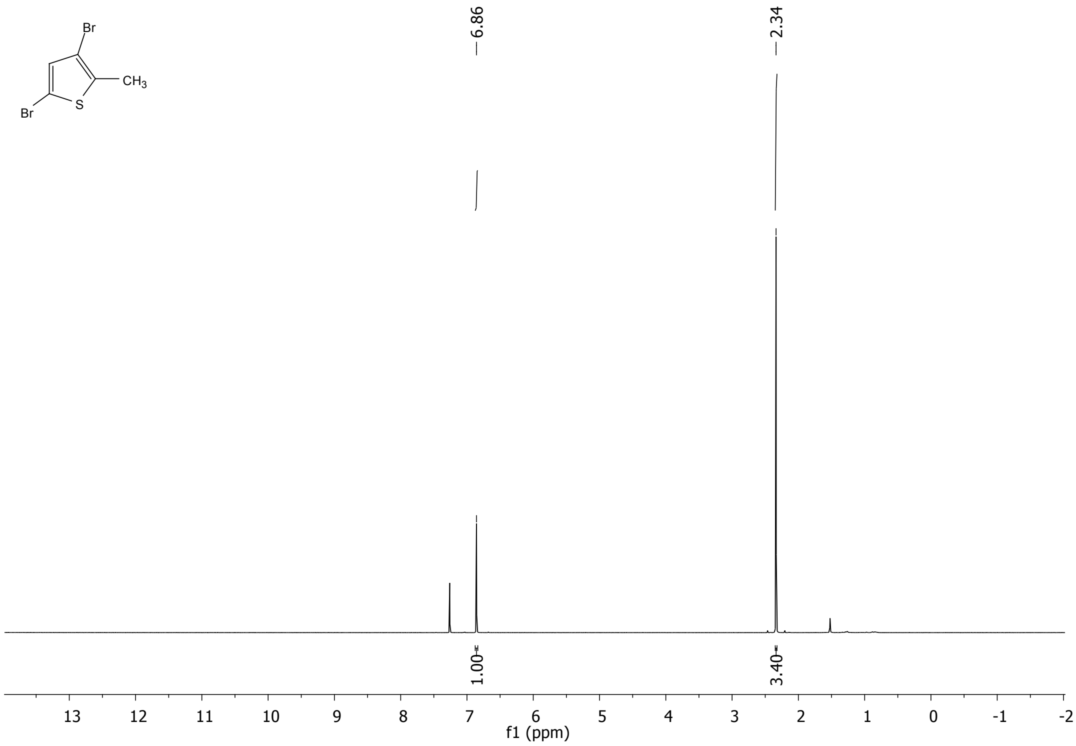

Figure S22. ^{1}H-NMR spectrum of **2** in $CDCl_3$ (500 MHz).

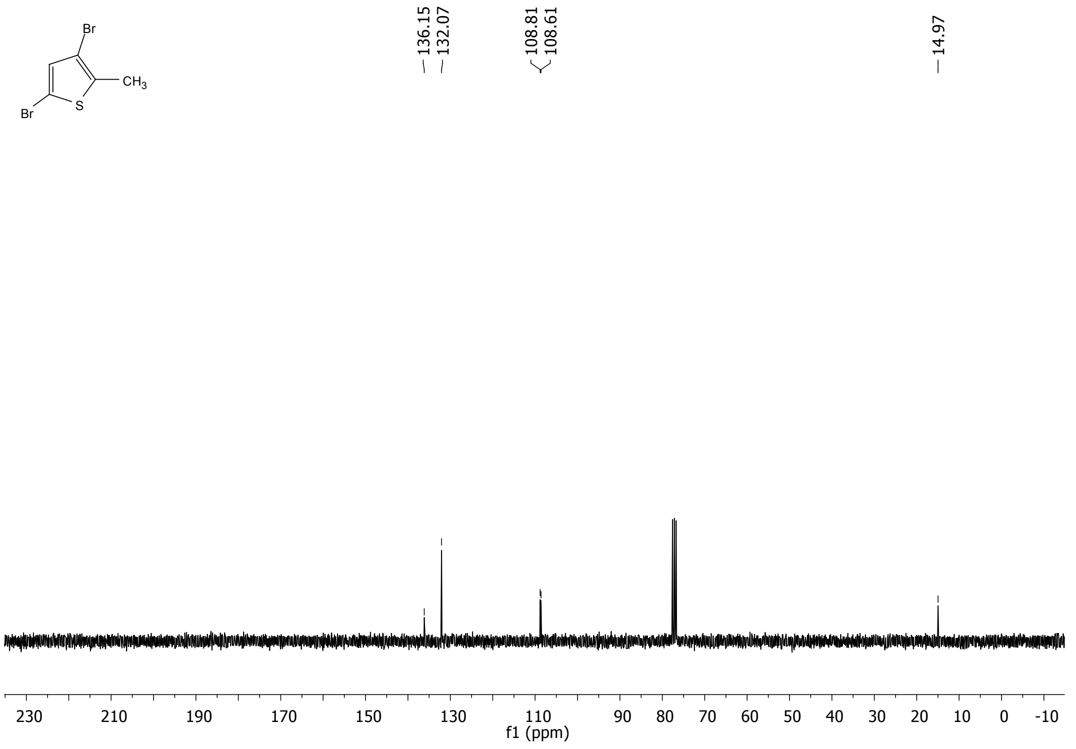

Figure S23. $^{13}C\{^{1}H\}$-NMR spectrum of **2** in $CDCl_3$ (126 MHz).

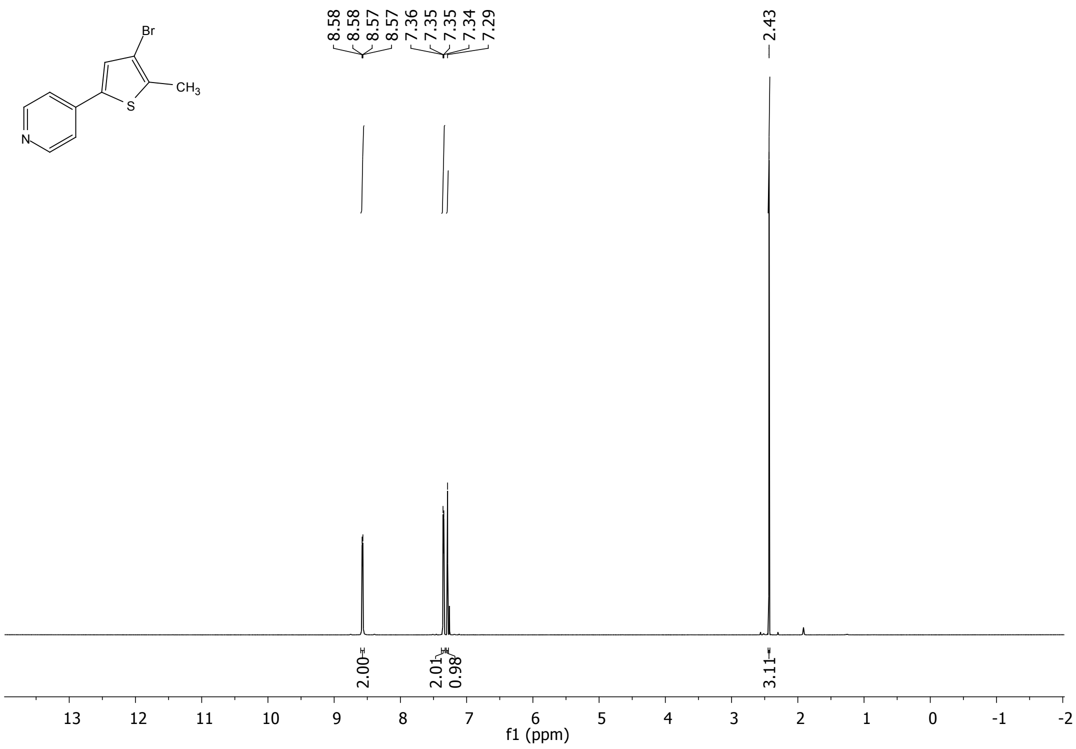

Figure S24. ^{1}H-NMR spectrum of **3** in $CDCl_3$ (500 MHz).

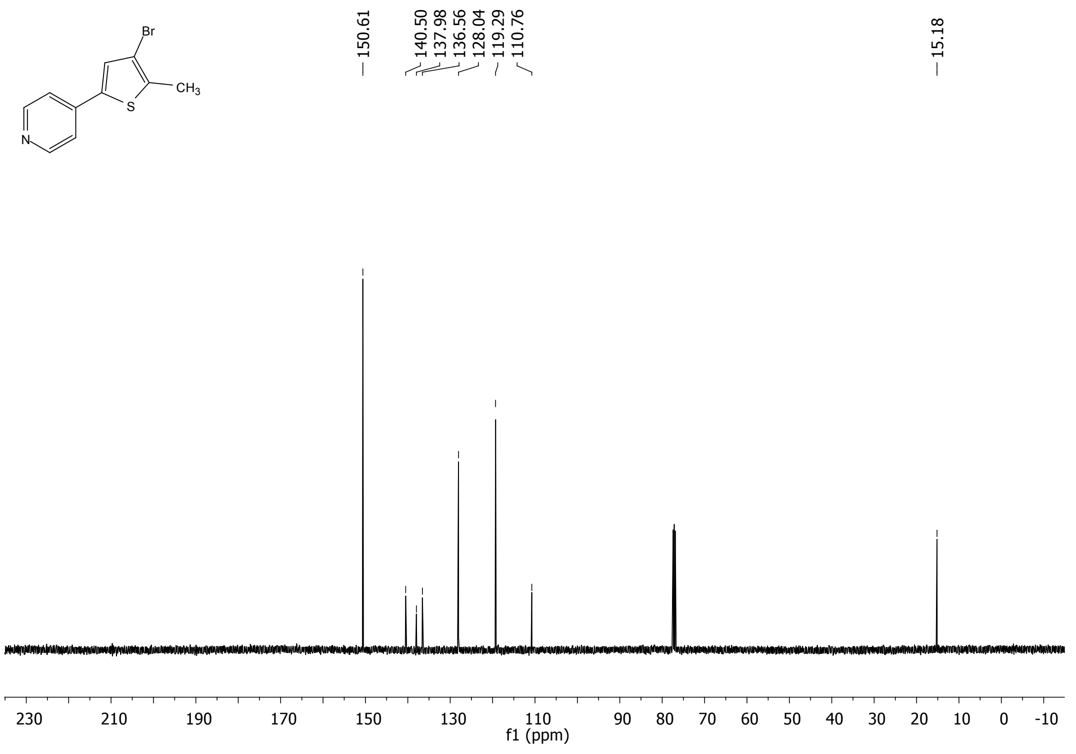

Figure S25. $^{13}C\{^1H\}$-NMR spectrum of **3** in $CDCl_3$ (126 MHz).

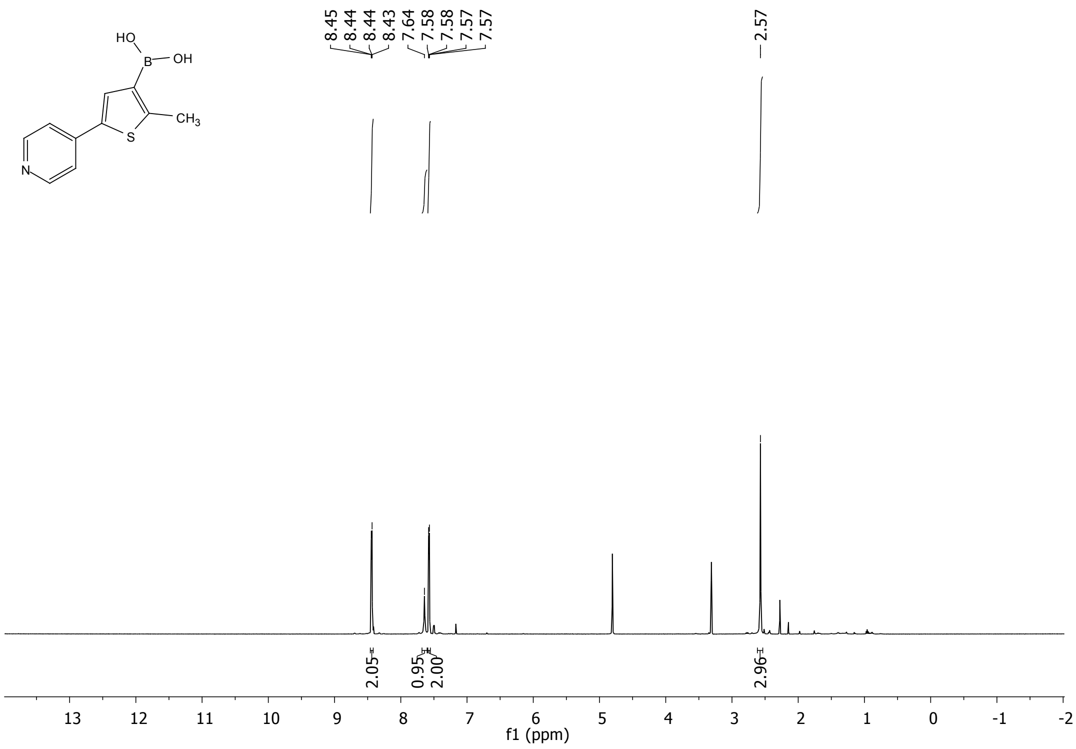

Figure S26. ^{1}H-NMR spectrum of **4** in CD_3OD (500 MHz).

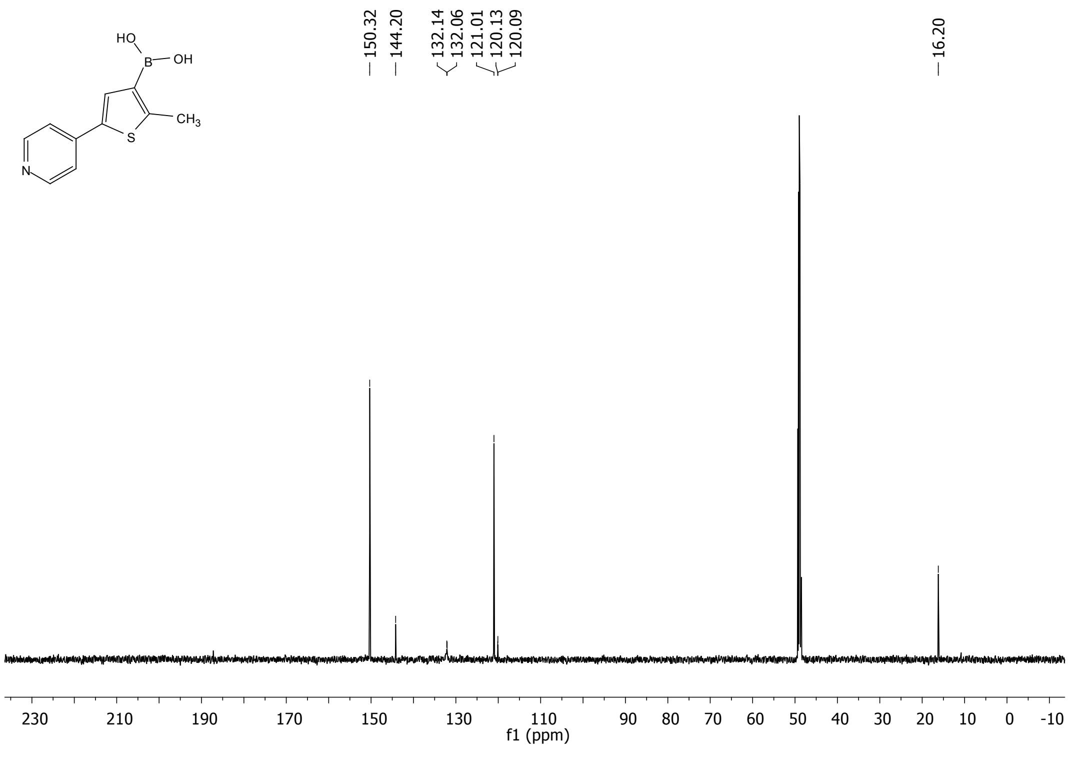

Figure S27. $^{13}C\{^{1}H\}$-NMR spectrum of **5** in CD_3OD (126 MHz).

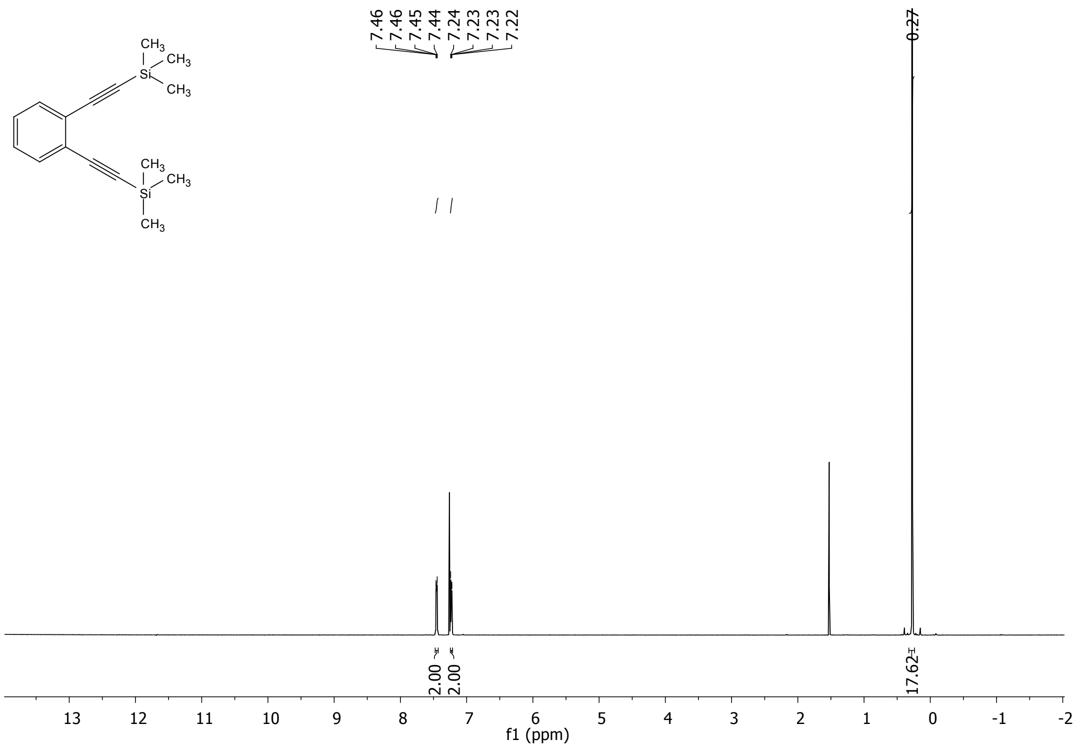

Figure S28. ^{1}H-NMR spectrum of **6** in $CDCl_3$ (500 MHz).

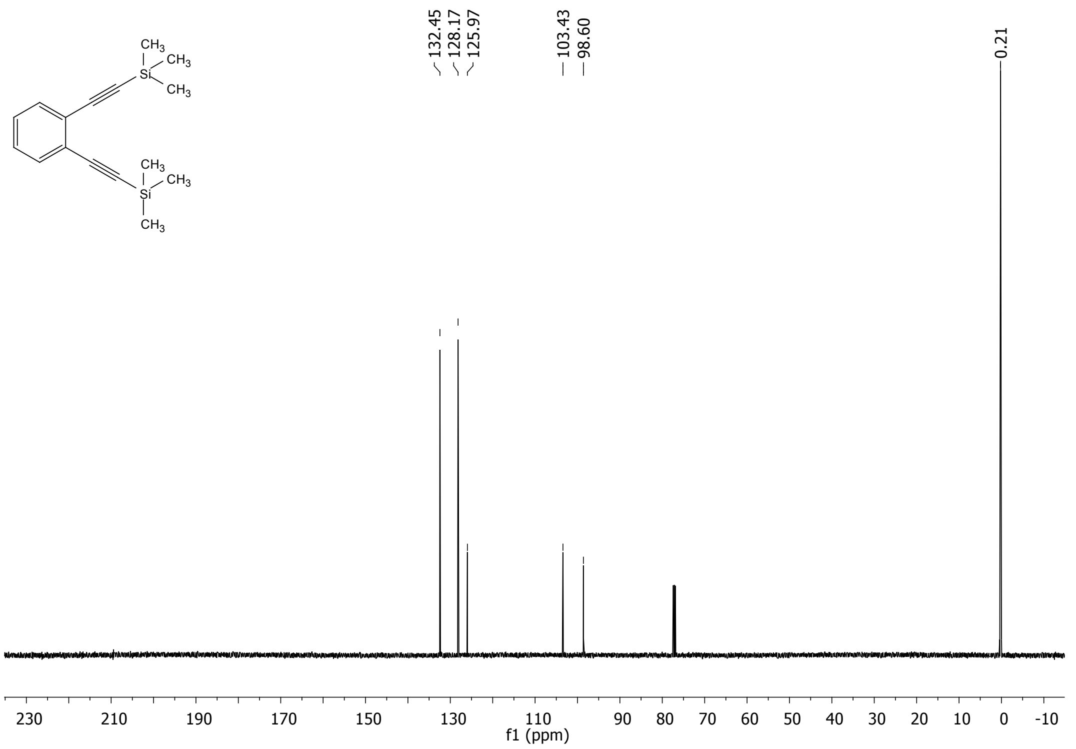

Figure S29. $^{13}C\{^1H\}$-NMR spectrum of **6** in $CDCl_3$ (126 MHz).

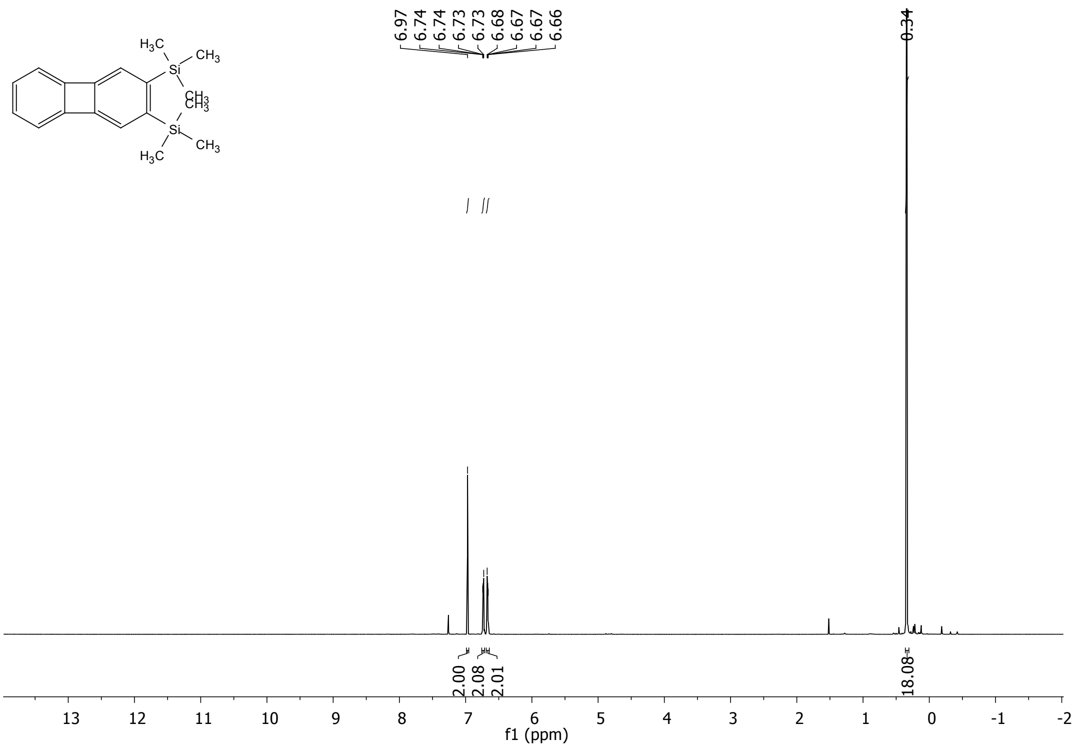

Figure S30. ^{1}H-NMR spectrum of **8** in $CDCl_3$ (500 MHz).

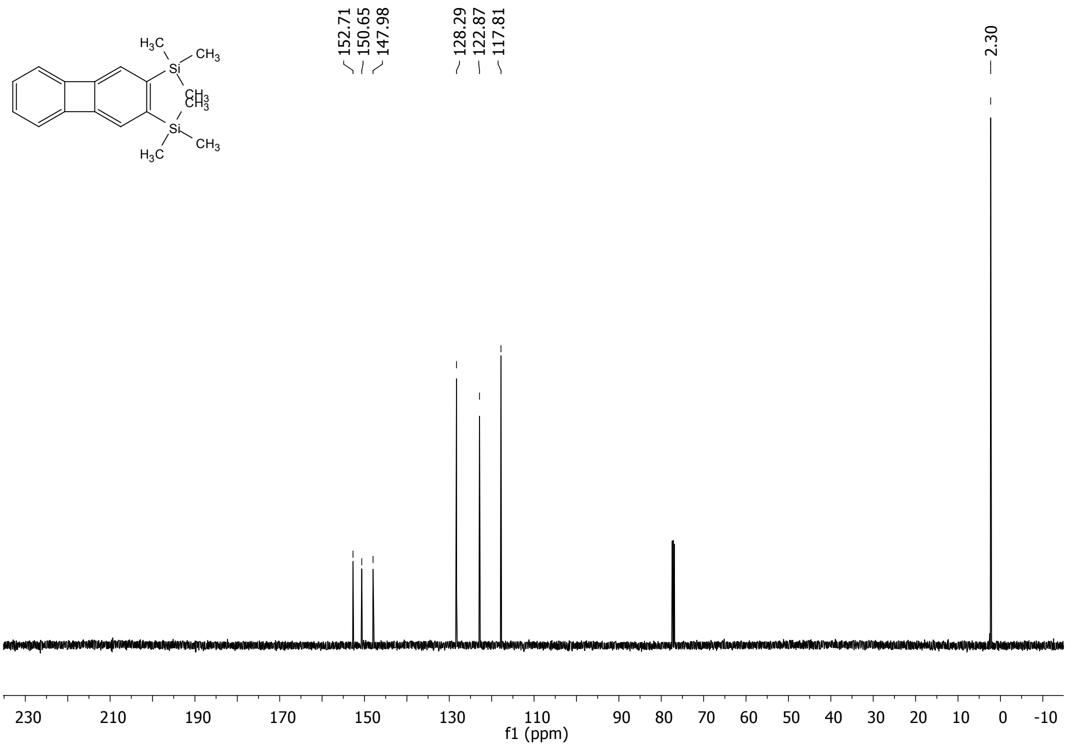

Figure S31. $^{13}C\{^{1}H\}$-NMR spectrum of **8** in $CDCl_3$ (126 MHz).

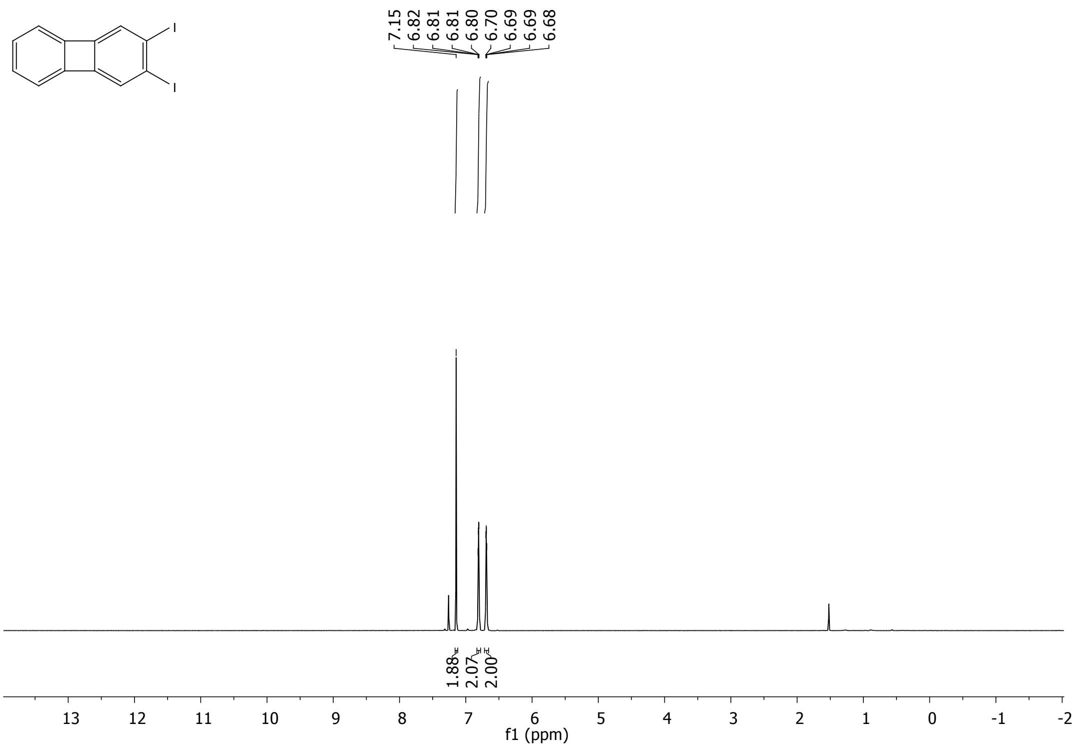

Figure S32. ^{1}H-NMR spectrum of **9** in $CDCl_3$ (500 MHz).

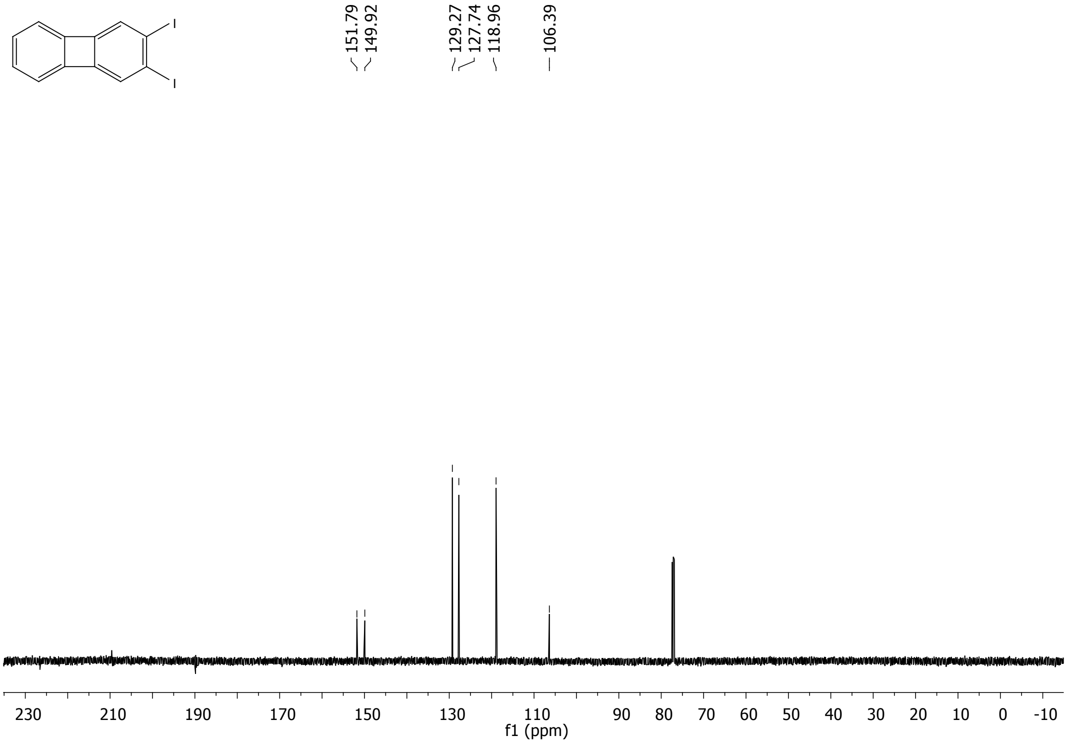

Figure S33. $^{13}C\{^{1}H\}$-NMR spectrum of **9** in $CDCl_3$ (126 MHz).

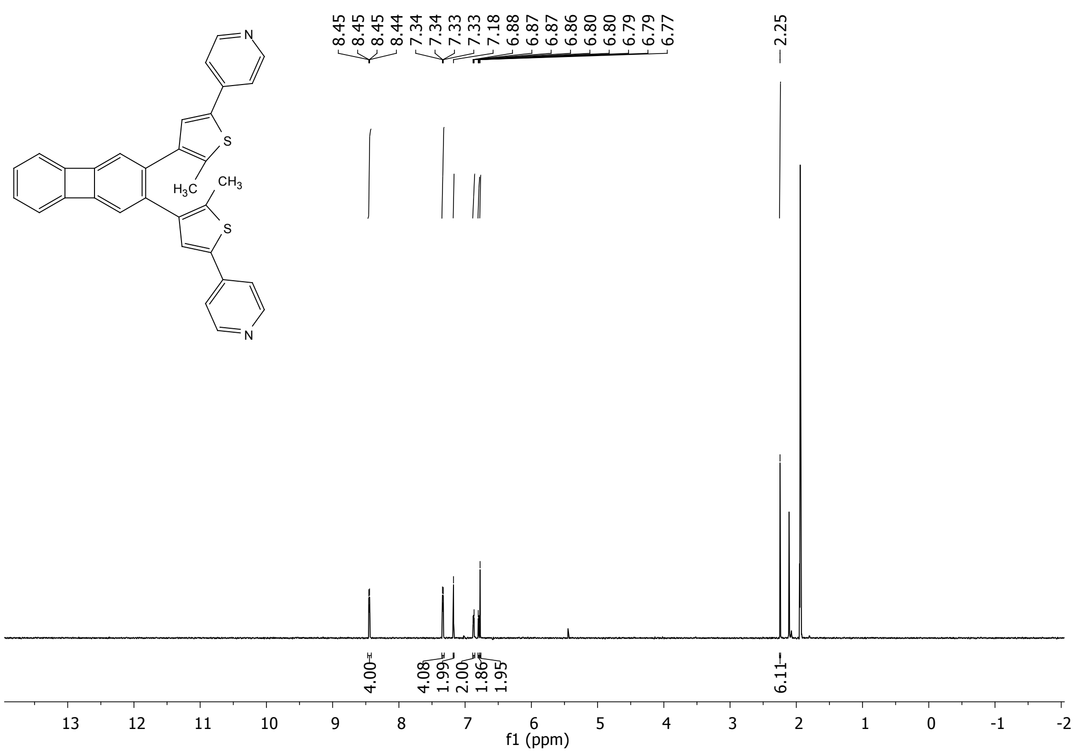

Figure S34. ^{1}H-NMR spectrum of **BPPyr** in CD_3CN (500 MHz).

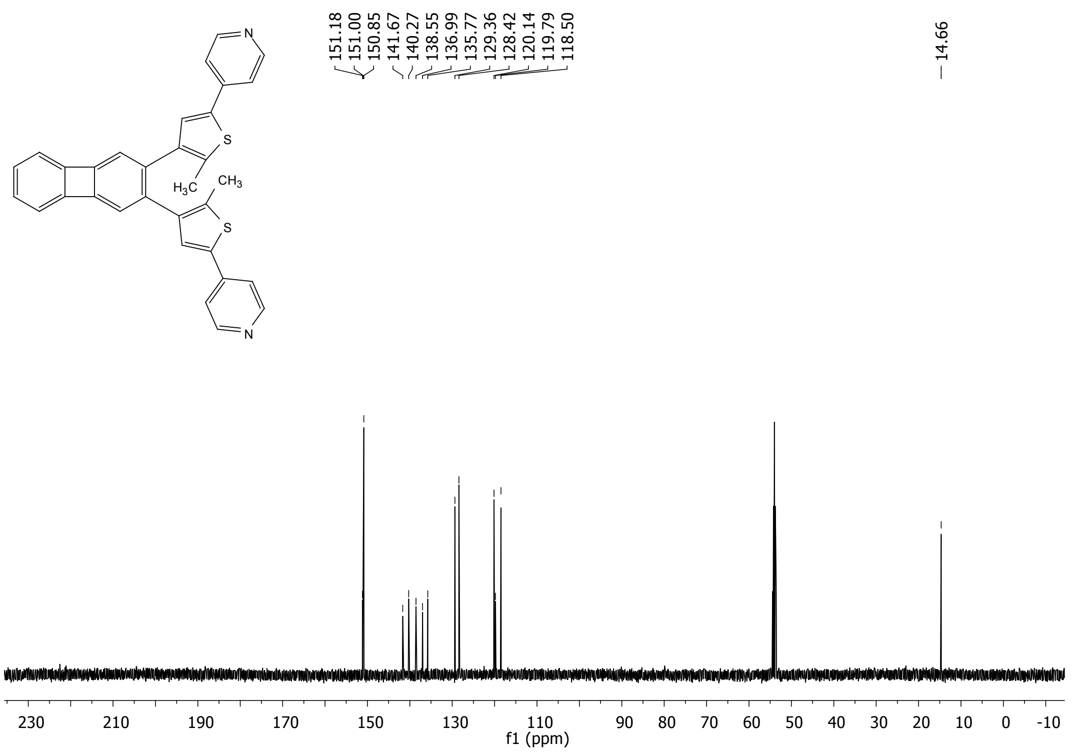

Figure S35. $^{13}C\{^{1}H\}$-NMR spectrum of **BPPyr** in CD_3CN (126 MHz).

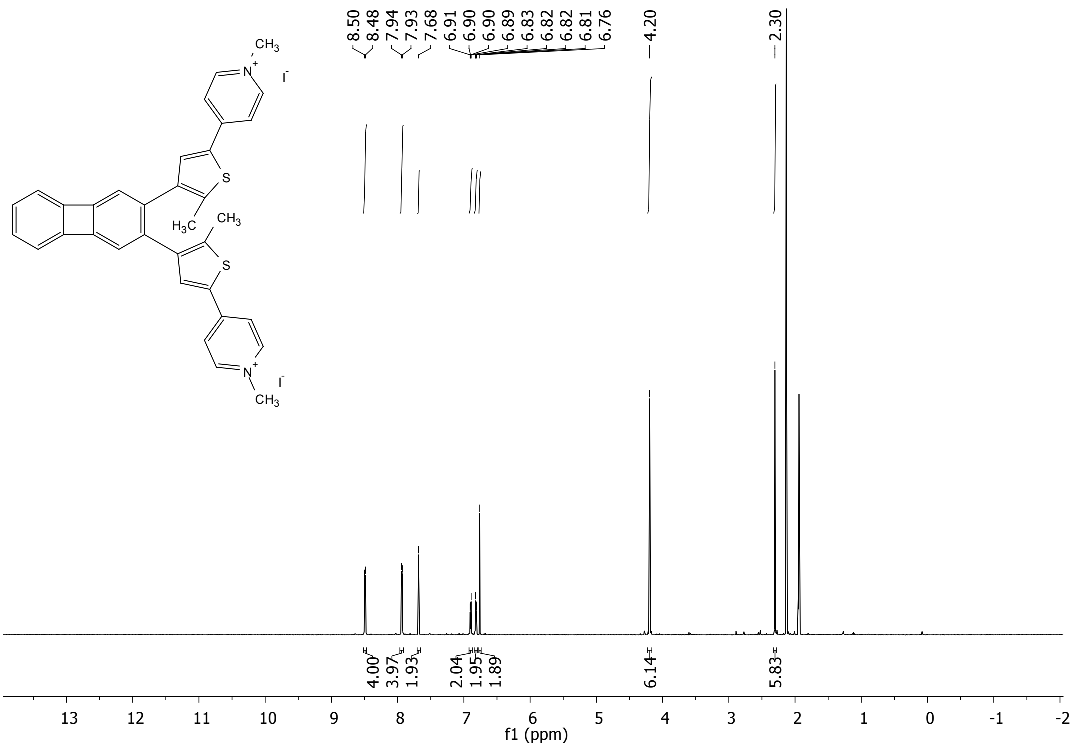

Figure S36. ^{1}H-NMR spectrum of **[BPPyr-Me$_2$]$^{2+}$** in CD_3CN (500 MHz).

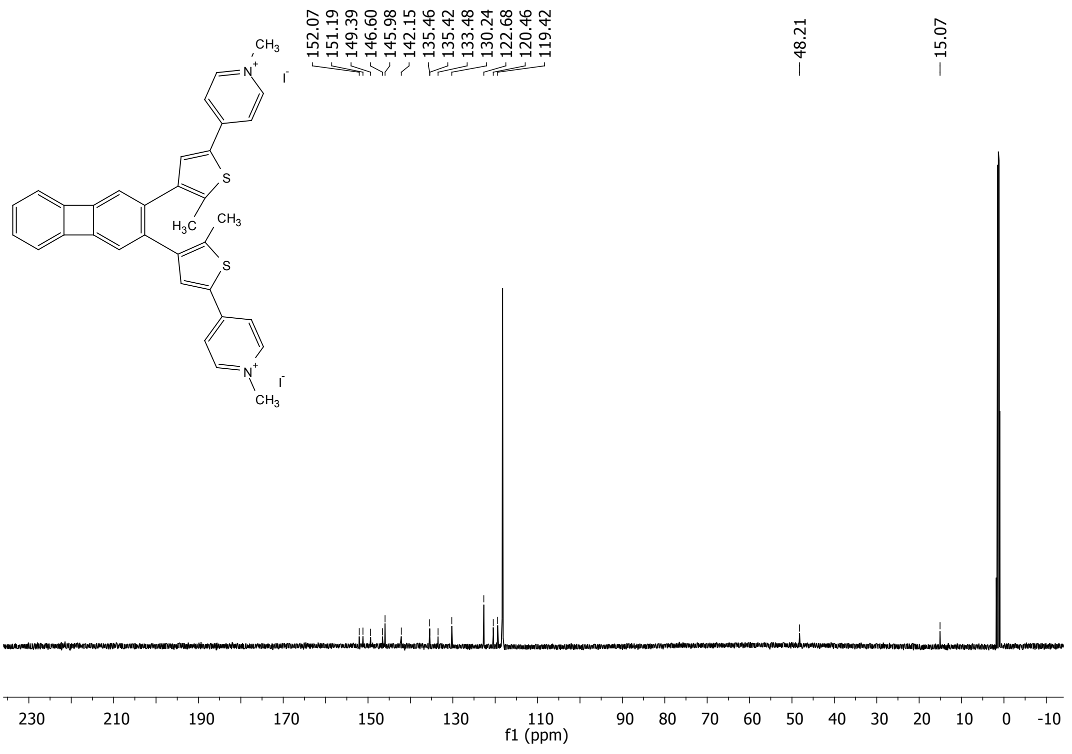

Figure S37. $^{13}C\{^{1}H\}$-NMR spectrum of **[BPPyr-Me$_2$]$^{2+}$** in CD_3CN (126 MHz).

S5 References

(1) Zhao, Y.; Truhlar, D. G. The M06 Suite of Density Functionals for Main Group Thermochemistry, Thermochemical Kinetics, Noncovalent Interactions, Excited States, and Transition Elements: Two New Functionals and Systematic Testing of Four M06-Class Functionals and 12 other Functionals. *Theor. Chem. Acc.* **2008**, *120*, 215–241.

(2) Chai, J.-D.; Head-Gordon, M. Long-Range Corrected Hybrid Density Functionals with Damped Atom-Atom Dispersion Corrections. *Phys. Chem. Chem. Phys.* **2008**, *10*, 6615−6620.

(3) Tomasi, J.; Mennucci, B.; Cammi, R. Quantum Mechanical Continuum Solvation Models. *Chem. Rev.* **2005**, *105*, 2999–3093.

(4) Frisch, M. J.; Trucks, G. W.; Schlegel, H. B.; Scuseria, G. E.; Robb, M. A.; Cheeseman, J. R.; Scalmani, G.; Barone, V.; Petersson, G. A.; Nakatsuji, H.; Li, X.; Caricato, M.; Marenich, A. V.; Bloino, J.; Janesko, B. G.; Gomperts, R.; Mennucci, B.; Hratchian, H. P.; Ortiz, J. V.; Izmaylov, A. F.; Sonnenberg, J. L.; Williams-Young, D.; Ding, F.; Lipparini, F.; Egidi, F.; Goings, J.; Peng, B.; Petrone, A.; Henderson, T.; Ranasinghe, D.; Zakrzewski, V. G.; Gao, J.; Rega, N.; Zheng, G.; Liang, W.; Hada, M.; Ehara, M.; Toyota, K.; Fukuda, R.; Hasegawa, J.; Ishida, M.; Nakajima, T.; Honda, Y.; Kitao, O.; Nakai, H.; Vreven, T.; Throssell, K.; Montgomery, J. A., Jr.; Peralta, J. E.; Ogliaro, F.; Bearpark, M. J.; Heyd, J. J.; Brothers, E. N.; Kudin, K. N.; Staroverov, V. N.; Keith, T. A.; Kobayashi, R.; Normand, J.; Raghavachari, K.; Rendell, A. P.; Burant, J. C.; Iyengar, S. S.; Tomasi, J.; Cossi, M.; Millam, J. M.; Klene, M.; Adamo, C.; Cammi, R.; Ochterski, J. W.; Martin, R. L.; Morokuma, K.; Farkas, O.; Foresman, J. B.; Fox, D. J. *Gaussian 16, Revision C.01*; Gaussian, Inc.: Wallingford CT, 2016.

(5) Mardirossian, N.; Head-Gordon, M. Thirty Years of Density Functional Theory in Computational Chemistry: An Overview and Extensive Assessment of 200 Density Functionals. *Mol. Phys.* **2017**, *115*, 2315–2372.

(6) Stranius, K.; Börjesson, K.; Determining the Photoisomerization Quantum Yield of Photoswitchable Molecules in Solution and in the Solid State. *Sci. Rep.* **2017**, *7*, 41145.

(7) Drapała, J.; Durka, K.; Jarzembska, K. N.; Kamiński, R.; Reliable Determination of Photoreaction Kinetics and Cyclization/Cycloreversion Quantum Yields for Dithienylethene Switches. *Angew. Chem. Int. Ed.* **2026**, *65*, e202514591.

S6 Electronic Energies and Cartesian Coordinates of Calculated Structures

Below, the electronic energies (E, in Ha) and the Cartesian coordinates (in Å) of the open form (o), the closed form (c) and the transition structure (TS) for thermal ring opening are given for the different switches in Figure 13 of the main text, as calculated at the M06-2X/cc-pVTZ/PCM level of theory.

BPPyr-o

E = –2138.41887998 Ha (all vibrational frequencies real)

C	0.65150	1.16389	0.25159
C	-0.65170	1.16372	-0.25159
C	1.33900	2.38898	0.49357
C	-1.33952	2.38863	-0.49355
C	0.66444	3.54169	0.24180
C	-0.66526	3.54151	-0.24177
H	-2.34834	2.36410	-0.88304
H	2.34782	2.36471	0.88307
C	-1.40080	-0.07592	-0.55888
C	-0.96332	-1.11449	-1.33806
C	-2.72204	-0.28982	-0.06522
C	1.40090	-0.07557	0.55888
C	2.72216	-0.28920	0.06519
C	3.26799	-1.48176	0.43589
H	3.22197	0.41665	-0.58356
C	0.96366	-1.11421	1.33810
S	2.15302	-2.35816	1.42829
C	-3.26760	-1.48251	-0.43591
S	-2.15244	-2.35868	-1.42828
H	-3.22202	0.41587	0.58358
C	0.32203	-1.26760	-2.08226
H	0.79109	-0.29436	-2.21751
H	1.02166	-1.90294	-1.53489
H	0.15439	-1.71292	-3.06196
C	-0.32166	-1.26757	2.08230
H	-0.79096	-0.29443	2.21746
H	-1.02113	-1.90312	1.53497
H	-0.15392	-1.71277	3.06203
C	0.66367	5.04647	0.24290
C	-0.66491	5.04629	-0.24281
C	1.35174	6.19927	0.49453
C	-1.35331	6.19891	-0.49439

H	-2.36816	6.21835	-0.86570
C	0.64816	7.39730	0.23745
C	-0.65006	7.39713	-0.23727
H	2.36658	6.21898	0.86584
H	1.14008	8.34347	0.41764
H	-1.14226	8.34316	-0.41741
C	-4.57951	-2.02609	-0.08720
C	-4.89007	-3.37539	-0.25251
C	-5.58351	-1.20591	0.43021
C	-6.15029	-3.82988	0.09874
H	-4.16037	-4.07372	-0.63959
C	-6.80878	-1.76016	0.75529
H	-5.42317	-0.14616	0.56702
N	-7.10848	-3.05077	0.59901
H	-6.39747	-4.87736	-0.02679
H	-7.59309	-1.13004	1.15773
C	4.57999	-2.02511	0.08715
C	4.89112	-3.37417	0.25338
C	5.58352	-1.20498	-0.43124
C	6.15139	-3.82848	-0.09793
H	4.16185	-4.07250	0.64127
C	6.80890	-1.75902	-0.75628
H	5.42276	-0.14541	-0.56893
N	7.10914	-3.04941	-0.59910
H	6.39897	-4.87577	0.02831
H	7.59283	-1.12891	-1.15945

BPPyr-c

E = –2138.38494751 Ha (all vibrational frequencies real)

C	1.40191	0.05084	0.14141
C	0.63077	-1.22923	0.43057
C	2.81457	-0.18684	0.11414
C	-1.40191	0.05083	-0.14142
C	-2.81457	-0.18687	-0.11415
C	-3.17233	-1.48845	-0.06791
H	-3.54413	0.60823	-0.09001
C	-0.63076	-1.22924	-0.43058
S	-1.79958	-2.59859	-0.02890
C	3.17235	-1.48842	0.06790
S	1.79960	-2.59857	0.02890
H	3.54413	0.60826	0.08999
C	0.35324	-1.28308	1.94328
H	-0.30182	-0.46202	2.23528

H	-0.11197	-2.22446	2.22491
H	1.29726	-1.18334	2.47659
C	-0.35323	-1.28309	-1.94329
H	0.30183	-0.46202	-2.23529
H	0.11200	-2.22447	-2.22492
H	-1.29725	-1.18337	-2.47660
C	-0.74233	1.23555	-0.00405
C	0.74231	1.23556	0.00403
C	-1.46463	2.50056	0.09701
C	1.46461	2.50058	-0.09703
C	-0.73265	3.61543	0.06193
C	0.73261	3.61544	-0.06196
H	2.54266	2.50309	-0.16514
H	-2.54269	2.50306	0.16513
C	-0.70328	5.09827	0.06344
C	0.70324	5.09827	-0.06346
C	-1.43599	6.26363	0.13151
C	1.43594	6.26364	-0.13151
H	2.51200	6.28244	-0.22998
C	-0.69471	7.44738	0.06390
C	0.69466	7.44739	-0.06387
H	-2.51206	6.28241	0.22999
H	-1.21105	8.39651	0.11154
H	1.21099	8.39652	-0.11149
C	4.53460	-2.02024	0.01260
C	4.78043	-3.35880	-0.28814
C	5.64617	-1.21014	0.25638
C	6.08920	-3.81332	-0.34020
H	3.96994	-4.04611	-0.48836
C	6.91170	-1.76151	0.18020
H	5.53580	-0.16772	0.51675
N	7.14983	-3.04211	-0.11331
H	6.28877	-4.85167	-0.57629
H	7.78017	-1.14238	0.36990
C	-4.53458	-2.02028	-0.01259
C	-4.78040	-3.35885	0.28810
C	-5.64616	-1.21017	-0.25632
C	-6.08917	-3.81337	0.34017
H	-3.96991	-4.04617	0.48827
C	-6.91168	-1.76154	-0.18012
H	-5.53579	-0.16773	-0.51664
N	-7.14981	-3.04216	0.11335
H	-6.28873	-4.85173	0.57623
H	-7.78016	-1.14240	-0.36977

BPPyr-TS

$E = -2138.34457585$ Ha (one imaginary vibrational frequency)

C	0.63673	1.32469	0.30169
C	-0.63675	1.32468	-0.30170
C	1.29458	2.53838	0.61823
C	-1.29462	2.53836	-0.61823
C	0.63763	3.69197	0.30455
C	-0.63768	3.69196	-0.30455
H	-2.26189	2.51780	-1.10144
H	2.26185	2.51783	1.10144
C	-1.31023	0.04288	-0.55352
C	-0.53962	-1.15560	-0.81368
C	-2.64976	-0.19800	-0.30779
C	1.31023	0.04290	0.55352
C	2.64976	-0.19797	0.30778
C	3.03066	-1.53297	0.38922
H	3.33243	0.58201	0.00044
C	0.53963	-1.15560	0.81367
S	1.67007	-2.52429	0.83105
C	-3.03064	-1.53302	-0.38923
S	-1.67004	-2.52431	-0.83105
H	-3.33244	0.58196	-0.00045
C	-0.63788	5.19573	-0.30643
C	-1.29801	6.34779	-0.62439
C	-0.62249	7.54671	-0.29968
C	0.62239	7.54671	0.29969
C	1.29792	6.34781	0.62440
C	0.63781	5.19574	0.30643
H	-2.27195	6.36738	-1.09253
H	-1.09484	8.49263	-0.52719
H	1.09472	8.49265	0.52721
H	2.27186	6.36741	1.09254
C	0.45777	-1.19327	-1.95160
H	1.10257	-2.06927	-1.88307
H	-0.07873	-1.23458	-2.89819
H	1.07451	-0.29797	-1.95030
C	-0.45776	-1.19328	1.95160
H	-1.10253	-2.06929	1.88307
H	0.07875	-1.23457	2.89818
H	-1.07452	-0.29799	1.95029
C	4.32708	-2.10254	0.10305
C	4.53694	-3.48647	0.05661

C	5.44714	-1.29398	-0.13766
C	5.80049	-3.97543	-0.22139
H	3.72530	-4.18023	0.22875
C	6.66610	-1.88602	-0.40499
H	5.37711	-0.21647	-0.11070
N	6.86538	-3.20636	-0.45274
H	5.96318	-5.04613	-0.26014
H	7.53339	-1.26333	-0.59041
C	-4.32705	-2.10260	-0.10305
C	-4.53688	-3.48653	-0.05657
C	-5.44713	-1.29406	0.13763
C	-5.80042	-3.97551	0.22143
H	-3.72523	-4.18028	-0.22870
C	-6.66607	-1.88611	0.40497
H	-5.37712	-0.21655	0.11064
N	-6.86534	-3.20646	0.45275
H	-5.96310	-5.04622	0.26021
H	-7.53338	-1.26343	0.59036

[BPPyr-H]$^+$-o

E = –2138.86297291 Ha (all vibrational frequencies real)

C	0.58257	1.21020	0.25852
C	-0.71555	1.16174	-0.25340
C	1.22432	2.45724	0.51124
C	-1.44615	2.36073	-0.49732
C	0.50953	3.58481	0.25645
C	-0.81550	3.53635	-0.23783
H	-2.45070	2.30016	-0.89350
H	2.22956	2.46943	0.91050
C	-1.41339	-0.10576	-0.56608
C	-0.93254	-1.12240	-1.34874
C	-2.72461	-0.37597	-0.07268
C	1.37242	-0.00483	0.56314
C	2.68982	-0.18018	0.07266
C	3.27241	-1.36194	0.45043
H	3.16645	0.54413	-0.57249
C	0.96875	-1.06025	1.34986
S	2.18384	-2.26766	1.44608
C	-3.21783	-1.59025	-0.44570
S	-2.06719	-2.41621	-1.44070
H	-3.25419	0.30641	0.57757
C	0.35323	-1.21371	-2.10292
H	0.78866	-0.22236	-2.21650

H	1.07628	-1.84302	-1.57956
H	0.19344	-1.63763	-3.09331
C	-0.31024	-1.24349	2.09517
H	-0.80331	-0.28210	2.22695
H	-0.99005	-1.89805	1.54579
H	-0.13002	-1.68491	3.07396
C	0.45244	5.08838	0.26110
C	-0.87094	5.03995	-0.23524
C	1.09515	6.26539	0.52103
C	-1.60022	6.16646	-0.49009
H	-2.61204	6.14868	-0.86952
C	0.34931	7.43655	0.26071
C	-0.94432	7.38910	-0.22468
H	2.10547	6.32212	0.90065
H	0.80393	8.40002	0.44694
H	-1.46994	8.31657	-0.40669
C	-4.50526	-2.19120	-0.09812
C	-4.74947	-3.55579	-0.24666
C	-5.55037	-1.41224	0.40060
C	-5.98946	-4.06481	0.10214
H	-3.98412	-4.22365	-0.61838
C	-6.75054	-2.01962	0.72488
H	-5.44133	-0.34421	0.52266
N	-6.98704	-3.32502	0.58455
H	-6.18592	-5.12441	-0.01024
H	-7.56704	-1.42212	1.11265
C	4.58382	-1.85629	0.09909
C	4.99849	-3.15374	0.44125
C	5.49531	-1.04774	-0.60152
C	6.24672	-3.59208	0.09384
H	4.34651	-3.82922	0.97399
C	6.73119	-1.53111	-0.92790
H	5.24720	-0.03819	-0.88646
N	7.07913	-2.78092	-0.57732
H	6.61573	-4.57768	0.32821
H	7.47041	-0.95610	-1.46222
H	7.99946	-3.12126	-0.82730

[BPPyr-H]$^{+}$-c

E = –2138.82775370 Ha (all vibrational frequencies real)

C	1.37107	0.09036	0.13607
C	0.63435	-1.21029	0.42534
C	2.77857	-0.09708	0.09423

C	-1.42634	0.01681	-0.14682
C	-2.82911	-0.25803	-0.11953
C	-3.14772	-1.57224	-0.08109
H	-3.58177	0.51410	-0.08078
C	-0.62189	-1.23967	-0.44173
S	-1.75335	-2.64427	-0.06319
C	3.18366	-1.39356	0.05507
S	1.83990	-2.54535	0.01605
H	3.47636	0.72615	0.07964
C	0.34943	-1.27861	1.93520
H	-0.32203	-0.47245	2.23149
H	-0.09918	-2.23087	2.20776
H	1.28816	-1.16533	2.47491
C	-0.33952	-1.27042	-1.95533
H	0.29341	-0.42895	-2.23724
H	0.15233	-2.19614	-2.24284
H	-1.28422	-1.19166	-2.49064
C	-0.80177	1.22403	-0.00264
C	0.67695	1.26570	0.01191
C	-1.56573	2.46316	0.09290
C	1.36181	2.54685	-0.07312
C	-0.86872	3.60060	0.07102
C	0.59401	3.63996	-0.03568
H	2.43935	2.58620	-0.12886
H	-2.64364	2.43057	0.14837
C	-0.87808	5.08450	0.08215
C	0.52908	5.12096	-0.02644
C	-1.64015	6.23042	0.14863
C	1.23564	6.30285	-0.07642
H	2.31205	6.34742	-0.16044
C	-0.92576	7.43164	0.09918
C	0.46485	7.46736	-0.01007
H	-2.71738	6.22288	0.23303
H	-1.46554	8.36761	0.14692
H	0.95779	8.42928	-0.04371
C	4.54849	-1.86224	0.00949
C	4.83769	-3.23761	0.03682
C	5.63646	-0.97274	-0.07530
C	6.13495	-3.67086	-0.01447
H	4.05103	-3.97319	0.10568
C	6.91341	-1.45371	-0.12647
H	5.49257	0.09487	-0.10782
N	7.13538	-2.78128	-0.09500
H	6.41321	-4.71237	0.00675

H	7.78324	-0.82050	-0.19349
C	-4.49705	-2.13943	-0.02431
C	-4.70895	-3.47435	0.31343
C	-5.62352	-1.36506	-0.30633
C	-6.00589	-3.96147	0.36333
H	-3.88298	-4.13259	0.54560
C	-6.87569	-1.94769	-0.22931
H	-5.53546	-0.32900	-0.59855
N	-7.08222	-3.22424	0.10008
H	-6.18231	-4.99684	0.62830
H	-7.75768	-1.35843	-0.44859
H	8.08859	-3.11940	-0.13512

[BPPyr-H]$^{+}$-TS

E = –2138.79493832 Ha (one imaginary vibrational frequency)

C	0.35461	1.44108	0.28834
C	-0.89572	1.19713	-0.30794
C	0.76894	2.75487	0.61157
C	-1.77971	2.26022	-0.61783
C	-0.09989	3.76020	0.30255
C	-1.35350	3.51641	-0.30390
H	-2.72807	2.05522	-1.09493
H	1.72265	2.91935	1.09366
C	-1.31015	-0.19003	-0.54841
C	-0.32516	-1.22046	-0.80492
C	-2.58121	-0.68158	-0.30686
C	1.26049	0.30609	0.52935
C	2.60645	0.31451	0.28775
C	3.23263	-0.94455	0.37957
H	3.13553	1.20509	-0.02055
C	0.71648	-1.01503	0.80630
S	2.06884	-2.15746	0.83241
C	-2.68941	-2.06452	-0.38230
S	-1.16946	-2.77997	-0.80712
H	-3.40391	-0.04931	-0.00519
C	-1.64311	4.99207	-0.29807
C	-2.51520	5.99590	-0.60828
C	-2.08227	7.30011	-0.27895
C	-0.85796	7.53777	0.31716
C	0.03722	6.49142	0.63406
C	-0.39018	5.23517	0.31132
H	-3.47631	5.82899	-1.07331
H	-2.72899	8.13834	-0.49946

H	-0.57675	8.55615	0.54811
H	0.99062	6.69612	1.09990
C	0.64961	-1.06989	-1.95477
H	1.45826	-1.79675	-1.88348
H	0.12189	-1.23344	-2.89255
H	1.06933	-0.06747	-1.97220
C	-0.24841	-1.21013	1.95720
H	-0.73096	-2.18578	1.90816
H	0.30123	-1.14581	2.89451
H	-1.00978	-0.43447	1.95624
C	4.58079	-1.25863	0.10590
C	5.06062	-2.59545	0.13162
C	5.53338	-0.25056	-0.20330
C	6.36694	-2.86765	-0.13143
H	4.40057	-3.41965	0.35681
C	6.82795	-0.57892	-0.45709
H	5.25435	0.79015	-0.23895
N	7.22957	-1.86963	-0.41953
H	6.77332	-3.86642	-0.12482
H	7.58440	0.15139	-0.69595
C	-3.86047	-2.87395	-0.10380
C	-3.78936	-4.26662	-0.00548
C	-5.12036	-2.29110	0.07204
C	-4.94078	-4.98662	0.26016
H	-2.85071	-4.79017	-0.12543
C	-6.20840	-3.10325	0.33297
H	-5.26131	-1.22292	-0.00203
N	-6.14124	-4.43271	0.43070
H	-4.89226	-6.06587	0.34030
H	-7.18751	-2.66011	0.46915
H	8.19692	-2.09174	-0.60806

[BPPyr-H$_2$]$^{2+}$-o

E = –2139.30523110 Ha (all vibrational frequencies real)

C	-0.64825	1.20151	-0.25817
C	0.64821	1.20157	0.25825
C	-1.33427	2.42446	-0.51089
C	1.33413	2.42459	0.51093
C	-0.66181	3.57649	-0.25065
C	0.66158	3.57655	0.25063
H	2.33754	2.40089	0.91394
H	-2.33767	2.40066	-0.91390
C	1.39058	-0.04181	0.56675

C	0.94825	-1.07727	1.35860
C	2.70067	-0.26818	0.07577
C	-1.39051	-0.04194	-0.56664
C	-2.70058	-0.26841	-0.07563
C	-3.23730	-1.46959	-0.45752
H	-3.20388	0.43457	0.57275
C	-0.94811	-1.07738	-1.35847
S	-2.11669	-2.33011	-1.45753
C	3.23743	-1.46937	0.45757
S	2.11691	-2.32993	1.45765
H	3.20392	0.43484	-0.57261
C	-0.33213	-1.20406	2.11412
H	-0.79406	-0.22510	2.22751
H	-1.03454	-1.85309	1.58723
H	-0.15898	-1.62700	3.10228
C	0.33228	-1.20409	-2.11397
H	0.79412	-0.22510	-2.22742
H	1.03475	-1.85302	-1.58703
H	0.15919	-1.62713	-3.10211
C	-0.66079	5.08095	-0.25177
C	0.66043	5.08102	0.25170
C	-1.34620	6.23332	-0.51317
C	1.34573	6.23346	0.51309
H	2.35529	6.25293	0.89831
C	-0.64630	7.43070	-0.24636
C	0.64572	7.43077	0.24625
H	-2.35576	6.25270	-0.89839
H	-1.13553	8.37689	-0.43318
H	1.13487	8.37701	0.43304
C	4.53106	-2.01345	0.10874
C	4.88788	-3.33163	0.43481
C	5.48030	-1.23266	-0.57203
C	6.11945	-3.81661	0.08918
H	4.20405	-3.98737	0.95211
C	6.69760	-1.76269	-0.89748
H	5.27701	-0.20875	-0.84096
N	6.98931	-3.03096	-0.56401
H	6.44561	-4.82027	0.31022
H	7.46472	-1.21171	-1.41752
C	-4.53094	-2.01370	-0.10878
C	-4.88771	-3.33188	-0.43491
C	-5.48023	-1.23296	0.57198
C	-6.11929	-3.81690	-0.08935
H	-4.20384	-3.98758	-0.95220

C	-6.69754	-1.76303	0.89735
H	-5.27698	-0.20906	0.84097
N	-6.98921	-3.03129	0.56382
H	-6.44542	-4.82056	-0.31044
H	-7.46469	-1.21209	1.41737
H	-7.89572	-3.40677	0.81368
H	7.89583	-3.40640	-0.81392

[BPPyr-H$_2$]$^{2+}$-c

E = –2139.26687466 Ha (all vibrational frequencies real)

C	1.39696	0.06305	0.14063
C	0.62884	-1.21744	0.43273
C	2.80375	-0.16421	0.10839
C	-1.39696	0.06305	-0.14063
C	-2.80375	-0.16421	-0.10839
C	-3.16615	-1.47032	-0.06749
H	-3.52432	0.63856	-0.09396
C	-0.62883	-1.21744	-0.43273
S	-1.79836	-2.58510	-0.02941
C	3.16615	-1.47031	0.06749
S	1.79837	-2.58510	0.02941
H	3.52432	0.63856	0.09397
C	0.35075	-1.27201	1.94518
H	-0.30360	-0.45126	2.23934
H	-0.11442	-2.21396	2.22462
H	1.29404	-1.17353	2.47972
C	-0.35074	-1.27201	-1.94518
H	0.30360	-0.45125	-2.23934
H	0.11442	-2.21395	-2.22463
H	-1.29404	-1.17352	-2.47972
C	-0.74083	1.25603	-0.00596
C	0.74083	1.25603	0.00598
C	-1.46559	2.51560	0.08861
C	1.46559	2.51560	-0.08859
C	-0.73172	3.63044	0.05635
C	0.73172	3.63044	-0.05633
H	2.54343	2.52037	-0.14916
H	-2.54344	2.52037	0.14917
C	-0.70342	5.11212	0.05724
C	0.70341	5.11212	-0.05722
C	-1.43864	6.27632	0.11859
C	1.43863	6.27632	-0.11860
H	2.51530	6.29458	-0.20735

C	-0.69576	7.45832	0.05759
C	0.69574	7.45832	-0.05762
H	-2.51531	6.29458	0.20734
H	-1.21141	8.40789	0.10045
H	1.21139	8.40789	-0.10050
C	4.52393	-1.98127	0.02141
C	4.76733	-3.36219	-0.01117
C	5.63465	-1.12142	0.00074
C	6.05309	-3.83297	-0.06203
H	3.95827	-4.07611	0.00718
C	6.89943	-1.63846	-0.05051
H	5.52238	-0.04986	0.02468
N	7.07831	-2.97081	-0.08081
H	6.30069	-4.88210	-0.08816
H	7.78933	-1.03025	-0.06834
C	-4.52393	-1.98127	-0.02141
C	-4.76733	-3.36219	0.01115
C	-5.63464	-1.12143	-0.00073
C	-6.05309	-3.83298	0.06201
H	-3.95827	-4.07611	-0.00721
C	-6.89943	-1.63847	0.05052
H	-5.52238	-0.04986	-0.02466
N	-7.07830	-2.97082	0.08080
H	-6.30068	-4.88211	0.08813
H	-7.78933	-1.03026	0.06835
H	8.02196	-3.33652	-0.11844
H	-8.02195	-3.33654	0.11844

[BPPyr-H_2]$^{2+}$-TS

E = –2139.23841526 Ha (one imaginary vibrational frequency)

C	0.63662	1.35812	0.29714
C	-0.63662	1.35812	-0.29714
C	1.30040	2.56947	0.61251
C	-1.30039	2.56947	-0.61251
C	0.63960	3.72016	0.30276
C	-0.63959	3.72016	-0.30276
H	-2.26986	2.54913	-1.09059
H	2.26986	2.54912	1.09059
C	-1.30763	0.07404	-0.53876
C	-0.53600	-1.12008	-0.82598
C	-2.63672	-0.17110	-0.29405
C	1.30763	0.07404	0.53876
C	2.63672	-0.17111	0.29405

C	3.00602	-1.52036	0.38556
H	3.32242	0.60292	-0.01856
C	0.53600	-1.12008	0.82598
S	1.64342	-2.49675	0.83568
C	-3.00603	-1.52035	-0.38556
S	-1.64343	-2.49674	-0.83568
H	-3.32242	0.60293	0.01856
C	-0.63793	5.22309	-0.30442
C	-1.30014	6.37489	-0.62165
C	-0.62373	7.57170	-0.29851
C	0.62376	7.57170	0.29851
C	1.30017	6.37489	0.62165
C	0.63795	5.22309	0.30442
H	-2.27503	6.39403	-1.08743
H	-1.09587	8.51797	-0.52445
H	1.09590	8.51796	0.52445
H	2.27505	6.39402	1.08743
C	0.46343	-1.13828	-1.96117
H	1.13225	-1.99510	-1.88591
H	-0.07535	-1.20676	-2.90452
H	1.04997	-0.22366	-1.97192
C	-0.46343	-1.13828	1.96117
H	-1.13226	-1.99510	1.88591
H	0.07534	-1.20676	2.90452
H	-1.04997	-0.22365	1.97192
C	4.28499	-2.08504	0.11127
C	4.49000	-3.48330	0.08843
C	5.40905	-1.26570	-0.14228
C	5.72681	-3.99428	-0.17483
H	3.67803	-4.17108	0.27028
C	6.62443	-1.82752	-0.39763
H	5.33537	-0.19038	-0.13171
N	6.76355	-3.16836	-0.41122
H	5.93540	-5.05165	-0.20754
H	7.51286	-1.24915	-0.59473
C	-4.28500	-2.08502	-0.11127
C	-4.49001	-3.48328	-0.08843
C	-5.40906	-1.26568	0.14229
C	-5.72682	-3.99426	0.17483
H	-3.67805	-4.17106	-0.27029
C	-6.62444	-1.82749	0.39763
H	-5.33537	-0.19036	0.13172
N	-6.76356	-3.16834	0.41122
H	-5.93542	-5.05163	0.20754

H	-7.51287	-1.24912	0.59474
H	7.67365	-3.56516	-0.60326
H	-7.67366	-3.56513	0.60326

[BPPyr-Me]$^{+}$-o

E = –2178.16538555 Ha (all vibrational frequencies real)

C	-1.17867	1.06305	-0.25501
C	0.08342	1.33964	0.27437
C	-2.10391	2.11494	-0.51668
C	0.49359	2.68089	0.52674
C	-1.69244	3.38383	-0.25634
C	-0.40332	3.66513	0.25498
H	1.47574	2.86978	0.93870
H	-3.07652	1.87808	-0.92621
C	1.06927	0.28397	0.60020
C	0.84475	-0.82327	1.38580
C	2.40728	0.34521	0.13519
C	-1.63997	-0.30811	-0.56861
C	-2.89148	-0.80007	-0.09213
C	-3.16024	-2.08372	-0.46254
H	-3.54140	-0.21589	0.54444
C	-0.97710	-1.22966	-1.33588
S	-1.86800	-2.70142	-1.43428
C	3.18316	-0.71113	0.53226
S	2.25501	-1.79428	1.51192
H	2.76066	1.14086	-0.50534
C	-0.39494	-1.22896	2.10984
H	-1.05291	-0.36983	2.22735
H	-0.93836	-1.99517	1.55297
H	-0.15766	-1.62816	3.09460
C	0.31854	-1.10138	-2.06739
H	0.57546	-0.05017	-2.18708
H	1.13075	-1.58780	-1.52275
H	0.25460	-1.55763	-3.05424
C	-2.00967	4.85467	-0.26423
C	-0.72205	5.13559	0.24886
C	-2.92083	5.83544	-0.53561
C	-0.29837	6.40784	0.50929
H	0.68119	6.64160	0.90161
C	-2.49213	7.15500	-0.26992
C	-1.23344	7.42970	0.23164
H	-3.90876	5.63974	-0.92774
H	-3.16901	7.97565	-0.46484

H	-0.95659	8.45853	0.41735
C	4.57178	-0.96150	0.21504
C	5.20694	-2.16120	0.55841
C	5.34658	0.00003	-0.45348
C	6.52529	-2.36181	0.24288
H	4.67927	-2.95264	1.06941
C	6.65686	-0.25456	-0.74478
H	4.93458	0.95419	-0.74103
N	7.23598	-1.42125	-0.39973
H	7.04696	-3.27305	0.49020
H	7.28889	0.45617	-1.25526
C	-4.32811	-2.89823	-0.12767
C	-4.33836	-4.28235	-0.29549
C	-5.49037	-2.31460	0.37862
C	-5.47459	-4.99858	0.04272
H	-3.47158	-4.80666	-0.67470
C	-6.57070	-3.12057	0.69111
H	-5.56393	-1.24560	0.51705
N	-6.58319	-4.44510	0.53238
H	-5.48885	-6.07446	-0.08497
H	-7.47638	-2.67512	1.08525
C	8.65310	-1.64011	-0.73129
H	8.78161	-1.54432	-1.80517
H	9.25428	-0.89775	-0.21470
H	8.93710	-2.63548	-0.41102

[BPPyr-Me]$^{+}$-c

E = –2178.13027005 Ha (all vibrational frequencies real)

C	1.05290	0.39290	0.14440
C	0.57561	-1.02529	0.42553
C	2.47261	0.47550	0.11840
C	-1.67925	-0.20718	-0.15072
C	-3.00575	-0.74098	-0.12521
C	-3.07238	-2.09111	-0.08375
H	-3.88977	-0.12347	-0.08839
C	-0.65188	-1.29040	-0.44264
S	-1.50068	-2.88213	-0.06213
C	3.11450	-0.71973	0.07536
S	2.01482	-2.10456	0.01235
H	3.00237	1.41567	0.10873
C	0.30905	-1.15310	1.93493
H	-0.50514	-0.49206	2.23282
H	0.05153	-2.17469	2.20366

H	1.20815	-0.86304	2.47607
C	-0.36641	-1.26928	-1.95555
H	0.09880	-0.32457	-2.23721
H	0.29006	-2.08701	-2.24185
H	-1.30841	-1.36853	-2.49225
C	-1.29373	1.09554	-0.00744
C	0.15126	1.41509	0.01437
C	-2.27717	2.16958	0.08150
C	0.58357	2.80301	-0.06788
C	-1.80613	3.41752	0.06102
C	-0.37580	3.73225	-0.03637
H	1.63514	3.04302	-0.11808
H	-3.33019	1.93538	0.13042
C	-2.09552	4.87262	0.06734
C	-0.71985	5.17442	-0.03130
C	-3.06146	5.85356	0.12356
C	-0.24983	6.46888	-0.07978
H	0.79927	6.71663	-0.15584
C	-2.58741	7.16822	0.07542
C	-1.22779	7.46639	-0.02298
H	-4.11841	5.64205	0.19938
H	-3.29510	7.98509	0.11512
H	-0.92565	8.50422	-0.05598
C	4.54552	-0.92132	0.03539
C	5.09419	-2.20777	-0.04761
C	5.44982	0.15480	0.07117
C	6.45397	-2.38026	-0.09550
H	4.46685	-3.08584	-0.07317
C	6.79436	-0.07573	0.02132
H	5.11294	1.17655	0.14138
N	7.28747	-1.32970	-0.06219
H	6.91143	-3.35503	-0.16011
H	7.52325	0.71999	0.04710
C	-4.29105	-2.90136	-0.02351
C	-4.24845	-4.25093	0.31998
C	-5.54359	-2.35357	-0.30569
C	-5.43093	-4.97239	0.37560
H	-3.31340	-4.74157	0.55314
C	-6.66408	-3.16029	-0.22263
H	-5.65220	-1.32082	-0.60260
N	-6.62710	-4.45146	0.11261
H	-5.40927	-6.02122	0.64554
H	-7.64143	-2.74781	-0.44157
C	8.74480	-1.51994	-0.13061

H	9.11099	-1.10917	-1.06738
H	9.20494	-1.00630	0.70782
H	8.96220	-2.58024	-0.07988

[BPPyr-Me]+-TS

E = –2178.09657474 Ha (one imaginary vibrational frequency)

C	-0.20192	1.49401	0.30780
C	-1.37498	1.01897	-0.30642
C	-0.04718	2.86261	0.63298
C	-2.43817	1.89708	-0.63221
C	-1.08477	3.68662	0.30856
C	-2.26092	3.21110	-0.31547
H	-3.32352	1.51728	-1.12313
H	0.85147	3.20350	1.12827
C	-1.51738	-0.42134	-0.55122
C	-0.35284	-1.24719	-0.79381
C	-2.67552	-1.14417	-0.32309
C	0.89834	0.55076	0.56503
C	2.22275	0.81522	0.34158
C	3.07387	-0.30080	0.44419
H	2.57718	1.78979	0.03713
C	0.61245	-0.84894	0.83662
S	2.15691	-1.71391	0.88140
C	-2.52071	-2.52263	-0.39664
S	-0.88738	-2.93785	-0.80470
H	-3.60555	-0.67822	-0.03011
C	-2.82290	4.60592	-0.31913
C	-3.86316	5.42770	-0.64612
C	-3.68836	6.79038	-0.31508
C	-2.53991	7.25441	0.29833
C	-1.46889	6.39522	0.63237
C	-1.64735	5.08075	0.30799
H	-4.76842	5.08287	-1.12516
H	-4.47755	7.49185	-0.54868
H	-2.45851	8.30768	0.52962
H	-0.57832	6.77605	1.11168
C	0.59420	-0.91353	-1.92768
H	1.52577	-1.47233	-1.84155
H	0.12298	-1.17449	-2.87353
H	0.81515	0.15059	-1.94154
C	-0.31543	-1.22759	1.97175
H	-0.60709	-2.27572	1.91179
H	0.19818	-1.06774	2.91810

H	-1.20803	-0.60747	1.96129
C	4.46375	-0.35041	0.18916
C	5.19110	-1.56628	0.20527
C	5.21445	0.81771	-0.09335
C	6.52961	-1.57857	-0.04348
H	4.70144	-2.50731	0.40718
C	6.55239	0.74067	-0.33205
H	4.74972	1.79024	-0.12257
N	7.21000	-0.44193	-0.31285
H	7.11015	-2.48842	-0.04030
H	7.15064	1.61240	-0.54822
C	-3.52006	-3.53807	-0.12703
C	-3.18484	-4.88894	0.00583
C	-4.87350	-3.20726	0.00556
C	-4.18264	-5.81282	0.26182
H	-2.15968	-5.22280	-0.07834
C	-5.79099	-4.20934	0.26043
H	-5.21604	-2.18837	-0.09931
N	-5.47174	-5.49887	0.39137
H	-3.92883	-6.86058	0.36890
H	-6.84064	-3.96137	0.36249
C	8.66117	-0.48862	-0.52157
H	8.95831	0.36854	-1.11603
H	9.16869	-0.46562	0.44005
H	8.91227	-1.40250	-1.05031

[BPPyr-Me$_2$]$^{2+}$-o

E = –2217.91005793 Ha (all vibrational frequencies real)

C	-0.64078	1.40870	-0.27608
C	0.64094	1.40861	0.27649
C	-1.31914	2.63193	-0.54771
C	1.31952	2.63175	0.54800
C	-0.65414	3.78395	-0.26891
C	0.65473	3.78386	0.26907
H	2.31143	2.60802	0.97856
H	-2.31105	2.60834	-0.97829
C	1.37483	0.16524	0.60484
C	0.91021	-0.87204	1.37996
C	2.69972	-0.05922	0.15074
C	-1.37484	0.16543	-0.60444
C	-2.69977	-0.05887	-0.15038
C	-3.22516	-1.26052	-0.54292
H	-3.22126	0.64588	0.48158

C	-0.91035	-0.87187	-1.37960
S	-2.07701	-2.12512	-1.50709
C	3.22494	-1.26098	0.54318
S	2.07674	-2.12543	1.50742
H	3.22127	0.64547	-0.48123
C	-0.39123	-1.00196	2.09835
H	-0.85620	-0.02340	2.20268
H	-1.07885	-1.64828	1.54902
H	-0.24644	-1.42859	3.08953
C	0.39110	-1.00192	-2.09797
H	0.85619	-0.02342	-2.20226
H	1.07863	-1.64834	-1.54863
H	0.24628	-1.42851	-3.08916
C	-0.65291	5.28845	-0.27011
C	0.65382	5.28836	0.27002
C	-1.33052	6.44093	-0.55060
C	1.33168	6.44075	0.55026
H	2.33015	6.46009	0.96338
C	-0.63816	7.63830	-0.26451
C	0.63959	7.63821	0.26392
H	-2.32898	6.46041	-0.96374
H	-1.12187	8.58453	-0.46502
H	1.12352	8.58438	0.46423
C	4.53040	-1.80399	0.23342
C	4.87451	-3.12661	0.53872
C	5.51225	-1.01847	-0.38933
C	6.12172	-3.60492	0.23460
H	4.17151	-3.79823	1.00824
C	6.74047	-1.54853	-0.67138
H	5.32907	0.01226	-0.64778
N	7.03812	-2.82495	-0.36178
H	6.42219	-4.61784	0.45297
H	7.52456	-0.97828	-1.14558
C	-4.53072	-1.80336	-0.23333
C	-4.87509	-3.12583	-0.53916
C	-5.51240	-1.01795	0.38977
C	-6.12235	-3.60401	-0.23521
H	-4.17221	-3.79739	-1.00895
C	-6.74073	-1.54790	0.67165
H	-5.32902	0.01263	0.64869
N	-7.03862	-2.82410	0.36154
H	-6.42308	-4.61678	-0.45396
H	-7.52465	-0.97764	1.14614
C	-8.38913	-3.33859	0.64206

H	-8.69592	-2.99694	1.62510
H	-9.07341	-2.96488	-0.11481
H	-8.36260	-4.42172	0.61896
C	8.38846	-3.33931	-0.64332
H	8.69173	-3.00332	-1.62944
H	9.07455	-2.95994	0.10906
H	8.36356	-4.42230	-0.61338

[BPPyr-Me$_2$]$^{2+}$-c

E = –2217.87237349 Ha (all vibrational frequencies real)

C	1.39689	0.26447	0.14133
C	0.62938	-1.01692	0.43227
C	2.80446	0.03766	0.11231
C	-1.39689	0.26446	-0.14102
C	-2.80445	0.03764	-0.11201
C	-3.16757	-1.26743	-0.07023
H	-3.52483	0.84067	-0.10146
C	-0.62937	-1.01693	-0.43193
S	-1.80109	-2.38333	-0.02710
C	3.16758	-1.26742	0.07056
S	1.80110	-2.38332	0.02743
H	3.52484	0.84068	0.10167
C	0.35215	-1.07302	1.94474
H	-0.30344	-0.25340	2.23942
H	-0.11153	-2.01580	2.22385
H	1.29553	-0.97337	2.47896
C	-0.35213	-1.07303	-1.94440
H	0.30344	-0.25341	-2.23908
H	0.11158	-2.01581	-2.22349
H	-1.29550	-0.97342	-2.47861
C	-0.74079	1.45665	-0.00613
C	0.74079	1.45664	0.00637
C	-1.46553	2.71683	0.08837
C	1.46553	2.71682	-0.08824
C	-0.73186	3.83163	0.05634
C	0.73186	3.83163	-0.05632
H	2.54341	2.72146	-0.14905
H	-2.54340	2.72147	0.14919
C	-0.70342	5.31345	0.05727
C	0.70342	5.31344	-0.05744
C	-1.43846	6.47776	0.11872
C	1.43845	6.47775	-0.11907
H	2.51512	6.49607	-0.20809

C	-0.69569	7.65993	0.05750
C	0.69567	7.65992	-0.05803
H	-2.51513	6.49608	0.20773
H	-1.21144	8.60945	0.10045
H	1.21142	8.60944	-0.10113
C	4.52674	-1.77677	0.03068
C	4.78264	-3.15126	-0.00392
C	5.63740	-0.91849	0.01950
C	6.07455	-3.61411	-0.04347
H	3.98144	-3.87462	0.00423
C	6.90205	-1.43404	-0.02052
H	5.52740	0.15355	0.04311
N	7.11242	-2.76594	-0.05046
H	6.31175	-4.66608	-0.06959
H	7.78483	-0.81337	-0.02886
C	-4.52673	-1.77678	-0.03052
C	-4.78267	-3.15129	0.00322
C	-5.63738	-0.91848	-0.01870
C	-6.07459	-3.61414	0.04240
H	-3.98148	-3.87465	-0.00544
C	-6.90204	-1.43403	0.02094
H	-5.52736	0.15358	-0.04151
N	-7.11244	-2.76595	0.04988
H	-6.31180	-4.66612	0.06775
H	-7.78481	-0.81334	0.02973
C	8.49633	-3.26773	-0.07709
H	9.01026	-2.83453	-0.92978
H	8.99168	-2.97932	0.84559
H	8.47453	-4.34720	-0.16604
C	-8.49637	-3.26769	0.07659
H	-9.00874	-2.83855	0.93229
H	-8.99332	-2.97483	-0.84380
H	-8.47453	-4.34758	0.16026

$[BPPyr\text{-}Me_2]^{2+}$-TS

E = –2217.84258971 Ha (one imaginary vibrational frequency)

C	0.62966	1.58042	0.30605
C	-0.63134	1.58080	-0.31442
C	1.28606	2.79162	0.63649
C	-1.28861	2.79255	-0.64128
C	0.63149	3.94281	0.31501
C	-0.63505	3.94328	-0.31608
H	-2.24813	2.77248	-1.13905

H	2.24548	2.77074	1.13443
C	-1.29715	0.29682	-0.57214
C	-0.51845	-0.89638	-0.84242
C	-2.63137	0.05020	-0.35472
C	1.29581	0.29623	0.56164
C	2.63010	0.04996	0.34449
C	2.99737	-1.29831	0.44699
H	3.32321	0.82383	0.04782
C	0.51724	-0.89702	0.83144
S	1.62455	-2.27404	0.86830
C	-2.99809	-1.29828	-0.45618
S	-1.62554	-2.27353	-0.87924
H	-3.32439	0.82350	-0.05633
C	-0.63443	5.44635	-0.31468
C	-1.29092	6.59846	-0.64226
C	-0.62182	7.79515	-0.30318
C	0.61351	7.79460	0.31835
C	1.28414	6.59731	0.65221
C	0.62917	5.44581	0.31950
H	-2.25646	6.61812	-1.12711
H	-1.09013	8.74158	-0.53631
H	1.08057	8.74061	0.55570
H	2.24959	6.61607	1.13731
C	0.50575	-0.91370	-1.95538
H	1.17460	-1.76898	-1.86395
H	-0.01142	-0.98423	-2.91060
H	1.09067	0.00204	-1.95396
C	-0.50700	-0.91489	1.94439
H	-1.17497	-1.77090	1.85331
H	0.01029	-0.98463	2.89960
H	-1.09284	0.00026	1.94273
C	4.28291	-1.86512	0.20594
C	4.50071	-3.25772	0.22481
C	5.40960	-1.05508	-0.05690
C	5.74690	-3.76788	-0.00491
H	3.69450	-3.95019	0.41542
C	6.62890	-1.62248	-0.27789
H	5.33742	0.02027	-0.08481
N	6.79793	-2.96322	-0.25404
H	5.94723	-4.82796	0.00179
H	7.51343	-1.03753	-0.47959
C	-4.28223	-1.86576	-0.20913
C	-4.49525	-3.25986	-0.21078
C	-5.41077	-1.05697	0.04572

C	-5.73814	-3.77095	0.02967
H	-3.68642	-3.95152	-0.39310
C	-6.62822	-1.62596	0.27748
H	-5.34254	0.01893	0.06015
N	-6.79192	-2.96666	0.27331
H	-5.93587	-4.83159	0.03873
H	-7.51307	-1.04049	0.47453
C	8.13975	-3.52050	-0.47104
H	8.56323	-3.08146	-1.36922
H	8.76556	-3.28971	0.38713
H	8.05764	-4.59443	-0.59120
C	-8.12900	-3.53825	0.48208
H	-8.02278	-4.55095	0.85486
H	-8.65924	-2.93311	1.20985
H	-8.66890	-3.54409	-0.46157

BPPip-o

E = –2143.19649100 Ha (all vibrational frequencies real)

C	-0.60296	1.22988	-0.35322
C	0.68487	1.07378	0.16823
C	-1.15770	2.52942	-0.54466
C	1.48779	2.21186	0.47429
C	-0.37545	3.59556	-0.23066
C	0.93908	3.43807	0.26809
H	2.48252	2.06677	0.87379
H	-2.15727	2.62517	-0.94695
C	1.30243	-0.24605	0.43226
C	0.74891	-1.26894	1.14690
C	2.61906	-0.56213	-0.04554
C	-1.46945	0.09106	-0.73314
C	-2.82401	-0.00445	-0.26781
C	-3.48192	-1.11288	-0.69175
H	-3.26510	0.73588	0.38518
C	-1.12447	-0.94875	-1.54701
S	-2.44211	-2.05795	-1.70643
C	3.04193	-1.81049	0.27476
S	1.82490	-2.62292	1.20203
H	3.20562	0.12868	-0.63562
C	-0.57111	-1.34424	1.84201
H	-0.95662	-0.34103	2.01828
H	-1.30761	-1.88007	1.23850
H	-0.47950	-1.85422	2.80030
C	0.15779	-1.21432	-2.26521

H	0.73496	-0.29449	-2.34822
H	0.76989	-1.94164	-1.72726
H	-0.02796	-1.59958	-3.26719
C	-0.22257	5.09110	-0.16183
C	1.09159	4.93365	0.33860
C	-0.78567	6.31828	-0.36781
C	1.88897	5.99792	0.65008
H	2.89476	5.89730	1.03286
C	0.03170	7.42614	-0.04852
C	1.31536	7.27239	0.43994
H	-1.78732	6.45820	-0.74910
H	-0.35891	8.42457	-0.19109
H	1.89851	8.15412	0.66851
C	4.34179	-2.48465	-0.04728
C	5.14881	-2.79298	1.21677
C	5.22424	-1.65673	-0.97417
C	6.56944	-3.17945	0.90258
H	5.16593	-1.91582	1.87187
C	6.47351	-2.44928	-1.33088
H	4.68960	-1.38355	-1.88436
N	7.17550	-3.05572	-0.19964
H	7.13148	-3.61929	1.72681
H	6.22411	-3.25736	-2.02425
C	-4.88179	-1.56594	-0.40045
C	-4.94097	-2.95280	0.24386
C	-5.61709	-0.59690	0.51675
C	-6.39230	-3.38361	0.39944
H	-4.40412	-3.68832	-0.35590
C	-6.91101	-1.17627	1.02391
H	-4.99852	-0.35350	1.38692
N	-7.28533	-2.38275	0.98231
H	-6.81006	-3.65642	-0.57386
H	-7.59829	-0.46358	1.48016
H	-5.82722	0.34960	0.01610
H	-4.45119	-2.90584	1.22087
H	-6.46001	-4.27731	1.01986
H	-5.43053	-1.62037	-1.34709
H	4.12706	-3.43458	-0.54755
H	5.50743	-0.73022	-0.46675
H	7.19008	-1.81362	-1.85105
H	4.68949	-3.59558	1.79673

BPPip-c

E = –2143.16654615 Ha (all vibrational frequencies real)

C	1.39007	0.03582	0.24986
C	0.59637	-1.24384	0.47682
C	2.80849	-0.20883	0.33445
C	-1.39007	0.03582	-0.24986
C	-2.80849	-0.20883	-0.33445
C	-3.16227	-1.50583	-0.30932
H	-3.54278	0.58095	-0.38976
C	-0.59637	-1.24384	-0.47682
S	-1.79425	-2.61292	-0.16597
C	3.16227	-1.50583	0.30932
S	1.79425	-2.61292	0.16597
H	3.54278	0.58095	0.38976
C	0.20249	-1.29698	1.96301
H	-0.47433	-0.47609	2.20119
H	-0.28260	-2.23862	2.20853
H	1.10231	-1.19540	2.56790
C	-0.20249	-1.29698	-1.96301
H	0.47433	-0.47609	-2.20119
H	0.28260	-2.23862	-2.20853
H	-1.10231	-1.19540	-2.56790
C	-0.74199	1.21457	-0.06080
C	0.74199	1.21457	0.06080
C	-1.46849	2.48280	-0.01110
C	1.46849	2.48280	0.01110
C	-0.73638	3.59735	0.00876
C	0.73638	3.59735	-0.00876
H	2.54908	2.48387	0.02151
H	-2.54908	2.48387	-0.02151
C	-0.70625	5.08024	0.01272
C	0.70625	5.08024	-0.01272
C	-1.44103	6.24645	0.02780
C	1.44103	6.24645	-0.02780
H	2.52151	6.26558	-0.04853
C	-0.69736	7.43076	0.01366
C	0.69736	7.43076	-0.01366
H	-2.52151	6.26558	0.04853
H	-1.21614	8.37976	0.02381
H	1.21614	8.37976	-0.02381
C	4.53901	-2.08835	0.35760
C	4.83215	-2.92152	-0.89446
C	5.64165	-1.04709	0.50284
C	6.29266	-3.26686	-1.01346
H	4.25559	-3.84838	-0.90163

C	6.98556	-1.74763	0.64221
H	5.64980	-0.40548	-0.38255
N	7.25579	-2.78574	-0.35195
H	6.53548	-4.02534	-1.75786
H	7.79783	-1.02356	0.58127
C	-4.53901	-2.08835	-0.35760
C	-4.83215	-2.92152	0.89446
C	-5.64165	-1.04709	-0.50284
C	-6.29266	-3.26686	1.01346
H	-4.25559	-3.84837	0.90163
C	-6.98556	-1.74763	-0.64221
H	-5.64980	-0.40548	0.38254
N	-7.25579	-2.78574	0.35194
H	-6.53548	-4.02534	1.75785
H	-7.79783	-1.02356	-0.58127
H	4.58431	-2.75991	1.22248
H	5.46907	-0.41097	1.37118
H	7.06797	-2.21612	1.62689
H	4.53315	-2.36966	-1.79170
H	-4.58431	-2.75991	-1.22248
H	-4.53315	-2.36966	1.79170
H	-5.46907	-0.41097	-1.37118
H	-7.06797	-2.21612	-1.62689

BPPip-TS

E = –2143.11461636 Ha (one imaginary vibrational frequency)

C	-0.67350	1.27875	-0.34993
C	0.55312	1.33950	0.35555
C	-1.36455	2.46310	-0.70679
C	1.12342	2.58592	0.71366
C	-0.79211	3.64789	-0.34430
C	0.43673	3.70862	0.35166
H	2.05135	2.61280	1.26895
H	-2.29061	2.39811	-1.26205
C	1.25917	0.09656	0.67685
C	0.53250	-1.14234	0.85173
C	2.64459	-0.07915	0.54913
C	-1.25307	-0.02716	-0.67326
C	-2.61560	-0.33744	-0.55501
C	-2.91947	-1.67090	-0.66353
H	-3.36585	0.40856	-0.32875
C	-0.40750	-1.18941	-0.84503
S	-1.48233	-2.60873	-0.96614

C	3.07928	-1.37688	0.65229
S	1.74155	-2.44804	0.97237
H	3.31204	0.74148	0.32360
C	0.36385	5.21000	0.35272
C	0.94324	6.39269	0.71260
C	0.23358	7.55918	0.34189
C	-0.96625	7.49996	-0.33943
C	-1.55889	6.26922	-0.70795
C	-0.86693	5.14926	-0.34622
H	1.88135	6.45923	1.24540
H	0.64333	8.52634	0.59979
H	-1.46808	8.42212	-0.59946
H	-2.49887	6.24293	-1.24093
C	-0.55323	-1.25091	1.90197
H	-1.11448	-2.17927	1.79342
H	-0.10223	-1.23543	2.89328
H	-1.24233	-0.41254	1.82832
C	0.68388	-1.18969	-1.89555
H	1.34226	-2.05125	-1.78167
H	0.23339	-1.22890	-2.88643
H	1.27861	-0.28149	-1.82824
C	-4.23642	-2.35003	-0.46464
C	-4.24425	-3.20056	0.81052
C	-5.40721	-1.37716	-0.38010
C	-5.63350	-3.64799	1.18020
H	-3.60875	-4.08157	0.70601
C	-6.70973	-2.15876	-0.28852
H	-5.29148	-0.75240	0.50992
N	-6.72904	-3.22581	0.71217
H	-5.69041	-4.43036	1.93744
H	-7.53887	-1.48887	-0.06085
C	4.45151	-1.93967	0.46020
C	4.56797	-2.80037	-0.80334
C	5.52381	-0.86069	0.38009
C	5.94465	-3.44764	-0.85190
H	3.79685	-3.57074	-0.82531
C	6.84972	-1.42670	-0.05622
H	5.24028	-0.09189	-0.34559
N	7.07025	-2.55175	-0.58822
H	6.00510	-4.25922	-0.12128
H	7.71360	-0.78275	0.10919
H	-4.41585	-3.02120	-1.31198
H	-5.43240	-0.71597	-1.24674
H	-6.94489	-2.61795	-1.25274

H	-3.83242	-2.62386	1.64566
H	5.65282	-0.34995	1.33552
H	4.68238	-2.58542	1.31548
H	4.41832	-2.15881	-1.67641
H	6.11702	-3.90375	-1.82692

[BPPip-H]$^{+}$-o

E = –2143.64495513 Ha (all vibrational frequencies real)

C	0.64418	1.14642	0.36100
C	-0.64615	1.07330	-0.17143
C	1.28644	2.40585	0.54565
C	-1.36522	2.26058	-0.49786
C	0.58316	3.51995	0.21175
C	-0.73411	3.44779	-0.29953
H	-2.36385	2.18055	-0.90573
H	2.28616	2.43677	0.95780
C	-1.35043	-0.20407	-0.42619
C	-0.86396	-1.26929	-1.12774
C	-2.68701	-0.42560	0.04897
C	1.42512	-0.04765	0.75692
C	2.76988	-0.24373	0.30300
C	3.33888	-1.39248	0.74978
H	3.26013	0.45008	-0.36741
C	1.00095	-1.05421	1.57891
S	2.23437	-2.24873	1.76218
C	-3.19101	-1.64656	-0.25958
S	-2.02786	-2.54875	-1.17263
H	-3.22783	0.30957	0.62903
C	0.45009	-1.43975	-1.81771
H	0.90380	-0.46665	-2.00061
H	1.14579	-2.02132	-1.20801
H	0.32681	-1.94969	-2.77245
C	-0.29931	-1.21385	2.29628
H	-0.80292	-0.25121	2.37085
H	-0.96426	-1.89520	1.76125
H	-0.14589	-1.60470	3.30136
C	0.53495	5.02155	0.12410
C	-0.78155	4.94925	-0.38906
C	1.17975	6.20937	0.32164
C	-1.49987	6.06202	-0.72278
H	-2.50604	6.02640	-1.11593
C	0.44490	7.36682	-0.02094
C	-0.84118	7.29606	-0.52224

H	2.18458	6.28450	0.71267
H	0.90249	8.33751	0.11353
H	-1.35889	8.21306	-0.76885
C	-4.53321	-2.23020	0.06528
C	-5.35716	-2.49541	-1.19754
C	-5.35996	-1.33784	0.98368
C	-6.80003	-2.78661	-0.88165
H	-5.31702	-1.62406	-1.85938
C	-6.65888	-2.04363	1.34539
H	-4.80910	-1.09271	1.89217
N	-7.39742	-2.61389	0.21866
H	-7.38873	-3.19679	-1.70255
H	-6.46375	-2.85953	2.04697
C	4.67189	-1.98611	0.40320
C	4.64560	-2.68809	-0.95988
C	5.77293	-0.92666	0.39080
C	5.97093	-3.37090	-1.23259
H	3.85617	-3.43665	-0.98691
C	7.01735	-1.38860	-0.25831
H	5.46720	-0.02945	-0.15870
N	7.09157	-2.45546	-0.95218
H	6.11542	-4.24936	-0.60434
H	7.92493	-0.80575	-0.15981
H	6.02608	-0.58184	1.39426
H	4.43461	-1.95359	-1.73981
H	6.06841	-3.67342	-2.27162
H	4.93092	-2.72987	1.16041
H	-4.38226	-3.18793	0.57390
H	-5.58114	-0.39919	0.46792
H	-7.33321	-1.35756	1.85790
H	-4.95051	-3.33071	-1.77061
H	7.99072	-2.70257	-1.35348

$[BPPip\text{-}H]^{+}$-c

E = –2143.61430017 Ha (all vibrational frequencies real)

C	-1.41599	0.02861	-0.24104
C	-0.61756	-1.24903	-0.46387
C	-2.83263	-0.22057	-0.33414
C	1.35890	0.03962	0.27417
C	2.77906	-0.19644	0.36241
C	3.13615	-1.49094	0.32985
H	3.50573	0.59984	0.42455
C	0.57091	-1.24445	0.49492

S	1.77827	-2.60801	0.18197
C	-3.18204	-1.51897	-0.30944
S	-1.81220	-2.62161	-0.15891
H	-3.56920	0.56663	-0.39354
C	-0.21779	-1.29956	-1.94874
H	0.45853	-0.47730	-2.18353
H	0.26909	-2.24043	-2.19362
H	-1.11549	-1.19867	-2.55673
C	0.17311	-1.30802	1.97913
H	-0.51074	-0.49308	2.21762
H	-0.30596	-2.25410	2.21904
H	1.06961	-1.20168	2.58807
C	0.70888	1.21595	0.08086
C	-0.77419	1.21066	-0.05006
C	1.43116	2.48682	0.03228
C	-1.50613	2.47591	-0.01051
C	0.69415	3.59775	0.00325
C	-0.77860	3.59328	0.00960
H	-2.58649	2.47248	-0.02944
H	2.51151	2.49368	0.05003
C	0.65973	5.08032	-0.00895
C	-0.75265	5.07609	0.00535
C	1.39190	6.24816	-0.02602
C	-1.49094	6.24031	0.00639
H	-2.57156	6.25663	0.01799
C	0.64492	7.43018	-0.02596
C	-0.75018	7.42615	-0.00991
H	2.47244	6.26994	-0.03850
H	1.16088	8.38064	-0.03860
H	-1.27153	8.37378	-0.01077
C	-4.55694	-2.10507	-0.36289
C	-4.85645	-2.92924	0.89367
C	-5.66087	-1.06735	-0.52345
C	-6.31707	-3.27731	1.00465
H	-4.27781	-3.85463	0.91197
C	-7.00189	-1.77244	-0.66752
H	-5.67732	-0.41990	0.35754
N	-7.27641	-2.80386	0.33231
H	-6.56327	-4.03070	1.75306
H	-7.81634	-1.05002	-0.61754
C	4.51995	-2.06180	0.36953
C	4.81712	-2.83167	-0.92463
C	5.60054	-1.00496	0.57874
C	6.25180	-3.12634	-1.11611

H	4.27835	-3.77953	-0.98099
C	6.95956	-1.66254	0.70460
H	5.60827	-0.30883	-0.26209
N	7.17274	-2.62204	-0.39367
H	6.56123	-3.79837	-1.90746
H	7.76727	-0.93839	0.64575
H	-4.59437	-2.78312	-1.22298
H	-5.48341	-0.43677	-1.39481
H	-7.07610	-2.24827	-1.64930
H	-4.56525	-2.36958	1.78861
H	4.56749	-2.77407	1.19930
H	4.50011	-2.26124	-1.80579
H	5.41146	-0.43363	1.48521
H	7.06224	-2.21712	1.63675
H	8.13374	-2.88772	-0.58593

$[BPPip-H]^{+}$-TS

E = –2143.56316164 Ha (one imaginary vibrational frequency)

C	-0.73862	1.26487	-0.35312
C	0.48355	1.36657	0.35376
C	-1.46791	2.42538	-0.71226
C	1.01167	2.63061	0.71431
C	-0.93508	3.62815	-0.34935
C	0.28910	3.72976	0.35019
H	1.93612	2.68846	1.27308
H	-2.39073	2.32983	-1.26835
C	1.22932	0.14651	0.67483
C	0.54141	-1.11465	0.84989
C	2.61841	0.01519	0.54856
C	-1.27557	-0.05999	-0.67412
C	-2.62672	-0.41386	-0.55551
C	-2.88641	-1.75721	-0.66240
H	-3.40106	0.30734	-0.33062
C	-0.39270	-1.19476	-0.84494
S	-1.42030	-2.64813	-0.96383
C	3.09017	-1.26950	0.65590
S	1.79013	-2.38322	0.97586
H	3.25563	0.86029	0.32493
C	0.16557	5.22783	0.35172
C	0.70358	6.42910	0.71461
C	-0.04411	7.57088	0.34270
C	-1.23937	7.47170	-0.34241
C	-1.78885	6.22222	-0.71376

C	-1.06010	5.12619	-0.35080
H	1.63704	6.52684	1.25072
H	0.33159	8.55115	0.60282
H	-1.77136	8.37661	-0.60296
H	-2.72589	6.16437	-1.24935
C	-0.53940	-1.25572	1.90168
H	-1.07371	-2.19972	1.79326
H	-0.08739	-1.22744	2.89209
H	-1.25200	-0.43729	1.82907
C	0.69680	-1.16023	-1.89692
H	1.37739	-2.00511	-1.78885
H	0.24548	-1.20735	-2.88698
H	1.26675	-0.23646	-1.82706
C	-4.18072	-2.47872	-0.46491
C	-4.15945	-3.33338	0.80741
C	-5.38209	-1.54434	-0.37623
C	-5.53326	-3.82775	1.17543
H	-3.49533	-4.19260	0.69942
C	-6.65858	-2.36802	-0.28629
H	-5.28570	-0.91912	0.51573
N	-6.64209	-3.43961	0.70944
H	-5.56395	-4.61504	1.92900
H	-7.50828	-1.72602	-0.05431
C	4.47916	-1.79284	0.47256
C	4.62382	-2.64415	-0.79641
C	5.50735	-0.66818	0.41601
C	6.00168	-3.27321	-0.85160
H	3.88209	-3.44015	-0.81525
C	6.84173	-1.13714	-0.01168
H	5.22165	0.10045	-0.31211
N	7.04289	-2.27340	-0.55364
H	6.11336	-4.07239	-0.11934
H	7.70667	-0.49922	0.12272
H	-4.33825	-3.15247	-1.31448
H	-5.42963	-0.88170	-1.24080
H	-6.88050	-2.83010	-1.25223
H	-3.76683	-2.74628	1.64452
H	5.61807	-0.15113	1.36975
H	4.72729	-2.43014	1.32798
H	4.45920	-2.01416	-1.67303
H	6.22884	-3.67611	-1.83492
H	7.99418	-2.52045	-0.80834

[BPPip-H_2]$^{2+}$-o

E = –2144.09226100 Ha (all vibrational frequencies real)

C	0.67794	1.14330	0.36505
C	-0.61212	1.08065	-0.16838
C	1.32896	2.39798	0.55124
C	-1.32259	2.27261	-0.49610
C	0.63379	3.51704	0.21732
C	-0.68322	3.45508	-0.29620
H	-2.32070	2.20043	-0.90677
H	2.32858	2.42151	0.96398
C	-1.32477	-0.19202	-0.42455
C	-0.84655	-1.25922	-1.12969
C	-2.66163	-0.40544	0.04999
C	1.45015	-0.05703	0.75880
C	2.79297	-0.26188	0.30318
C	3.35422	-1.41514	0.74802
H	3.28743	0.42945	-0.36661
C	1.01967	-1.06147	1.58009
S	2.24483	-2.26483	1.76073
C	-3.17051	-1.62260	-0.26510
S	-2.01553	-2.53261	-1.17660
H	-3.19518	0.33334	0.63232
C	0.46578	-1.43726	-1.82041
H	0.92422	-0.46635	-2.00234
H	1.15733	-2.02305	-1.21035
H	0.33882	-1.94608	-2.77510
C	-0.28014	-1.21067	2.30075
H	-0.77810	-0.24485	2.37196
H	-0.94958	-1.89228	1.77159
H	-0.12671	-1.59695	3.30757
C	0.59747	5.01887	0.12879
C	-0.71845	4.95675	-0.38673
C	1.25196	6.20144	0.32595
C	-1.42686	6.07496	-0.72366
H	-2.43235	6.04730	-1.11915
C	0.52740	7.36432	-0.01952
C	-0.75833	7.30357	-0.52332
H	2.25674	6.26861	0.71851
H	0.99277	8.33136	0.11441
H	-1.26777	8.22454	-0.77225
C	-4.51951	-2.19405	0.05566
C	-5.34918	-2.39113	-1.21898
C	-5.30821	-1.31513	1.02277
C	-6.78386	-2.62464	-0.95506

H	-5.29428	-1.50703	-1.86482
C	-6.61519	-1.98502	1.39572
H	-4.74045	-1.13810	1.93402
N	-7.31882	-2.45660	0.18951
H	-7.43501	-2.94995	-1.75717
H	-6.46176	-2.85389	2.03493
C	4.68394	-2.01609	0.40092
C	4.65430	-2.71606	-0.96313
C	5.79063	-0.96239	0.39055
C	5.97636	-3.40490	-1.23680
H	3.86132	-3.46080	-0.99133
C	7.03247	-1.42907	-0.26002
H	5.48963	-0.06242	-0.15699
N	7.10128	-2.49494	-0.95593
H	6.11697	-4.28463	-0.60947
H	7.94282	-0.85065	-0.16091
H	6.04575	-0.62108	1.39470
H	4.44713	-1.97944	-1.74207
H	6.07198	-3.70679	-2.27617
H	4.93852	-2.76223	1.15722
H	-4.38487	-3.17476	0.52029
H	-5.51143	-0.34664	0.56148
H	-7.29112	-1.30235	1.90318
H	-4.98704	-3.22118	-1.82798
H	7.99901	-2.74545	-1.35837
H	-8.31005	-2.65206	0.28748

$[BPPip\text{-}H_2]^{2+}$-c

E = –2144.06059009 Ha (all vibrational frequencies real)

C	-1.38457	0.03036	-0.26610
C	-0.59221	-1.25207	-0.48196
C	-2.80314	-0.20984	-0.36382
C	1.38457	0.03036	0.26610
C	2.80314	-0.20984	0.36382
C	3.15655	-1.50539	0.33076
H	3.53155	0.58423	0.43134
C	0.59221	-1.25207	0.48196
S	1.79718	-2.61847	0.17422
C	-3.15655	-1.50539	-0.33076
S	-1.79718	-2.61847	-0.17422
H	-3.53155	0.58423	-0.43134
C	-0.18860	-1.31386	-1.96480
H	0.49545	-0.49817	-2.20010

H	0.29143	-2.25964	-2.20386
H	-1.08286	-1.20762	-2.57691
C	0.18860	-1.31386	1.96480
H	-0.49545	-0.49817	2.20010
H	-0.29143	-2.25964	2.20386
H	1.08286	-1.20762	2.57691
C	0.74111	1.20989	0.07059
C	-0.74111	1.20989	-0.07059
C	1.46882	2.47784	0.03236
C	-1.46882	2.47784	-0.03236
C	0.73639	3.59157	0.00264
C	-0.73639	3.59157	-0.00264
H	-2.54890	2.48025	-0.05894
H	2.54890	2.48025	0.05894
C	0.70610	5.07405	-0.00145
C	-0.70610	5.07405	0.00145
C	1.44175	6.23999	-0.00445
C	-1.44175	6.23999	0.00445
H	-2.52236	6.25897	0.00764
C	0.69772	7.42358	-0.00231
C	-0.69772	7.42358	0.00231
H	2.52236	6.25897	-0.00764
H	1.21619	8.37273	-0.00388
H	-1.21619	8.37273	0.00388
C	-4.53934	-2.07863	-0.37555
C	-4.84489	-2.83658	0.92387
C	-5.61926	-1.02470	-0.60274
C	-6.28123	-3.12628	1.11127
H	-4.30889	-3.78534	0.99132
C	-6.97664	-1.68501	-0.73147
H	-5.63391	-0.32058	0.23132
N	-7.19764	-2.62967	0.37794
H	-6.59556	-3.78766	1.90960
H	-7.78526	-0.96081	-0.68921
C	4.53934	-2.07863	0.37555
C	4.84489	-2.83658	-0.92387
C	5.61926	-1.02470	0.60274
C	6.28123	-3.12628	-1.11127
H	4.30889	-3.78534	-0.99132
C	6.97664	-1.68501	0.73147
H	5.63391	-0.32058	-0.23132
N	7.19764	-2.62967	-0.37794
H	6.59556	-3.78766	-1.90960
H	7.78526	-0.96081	0.68921

H	-4.57965	-2.79831	-1.19915
H	-5.42413	-0.46205	-1.51336
H	-7.07108	-2.25208	-1.65696
H	-4.53112	-2.25928	1.80167
H	4.57965	-2.79831	1.19915
H	4.53112	-2.25928	-1.80167
H	5.42413	-0.46205	1.51336
H	7.07108	-2.25208	1.65696
H	8.16014	-2.89094	-0.56906
H	-8.16014	-2.89094	0.56906

[BPPip-H$_2$]$^{2+}$-TS

E = –2144.01001279 Ha (one imaginary vibrational frequency)

C	-0.67487	1.28534	-0.35250
C	0.54635	1.35426	0.35809
C	-1.37167	2.46432	-0.71583
C	1.10705	2.60360	0.72098
C	-0.80867	3.65223	-0.35026
C	0.41487	3.72111	0.35472
H	2.03107	2.63687	1.28229
H	-2.29376	2.39362	-1.27679
C	1.25979	0.11482	0.67930
C	0.54065	-1.12880	0.85603
C	2.64463	-0.05133	0.55255
C	-1.24665	-0.02575	-0.67140
C	-2.60621	-0.34054	-0.55763
C	-2.89903	-1.67739	-0.66397
H	-3.35918	0.40518	-0.33994
C	-0.39436	-1.18514	-0.83744
S	-1.46095	-2.61112	-0.95771
C	3.08348	-1.34787	0.65967
S	1.75673	-2.42799	0.98164
H	3.30294	0.77707	0.32812
C	0.33127	5.22195	0.35565
C	0.89992	6.40831	0.72102
C	0.18458	7.56934	0.34616
C	-1.01015	7.50211	-0.34409
C	-1.59120	6.26812	-0.71808
C	-0.89338	5.15306	-0.35202
H	1.83349	6.48104	1.26084
H	0.58518	8.53927	0.60782
H	-1.51656	8.42096	-0.60673
H	-2.52702	6.23541	-1.25788

C	-0.54223	-1.24295	1.90887
H	-1.08622	-2.18271	1.81211
H	-0.09000	-1.20889	2.89893
H	-1.24477	-0.41670	1.82683
C	0.69473	-1.18347	-1.89077
H	1.35075	-2.04730	-1.78209
H	0.24092	-1.21868	-2.88006
H	1.29045	-0.27612	-1.82237
C	-4.21363	-2.36184	-0.46378
C	-4.24772	-3.09957	0.88173
C	-5.39705	-1.39863	-0.51587
C	-5.60502	-3.54247	1.26353
H	-3.59453	-3.97345	0.89471
C	-6.69996	-2.16784	-0.43486
H	-5.33866	-0.69452	0.31617
N	-6.66055	-3.14006	0.67264
H	-5.73780	-4.23835	2.08320
H	-7.54617	-1.51371	-0.24372
C	4.45877	-1.90531	0.47246
C	4.57717	-2.75881	-0.79785
C	5.51388	-0.80583	0.41252
C	5.93916	-3.42083	-0.85994
H	3.81612	-3.53634	-0.81453
C	6.83411	-1.30557	-0.02442
H	5.24309	-0.02921	-0.31275
N	7.00509	-2.44543	-0.56900
H	6.03613	-4.22181	-0.12759
H	7.71446	-0.68798	0.10468
H	-4.34588	-3.10869	-1.25267
H	-5.39053	-0.82518	-1.44050
H	-6.90074	-2.73127	-1.34546
H	-3.89409	-2.45138	1.69286
H	5.64273	-0.29342	1.36647
H	4.69314	-2.54897	1.32694
H	4.42467	-2.12399	-1.67311
H	6.15057	-3.82970	-1.84424
H	-7.55419	-3.50530	0.98674
H	7.94842	-2.71350	-0.83201

BPNH$_2$-o

E = –1754.95937750 Ha (all vibrational frequencies real)

C	0.26647	0.69259	-0.09728
C	0.26646	-0.69252	0.09724

C	1.49410	1.41494	-0.17484
C	1.49407	-1.41489	0.17477
C	2.64762	0.70237	-0.08387
C	2.64760	-0.70234	0.08380
H	1.46913	-2.48609	0.32266
H	1.46918	2.48614	-0.32276
C	-0.96747	-1.49997	0.23452
C	-2.00066	-1.24143	1.08203
C	-1.14944	-2.70879	-0.52426
C	-0.96744	1.50004	-0.23457
C	-1.14938	2.70895	0.52406
C	-2.32829	3.33226	0.27077
H	-0.43319	3.07251	1.24781
C	-2.00071	1.24140	-1.08196
S	-3.23943	2.45287	-0.91735
C	-2.32830	-3.33218	-0.27087
S	-3.23930	-2.45303	0.91749
H	-0.43333	-3.07219	-1.24817
C	-2.18223	-0.13993	2.07336
H	-1.21823	0.30768	2.31140
H	-2.82367	0.65231	1.67983
H	-2.62675	-0.51220	2.99624
C	-2.18218	0.13989	-2.07330
H	-1.21818	-0.30782	-2.31112
H	-2.82377	-0.65229	-1.67986
H	-2.62650	0.51216	-2.99627
C	4.15258	0.70244	-0.08513
C	4.15256	-0.70245	0.08510
C	5.30567	1.42921	-0.17334
C	5.30563	-1.42924	0.17338
H	5.32543	-2.50206	0.30370
C	6.50444	0.68591	-0.08329
C	6.50442	-0.68597	0.08339
H	5.32550	2.50203	-0.30365
H	7.45043	1.20636	-0.14695
H	7.45039	-1.20644	0.14710
N	-2.88388	-4.44942	-0.88991
H	-2.19691	-4.97908	-1.40864
H	-3.38593	-5.05954	-0.25998
N	-2.88398	4.44949	0.88980
H	-2.19719	4.97842	1.40953
H	-3.38475	5.06031	0.25950

$BPNH_2$-c

E = –1754.94424632 Ha (all vibrational frequencies real)

C	0.81979	1.41989	0.09387
C	2.09176	0.64179	0.41603
C	1.07065	2.82993	0.03670
C	0.81980	-1.41990	-0.09370
C	1.07067	-2.82988	-0.03638
C	2.38354	-3.16187	0.01564
H	0.29912	-3.58515	-0.00730
C	2.09172	-0.64181	-0.41602
S	3.48101	-1.78063	0.00521
C	2.38348	3.16186	-0.01563
S	3.48096	1.78067	-0.00545
H	0.29910	3.58522	0.00801
C	2.12831	0.39245	1.93218
H	1.29921	-0.25255	2.22310
H	3.06285	-0.07476	2.23408
H	2.02874	1.34715	2.44625
C	2.12805	-0.39252	-1.93219
H	1.29893	0.25250	-2.22302
H	3.06256	0.07463	-2.23428
H	2.02835	-1.34723	-2.44621
C	-0.35602	-0.74342	0.02303
C	-0.35601	0.74341	-0.02281
C	-1.62415	-1.45691	0.15474
C	-1.62415	1.45691	-0.15458
C	-2.74263	-0.73062	0.09312
C	-2.74262	0.73058	-0.09304
H	-1.62271	2.53244	-0.26752
H	-1.62270	-2.53246	0.26765
C	-4.22583	-0.70098	0.09423
C	-4.22582	0.70097	-0.09423
C	-5.39294	-1.42722	0.19350
C	-5.39292	1.42722	-0.19358
H	-5.41295	2.49833	-0.33870
C	-6.57903	-0.69067	0.09374
C	-6.57902	0.69068	-0.09389
H	-5.41299	-2.49833	0.33861
H	-7.52761	-1.20583	0.16370
H	-7.52760	1.20584	-0.16390
N	2.93446	-4.40332	0.15402
H	2.32348	-5.18216	-0.03903
H	3.87355	-4.53065	-0.18874
N	2.93456	4.40336	-0.15436

H	2.32327	5.18194	0.03895
H	3.87303	4.53066	0.19026!

BPNH$_2$-TS

E = –1754.88352787 Ha (one imaginary vibrational frequency)

C	0.38608	0.68902	-0.17332
C	0.38608	-0.68902	0.17333
C	1.60603	1.39224	-0.33753
C	1.60603	-1.39224	0.33753
C	2.76234	0.68809	-0.16247
C	2.76234	-0.68809	0.16246
H	1.58720	-2.43736	0.61678
H	1.58720	2.43736	-0.61678
C	-0.88283	-1.40090	0.32568
C	-2.08056	-0.69843	0.71066
C	-1.11300	-2.70807	-0.14215
C	-0.88283	1.40090	-0.32567
C	-1.11299	2.70807	0.14216
C	-2.42828	3.09549	0.11292
H	-0.34113	3.33555	0.56785
C	-2.08056	0.69843	-0.71066
S	-3.44859	1.83772	-0.54339
C	-2.42828	-3.09548	-0.11292
S	-3.44860	-1.83771	0.54338
H	-0.34114	-3.33557	-0.56783
C	4.26483	-0.68983	0.16348
C	5.41813	-1.40096	0.33174
C	6.61899	-0.67173	0.15916
C	6.61899	0.67173	-0.15916
C	5.41813	1.40095	-0.33174
C	4.26483	0.68983	-0.16348
H	5.43877	-2.45269	0.58071
H	7.56468	-1.18259	0.28028
H	7.56468	1.18259	-0.28028
H	5.43877	2.45269	-0.58072
C	-2.13771	0.09942	1.99389
H	-3.02475	0.73327	2.02890
H	-2.16377	-0.57647	2.84841
H	-1.25596	0.72940	2.08690
C	-2.13771	-0.09942	-1.99389
H	-3.02476	-0.73326	-2.02890
H	-2.16376	0.57647	-2.84841
H	-1.25596	-0.72940	-2.08690

N	-2.99560	4.22950	0.65452
H	-2.33232	4.96482	0.85220
H	-3.81008	4.58313	0.17413
N	-2.99562	-4.22951	-0.65452
H	-2.33231	-4.96478	-0.85227
H	-3.81001	-4.58320	-0.17403

[BPNH$_2$-H]$^+$-o

E = –1755.38618080 Ha (all vibrational frequencies real)

C	0.29663	0.71370	-0.10324
C	0.28344	-0.66760	0.10437
C	1.53233	1.42011	-0.18800
C	1.49822	-1.40870	0.19136
C	2.67569	0.69227	-0.09108
C	2.65983	-0.71101	0.09110
H	1.46138	-2.47755	0.35428
H	1.52036	2.49003	-0.34507
C	-0.96391	-1.45348	0.24795
C	-1.99588	-1.18773	1.10551
C	-1.18165	-2.64545	-0.51307
C	-0.93162	1.52768	-0.24300
C	-1.11121	2.73314	0.52016
C	-2.29002	3.35819	0.26774
H	-0.39509	3.09220	1.24600
C	-1.96372	1.27305	-1.09369
S	-3.20103	2.48466	-0.92535
C	-2.36734	-3.21848	-0.22241
S	-3.25635	-2.36494	0.97222
H	-0.48814	-3.02610	-1.24997
C	-2.15064	-0.07786	2.09093
H	-1.17322	0.33733	2.33013
H	-2.76238	0.72685	1.67891
H	-2.61616	-0.43167	3.00930
C	-2.13644	0.18465	-2.10176
H	-1.17418	-0.27770	-2.31782
H	-2.81142	-0.59610	-1.74235
H	-2.54351	0.57567	-3.03395
C	4.18038	0.67416	-0.09286
C	4.16420	-0.72810	0.09158
C	5.34128	1.38753	-0.18887
C	5.30864	-1.46789	0.18668
H	5.31622	-2.53940	0.32795
C	6.53069	0.63140	-0.09205

C	6.51494	-0.73913	0.08839
H	5.37294	2.45864	-0.32971
H	7.48265	1.13996	-0.16125
H	7.45503	-1.26947	0.15667
N	-2.88857	-4.43026	-0.84127
H	-3.05611	-5.17628	-0.16029
H	-3.76652	-4.26666	-1.34204
N	-2.84478	4.47147	0.88982
H	-2.16037	5.00243	1.41031
H	-3.35785	5.07988	0.26743
H	-2.21510	-4.78865	-1.52279

$[BPNH_2\text{-}H]^+$-c

E = –1755.36572499 Ha (all vibrational frequencies real)

C	0.79706	-1.45528	-0.08256
C	2.07457	-0.69409	-0.42624
C	1.02468	-2.84733	0.01139
C	0.82253	1.36951	0.09797
C	1.07194	2.78241	0.05026
C	2.37522	3.07322	-0.00551
H	0.30196	3.54083	0.05294
C	2.09616	0.58876	0.40414
S	3.49500	1.72396	-0.05927
C	2.34864	-3.20026	0.04691
S	3.45917	-1.84305	-0.03306
H	0.24815	-3.59157	0.09959
C	2.08325	-0.45215	-1.94526
H	1.25800	0.20195	-2.22453
H	3.01810	0.00120	-2.26598
H	1.96283	-1.40785	-2.45224
C	2.17482	0.34899	1.91865
H	1.34156	-0.27808	2.23691
H	3.10916	-0.13177	2.19886
H	2.10393	1.30705	2.43124
C	-0.36625	0.71178	-0.01729
C	-0.38694	-0.76347	0.01619
C	-1.62672	1.44226	-0.13220
C	-1.65394	-1.47159	0.12238
C	-2.74862	0.72014	-0.07408
C	-2.77007	-0.73823	0.07791
H	-1.65906	-2.54927	0.20567
H	-1.62183	2.51835	-0.22947
C	-4.23468	0.71594	-0.07544

C	-4.25357	-0.68883	0.07768
C	-5.38653	1.46500	-0.15788
C	-5.43091	-1.39961	0.15753
H	-5.46870	-2.47329	0.27541
C	-6.58472	0.74483	-0.07731
C	-6.60434	-0.64008	0.07556
H	-5.38754	2.53942	-0.27630
H	-7.52523	1.27557	-0.13476
H	-7.56085	-1.14204	0.13219
N	2.91903	4.42487	-0.03567
H	3.53285	4.60457	0.76524
H	3.46953	4.59650	-0.88320
N	2.85597	-4.43576	0.13793
H	2.24296	-5.22681	0.24019
H	3.84421	-4.59449	0.22400
H	2.16877	5.11981	-0.01107

[BPNH$_2$-H]$^+$-TS

E = N/A
Transition structure could not be located.

[BPNH$_2$-H$_2$]$^{2+}$-o

E = –1755.80932198 Ha (all vibrational frequencies real)

C	-0.30732	-0.68919	-0.10757
C	-0.30732	0.68916	0.10756
C	-1.53074	-1.41430	-0.19958
C	-1.53074	1.41427	0.19961
C	-2.68258	-0.70119	-0.09598
C	-2.68258	0.70115	0.09609
H	-1.50705	2.48239	0.36813
H	-1.50706	-2.48243	-0.36811
C	0.93271	1.48535	0.25139
C	1.96180	1.23410	1.11686
C	1.14307	2.67409	-0.51659
C	0.93271	-1.48538	-0.25143
C	1.14311	-2.67410	0.51656
C	2.32272	-3.25793	0.22372
H	0.44928	-3.04402	1.25849
C	1.96178	-1.23412	-1.11692
S	3.21303	-2.42051	-0.98204
C	2.32264	3.25798	-0.22370
S	3.21302	2.42052	0.98198

H	0.44921	3.04404	-1.25848
C	2.11266	0.13843	2.11982
H	1.13790	-0.29421	2.33845
H	2.76076	-0.65433	1.74149
H	2.54144	0.51422	3.04717
C	2.11263	-0.13844	-2.11987
H	1.13786	0.29417	-2.33851
H	2.76069	0.65435	-1.74152
H	2.54144	-0.51420	-3.04721
C	-4.18684	-0.70019	-0.09707
C	-4.18684	0.70015	0.09716
C	-5.33925	-1.42689	-0.19786
C	-5.33925	1.42686	0.19786
H	-5.35869	2.49712	0.34647
C	-6.53634	-0.68491	-0.09509
C	-6.53634	0.68488	0.09502
H	-5.35869	-2.49715	-0.34648
H	-7.48254	-1.20355	-0.16742
H	-7.48254	1.20354	0.16729
N	2.83467	4.47134	-0.84663
H	2.98662	5.22456	-0.16975
H	3.71892	4.31516	-1.33880
N	2.83479	-4.47123	0.84674
H	2.98643	-5.22462	0.16998
H	3.71918	-4.31506	1.33865
H	2.16270	4.81703	-1.53635
H	2.16296	-4.81669	1.53672

$[BPNH_2\text{-}H_2]^{2+}$-c

E = –1755.77540445 Ha (all vibrational frequencies real)

C	-0.79030	-1.40009	0.10179
C	-2.07800	-0.64323	0.41161
C	-1.02111	-2.82245	0.04777
C	-0.79030	1.40009	-0.10179
C	-1.02111	2.82245	-0.04778
C	-2.31982	3.12013	0.02105
H	-0.24252	3.57073	-0.04718
C	-2.07800	0.64323	-0.41161
S	-3.45881	1.79588	0.05210
C	-2.31982	-3.12013	-0.02106
S	-3.45881	-1.79588	-0.05208
H	-0.24251	-3.57073	0.04718
C	-2.15152	-0.42462	1.93093

H	-1.33855	0.22689	2.25137
H	-3.09993	0.01933	2.22257
H	-2.04468	-1.38549	2.43133
C	-2.15154	0.42463	-1.93093
H	-1.33857	-0.22689	-2.25137
H	-3.09994	-0.01932	-2.22257
H	-2.04469	1.38550	-2.43133
C	0.39136	0.74452	0.01846
C	0.39136	-0.74452	-0.01847
C	1.65634	1.46453	0.14398
C	1.65634	-1.46453	-0.14399
C	2.76788	0.73141	0.08487
C	2.76788	-0.73141	-0.08486
H	1.66048	-2.53936	-0.24819
H	1.66048	2.53936	0.24818
C	4.24863	0.70035	0.08518
C	4.24863	-0.70035	-0.08517
C	5.41383	1.43258	0.17551
C	5.41383	-1.43258	-0.17551
H	5.43209	-2.50493	-0.30682
C	6.59524	0.69325	0.08508
C	6.59524	-0.69324	-0.08509
H	5.43209	2.50493	0.30682
H	7.54488	1.20674	0.14811
H	7.54488	-1.20674	-0.14812
N	-2.84427	4.47882	0.07830
H	-3.46219	4.67697	-0.71572
H	-3.38768	4.64054	0.93280
N	-2.84428	-4.47882	-0.07831
H	-3.46217	-4.67697	0.71572
H	-3.38771	-4.64052	-0.93280
H	-2.08615	5.16615	0.06001
H	-2.08615	-5.16615	-0.06006

$[BPNH_2\text{-}H_2]^{2+}$-TS

E = –1755.72563260 Ha (one imaginary vibrational frequency)

C	0.43706	0.67902	-0.19246
C	0.43707	-0.67902	0.19239
C	1.65055	1.38188	-0.39138
C	1.65055	-1.38189	0.39128
C	2.80302	0.68084	-0.19204
C	2.80302	-0.68087	0.19186
H	1.63236	-2.41586	0.70734

H	1.63236	2.41586	-0.70742
C	-0.84204	-1.37927	0.34037
C	-2.04366	-0.65265	0.70815
C	-1.08670	-2.67679	-0.11047
C	-0.84205	1.37927	-0.34039
C	-1.08671	2.67679	0.11047
C	-2.41097	3.01129	0.05799
H	-0.32588	3.32814	0.51839
C	-2.04368	0.65266	-0.70815
S	-3.43545	1.76837	-0.56203
C	-2.41095	-3.01130	-0.05796
S	-3.43544	-1.76837	0.56207
H	-0.32588	-3.32815	-0.51838
C	4.30557	-0.68039	0.19330
C	5.45762	-1.38593	0.39438
C	6.65510	-0.66483	0.18944
C	6.65510	0.66488	-0.18928
C	5.45762	1.38595	-0.39434
C	4.30558	0.68037	-0.19341
H	5.47714	-2.42520	0.68998
H	7.60127	-1.16847	0.33287
H	7.60127	1.16854	-0.33261
H	5.47715	2.42523	-0.68991
C	-2.08499	0.13904	1.99954
H	-2.96996	0.77321	2.04717
H	-2.11002	-0.54914	2.84228
H	-1.19501	0.75622	2.09387
C	-2.08507	-0.13901	-1.99955
H	-2.97014	-0.77303	-2.04723
H	-2.10996	0.54919	-2.84228
H	-1.19519	-0.75633	-2.09385
N	-2.97674	4.24163	0.59005
H	-3.57154	4.72372	-0.09072
H	-3.54514	4.07499	1.42766
N	-2.97669	-4.24167	-0.58998
H	-3.57123	-4.72393	0.09089
H	-3.54532	-4.07504	-1.42744
H	-2.23206	-4.89276	-0.85302
H	-2.23215	4.89285	0.85284

BPPhe-$(OH)_2$-o

E = –2256.80252882 Ha (all vibrational frequencies real)

C	-0.64521	1.36279	-0.26869
C	0.64520	1.36266	0.26848
C	-1.32572	2.58892	-0.52673
C	1.32590	2.58868	0.52660
C	-0.65842	3.74208	-0.25819
C	0.65876	3.74195	0.25816
H	2.32495	2.56381	0.94059
H	-2.32479	2.56422	-0.94070
C	1.38905	0.12560	0.59863
C	0.93139	-0.91368	1.36017
C	2.72856	-0.08176	0.14119
C	-1.38921	0.12584	-0.59889
C	-2.72872	-0.08145	-0.14141
C	-3.27146	-1.27020	-0.52345
H	-3.24270	0.62480	0.49620
C	-0.93158	-0.91353	-1.36035
S	-2.13115	-2.15021	-1.48683
C	3.27128	-1.27049	0.52337
S	2.13090	-2.15040	1.48674
H	3.24259	0.62443	-0.49644
C	-0.37880	-1.08159	2.05680
H	-0.85911	-0.11251	2.18354
H	-1.05661	-1.71455	1.47955
H	-0.24431	-1.53365	3.03880
C	0.37853	-1.08139	-2.05714
H	0.85905	-0.11236	-2.18335
H	1.05623	-1.71485	-1.48031
H	0.24385	-1.53282	-3.03941
C	-0.65794	5.24698	-0.25917
C	0.65852	5.24685	0.25928
C	-1.33940	6.39991	-0.52742
C	1.34017	6.39965	0.52764
H	2.34561	6.41919	0.92382
C	-0.64253	7.59832	-0.25303
C	0.64348	7.59819	0.25335
H	-2.34484	6.41965	-0.92360
H	-1.13004	8.54441	-0.44530
H	1.13114	8.54419	0.44571
C	4.59983	-1.81044	0.21867
C	4.85870	-3.18071	0.24658
C	5.65214	-0.95230	-0.11557
C	6.11573	-3.68252	-0.04981
H	4.06363	-3.87404	0.48998
C	6.90764	-1.44184	-0.42152

H	5.48863	0.11704	-0.12156
H	6.29530	-4.75003	-0.02438
H	7.71881	-0.77405	-0.67662
C	-4.60000	-1.81011	-0.21865
C	-4.85890	-3.18038	-0.24644
C	-5.65230	-0.95193	0.11554
C	-6.11592	-3.68214	0.05006
H	-4.06385	-3.87374	-0.48983
C	-6.90779	-1.44142	0.42157
H	-5.48878	0.11741	0.12141
H	-6.29551	-4.74966	0.02473
H	-7.71895	-0.77360	0.67659
C	7.14516	-2.81282	-0.38920
C	-7.14532	-2.81240	0.38941
O	8.39825	-3.24165	-0.69421
H	8.44184	-4.20163	-0.63477
O	-8.39838	-3.24121	0.69458
H	-8.44208	-4.20116	0.63486

BPPhe-(OH)$_2$-c

E = –2256.77299330 Ha (all vibrational frequencies real)

C	1.40546	0.26988	0.14527
C	0.62839	-1.00629	0.43439
C	2.81701	0.02603	0.11773
C	-1.40545	0.26986	-0.14527
C	-2.81701	0.02599	-0.11772
C	-3.17737	-1.27692	-0.07800
H	-3.54762	0.82004	-0.08524
C	-0.62837	-1.00629	-0.43441
S	-1.79455	-2.37956	-0.04738
C	3.17740	-1.27688	0.07798
S	1.79459	-2.37953	0.04731
H	3.54760	0.82009	0.08528
C	0.33741	-1.05283	1.94454
H	-0.31554	-0.22678	2.22709
H	-0.13617	-1.99044	2.22555
H	1.27760	-0.95662	2.48537
C	-0.33740	-1.05278	-1.94457
H	0.31552	-0.22671	-2.22710
H	0.13621	-1.99037	-2.22561
H	-1.27759	-0.95659	-2.48539
C	-0.74241	1.45272	-0.00583
C	0.74239	1.45273	0.00588

C	-1.46353	2.71897	0.09435
C	1.46349	2.71899	-0.09427
C	-0.73286	3.83513	0.06124
C	0.73281	3.83514	-0.06107
H	2.54203	2.72060	-0.16036
H	-2.54207	2.72056	0.16041
C	-0.70373	5.31850	0.06298
C	0.70365	5.31851	-0.06284
C	-1.43543	6.48431	0.13038
C	1.43532	6.48433	-0.13030
H	2.51159	6.50357	-0.22797
C	-0.69448	7.66910	0.06332
C	0.69435	7.66911	-0.06331
H	-2.51169	6.50352	0.22804
H	-1.21142	8.61799	0.11057
H	1.21127	8.61801	-0.11061
C	4.53183	-1.82426	0.03330
C	4.76068	-3.16701	-0.28286
C	5.64353	-1.01840	0.30243
C	6.04048	-3.68630	-0.34125
H	3.92497	-3.82060	-0.49704
C	6.92687	-1.52615	0.24783
H	5.50671	0.01895	0.57423
H	6.21057	-4.72440	-0.58985
H	7.77512	-0.88878	0.46385
C	-4.53179	-1.82432	-0.03331
C	-4.76068	-3.16692	0.28345
C	-5.64345	-1.01860	-0.30303
C	-6.04049	-3.68619	0.34183
H	-3.92500	-3.82038	0.49815
C	-6.92680	-1.52633	-0.24844
H	-5.50657	0.01862	-0.57530
H	-6.21061	-4.72417	0.59091
H	-7.77500	-0.88906	-0.46488
C	-7.13104	-2.86558	0.07718
C	7.13106	-2.86553	-0.07723
O	-8.36688	-3.41856	0.14426
H	-9.03721	-2.75775	-0.05931
O	8.36693	-3.41851	-0.14438
H	9.03723	-2.75761	0.05899

BPPhe-$(OH)_2$-TS

E = –2256.72706154 Ha (one imaginary vibrational frequency)

C	-0.63633	1.53361	-0.31967
C	0.62534	1.53787	0.31453
C	-1.28970	2.74677	-0.64754
C	1.26972	2.75549	0.64376
C	-0.64534	3.90397	-0.31853
C	0.61674	3.90825	0.31620
H	2.22604	2.73825	1.14879
H	-2.24584	2.72297	-1.15266
C	1.29846	0.26112	0.58827
C	0.53140	-0.94131	0.82344
C	2.65072	0.02934	0.37090
C	-1.30015	0.25219	-0.59468
C	-2.65017	0.01002	-0.37575
C	-3.03505	-1.31888	-0.46381
H	-3.33481	0.79267	-0.07883
C	-0.52436	-0.94420	-0.83195
S	-1.66079	-2.31060	-0.88004
C	3.04525	-1.29666	0.45790
S	1.67787	-2.29943	0.87028
H	3.33018	0.81722	0.07593
C	0.61208	5.41207	0.31916
C	1.26079	6.56659	0.65058
C	0.58747	7.76419	0.31322
C	-0.64462	7.76001	-0.31072
C	-1.30908	6.55790	-0.64955
C	-0.65184	5.40778	-0.31960
H	2.22517	6.58981	1.13812
H	1.05201	8.71155	0.55087
H	-1.11611	8.70421	-0.54728
H	-2.27360	6.57461	-1.13708
C	-0.49646	-0.99555	1.93267
H	-1.12448	-1.88246	1.84664
H	0.01068	-1.02513	2.89611
H	-1.13010	-0.11214	1.90989
C	0.50394	-0.98939	-1.94112
H	1.13826	-1.87193	-1.85628
H	-0.00274	-1.02107	-2.90472
H	1.13132	-0.10156	-1.91679
C	-4.34072	-1.89483	-0.20612
C	-4.53640	-3.28269	-0.15819
C	-5.46186	-1.07781	-0.00063
C	-5.78114	-3.82995	0.08717
H	-3.69719	-3.94933	-0.30965
C	-6.71010	-1.61668	0.24486

H	-5.36176	-0.00219	-0.04083
H	-5.91970	-4.90156	0.12442
H	-7.56202	-0.96610	0.39892
C	4.35632	-1.86196	0.20432
C	4.56135	-3.24580	0.14494
C	5.47524	-1.03408	0.01617
C	5.81458	-3.78233	-0.09434
H	3.72620	-3.92066	0.28130
C	6.72774	-1.56034	-0.22185
H	5.36548	0.04023	0.06636
H	5.94690	-4.85616	-0.13857
H	7.58387	-0.91511	-0.36259
C	6.90559	-2.94111	-0.27856
C	-6.87705	-2.99838	0.29105
O	8.16065	-3.40511	-0.51308
H	8.15964	-4.36795	-0.52033
O	-8.08048	-3.58261	0.52770
H	-8.75616	-2.90723	0.64826

[BPPhe-O_2H]$^-$-o

E = –2256.31841450 Ha (all vibrational frequencies real)

C	-0.60178	1.35607	-0.26309
C	0.69173	1.33758	0.26800
C	-1.26860	2.59129	-0.51456
C	1.38765	2.55589	0.52372
C	-0.58662	3.73605	-0.24673
C	0.73323	3.71802	0.26208
H	2.38917	2.51698	0.93045
H	-2.26984	2.57961	-0.92402
C	1.42386	0.09231	0.59341
C	0.95348	-0.94295	1.34829
C	2.76246	-0.12406	0.12946
C	-1.36434	0.13070	-0.59545
C	-2.70672	-0.05771	-0.13808
C	-3.26836	-1.23662	-0.52352
H	-3.20933	0.65411	0.50252
C	-0.92338	-0.91343	-1.36014
S	-2.14214	-2.13096	-1.49013
C	3.30358	-1.32149	0.49845
S	2.14819	-2.19212	1.46634
H	3.27659	0.58266	-0.50729
C	-0.35884	-1.10685	2.04216
H	-0.83693	-0.13648	2.16952

H	-1.04090	-1.73753	1.46647
H	-0.22841	-1.55850	3.02540
C	0.38554	-1.10237	-2.05321
H	0.87668	-0.13986	-2.18826
H	1.05624	-1.73390	-1.46626
H	0.24783	-1.56376	-3.03045
C	-0.56735	5.24085	-0.24464
C	0.75222	5.22296	0.26634
C	-1.23529	6.40306	-0.50662
C	1.44986	6.36652	0.53273
H	2.45784	6.37244	0.92292
C	-0.52163	7.59225	-0.23395
C	0.76700	7.57472	0.26491
H	-2.24265	6.43655	-0.89707
H	-0.99826	8.54483	-0.42163
H	1.26796	8.51397	0.45627
C	4.61804	-1.87946	0.20018
C	4.89910	-3.24743	0.32914
C	5.66960	-1.05857	-0.23813
C	6.14026	-3.77108	0.04000
H	4.11141	-3.92046	0.65311
C	6.91536	-1.56415	-0.53424
H	5.50080	0.00872	-0.33039
H	6.31776	-4.83499	0.14551
H	7.70626	-0.89947	-0.86262
C	-4.60459	-1.75721	-0.21882
C	-4.88375	-3.12350	-0.24848
C	-5.64412	-0.88442	0.11771
C	-6.14789	-3.60723	0.04801
H	-4.09907	-3.82805	-0.49338
C	-6.90654	-1.35595	0.42377
H	-5.46477	0.18237	0.12542
H	-6.34304	-4.67198	0.02147
H	-7.70763	-0.67673	0.68059
C	7.23165	-2.95895	-0.41313
C	-7.16428	-2.72316	0.38915
O	8.37794	-3.43044	-0.68089
O	-8.42371	-3.13430	0.69382
H	-8.48138	-4.09330	0.63069

[BPPhe-O_2H]$^-$-c

$E = -2256.29503499$ Ha (all vibrational frequencies real)

C	-1.36762	0.28174	-0.14232

C	-0.60285	-1.00247	-0.42736
C	-2.78160	0.05332	-0.11847
C	1.45411	0.24616	0.13789
C	2.84806	-0.01748	0.09882
C	3.20665	-1.33818	0.07591
H	3.58778	0.76643	0.04662
C	0.65437	-1.01281	0.44237
S	1.79395	-2.40892	0.08195
C	-3.16091	-1.24385	-0.08081
S	-1.78485	-2.36171	-0.03247
H	-3.50283	0.85688	-0.09439
C	-0.30636	-1.06060	-1.93528
H	0.35025	-0.23796	-2.22017
H	0.16598	-2.00120	-2.20925
H	-1.24416	-0.96463	-2.48054
C	0.35670	-1.02748	1.95221
H	-0.28225	-0.18552	2.21877
H	-0.13595	-1.95156	2.24566
H	1.29702	-0.94103	2.49448
C	0.79451	1.44310	0.00918
C	-0.68605	1.45600	-0.00360
C	1.52854	2.69741	-0.07989
C	-1.39163	2.73199	0.09542
C	0.81585	3.82804	-0.05129
C	-0.64799	3.84066	0.06388
H	-2.47040	2.74688	0.16028
H	2.60808	2.68308	-0.13640
C	0.80180	5.31179	-0.04837
C	-0.60749	5.32562	0.07036
C	1.54201	6.47243	-0.10733
C	-1.32846	6.49663	0.13846
H	-2.40518	6.52446	0.23089
C	0.81049	7.66463	-0.03976
C	-0.57769	7.67747	0.07954
H	2.61901	6.48410	-0.19911
H	1.33697	8.60872	-0.08099
H	-1.08720	8.63035	0.12756
C	-4.52123	-1.77663	-0.04543
C	-4.76654	-3.12142	0.24977
C	-5.62572	-0.95588	-0.30145
C	-6.05277	-3.62774	0.30065
H	-3.93812	-3.78794	0.45210
C	-6.91483	-1.45036	-0.25333
H	-5.47833	0.08432	-0.55655

H	-6.23350	-4.66801	0.53264
H	-7.75601	-0.79999	-0.45867
C	4.52421	-1.90639	0.02576
C	4.73731	-3.29748	-0.07485
C	5.67968	-1.09187	0.06750
C	5.99288	-3.84335	-0.13294
H	3.88024	-3.96132	-0.10904
C	6.94096	-1.61745	0.01201
H	5.56755	-0.01785	0.15249
H	6.12055	-4.91605	-0.21109
H	7.80613	-0.96663	0.05008
C	7.18303	-3.03535	-0.09324
C	-7.13439	-2.79203	0.05076
O	8.34159	-3.52337	-0.14422
O	-8.37830	-3.33229	0.10994
H	-9.03963	-2.65908	-0.08139

[BPPhe-O_2H]$^-$-TS

E = –2256.24665093 Ha (one imaginary vibrational frequency)

C	0.56127	1.53151	0.33025
C	-0.69798	1.49192	-0.31209
C	1.17043	2.76836	0.65388
C	-1.37727	2.68675	-0.65194
C	0.49238	3.90373	0.31246
C	-0.76443	3.86373	-0.32874
H	-2.32987	2.63492	-1.16192
H	2.12291	2.77894	1.16663
C	-1.33303	0.19335	-0.58194
C	-0.53182	-0.98605	-0.80838
C	-2.67907	-0.06831	-0.36983
C	1.26695	0.27527	0.61615
C	2.62660	0.07870	0.39504
C	3.06113	-1.23279	0.48156
H	3.28204	0.88611	0.09667
C	0.53330	-0.94653	0.84841
S	1.71943	-2.27158	0.90235
C	-3.05267	-1.40777	-0.45073
S	-1.64657	-2.37321	-0.85779
H	-3.37730	0.70608	-0.08322
C	-0.80968	5.36698	-0.34020
C	-1.49343	6.49815	-0.68024
C	-0.86134	7.71987	-0.34546
C	0.36613	7.75914	0.28475

C	1.06766	6.57989	0.63333
C	0.45099	5.40723	0.30579
H	-2.45566	6.48768	-1.17259
H	-1.35566	8.65015	-0.59069
H	0.80553	8.71928	0.51919
H	2.02823	6.63081	1.12645
C	0.49925	-1.01869	-1.91463
H	1.13393	-1.90199	-1.83547
H	-0.00171	-1.04112	-2.88199
H	1.12817	-0.13204	-1.87820
C	-0.49950	-1.03279	1.95082
H	-1.09958	-1.93868	1.86146
H	-0.00016	-1.04367	2.91895
H	-1.16213	-0.17089	1.91995
C	4.38345	-1.76528	0.21537
C	4.62634	-3.14605	0.16924
C	5.47760	-0.91438	-0.00182
C	5.88705	-3.65327	-0.08583
H	3.81111	-3.83972	0.33017
C	6.74111	-1.41337	-0.25701
H	5.34315	0.15773	0.03636
H	6.05932	-4.72022	-0.12118
H	7.56994	-0.73519	-0.41976
C	-4.33162	-2.00090	-0.20088
C	-4.54605	-3.39408	-0.28744
C	-5.45582	-1.21306	0.14136
C	-5.77162	-3.96286	-0.05221
H	-3.71378	-4.03926	-0.54847
C	-6.68665	-1.76485	0.37712
H	-5.34422	-0.13841	0.22004
H	-5.89835	-5.03631	-0.12752
H	-7.52612	-1.13114	0.63709
C	-6.92814	-3.18202	0.29569
C	6.95349	-2.78858	-0.30159
O	-8.06088	-3.69373	0.51149
O	8.17571	-3.33470	-0.54759
H	8.82550	-2.63646	-0.67770

[BPPhe-O_2]$^{2-}$-o

E = –2255.83273661 Ha (all vibrational frequencies real)

C	-0.64848	1.32969	-0.26242
C	0.64849	1.32965	0.26233
C	-1.33056	2.55739	-0.51159

C	1.33063	2.55731	0.51154
C	-0.66128	3.71124	-0.25070
C	0.66139	3.71120	0.25068
H	2.33449	2.53159	0.91374
H	-2.33443	2.53172	-0.91379
C	1.39992	0.09610	0.58985
C	0.94726	-0.94422	1.34838
C	2.74194	-0.10044	0.12625
C	-1.39996	0.09617	-0.58997
C	-2.74197	-0.10035	-0.12632
C	-3.30329	-1.28691	-0.49965
H	-3.24427	0.61208	0.51365
C	-0.94734	-0.94416	-1.34851
S	-2.16295	-2.17273	-1.47131
C	3.30322	-1.28701	0.49960
S	2.16276	-2.17291	1.47103
H	3.24429	0.61201	-0.51367
C	-0.36417	-1.13045	2.03786
H	-0.85408	-0.16709	2.17337
H	-1.03815	-1.76029	1.45196
H	-0.23071	-1.59151	3.01634
C	0.36411	-1.13045	-2.03794
H	0.85410	-0.16712	-2.17336
H	1.03801	-1.76039	-1.45207
H	0.23064	-1.59143	-3.01646
C	-0.66131	5.21631	-0.25182
C	0.66149	5.21628	0.25185
C	-1.34536	6.36944	-0.51206
C	1.34558	6.36937	0.51213
H	2.35554	6.38910	0.89681
C	-0.64553	7.56851	-0.24576
C	0.64581	7.56848	0.24587
H	-2.35531	6.38922	-0.89674
H	-1.13553	8.51449	-0.43259
H	1.13584	8.51442	0.43273
C	4.62683	-1.82440	0.20189
C	4.92868	-3.18795	0.32986
C	5.66583	-0.98765	-0.23601
C	6.17762	-3.69262	0.03969
H	4.15125	-3.87295	0.65349
C	6.91906	-1.47440	-0.53341
H	5.48088	0.07708	-0.32724
H	6.37104	-4.75384	0.14433
H	7.70003	-0.79785	-0.86169

C	-4.62688	-1.82430	-0.20186
C	-4.92876	-3.18783	-0.32992
C	-5.66581	-0.98756	0.23621
C	-6.17768	-3.69250	-0.03966
H	-4.15138	-3.87282	-0.65368
C	-6.91902	-1.47431	0.53370
H	-5.48083	0.07716	0.32751
H	-6.37114	-4.75371	-0.14436
H	-7.69995	-0.79776	0.86212
C	7.25604	-2.86427	-0.41439
C	-7.25604	-2.86415	0.41460
O	8.40897	-3.31875	-0.68461
O	-8.40896	-3.31863	0.68489

[BPPhe-O_2]$^{2-}$-c

E = –2255.81338260 Ha (all vibrational frequencies real)

C	1.41492	0.26239	0.13783
C	0.62947	-1.00704	0.43442
C	2.81848	0.01252	0.10652
C	-1.41492	0.26239	-0.13783
C	-2.81848	0.01252	-0.10652
C	-3.19106	-1.29837	-0.07758
H	-3.54974	0.80589	-0.07021
C	-0.62947	-1.00704	-0.43442
S	-1.78704	-2.38727	-0.06029
C	3.19106	-1.29837	0.07758
S	1.78704	-2.38727	0.06029
H	3.54974	0.80589	0.07021
C	0.33019	-1.03864	1.94298
H	-0.31580	-0.20368	2.21555
H	-0.15579	-1.96894	2.22835
H	1.26903	-0.94905	2.48760
C	-0.33019	-1.03864	-1.94298
H	0.31580	-0.20368	-2.21555
H	0.15579	-1.96895	-2.22835
H	-1.26903	-0.94905	-2.48760
C	-0.74144	1.44610	-0.00423
C	0.74144	1.44610	0.00423
C	-1.45934	2.71200	0.09459
C	1.45934	2.71200	-0.09459
C	-0.73226	3.83284	0.06204
C	0.73226	3.83284	-0.06204
H	2.53861	2.71157	-0.16119

H	-2.53861	2.71157	0.16119
C	-0.70473	5.31682	0.06411
C	0.70473	5.31682	-0.06411
C	-1.43400	6.48348	0.13256
C	1.43400	6.48348	-0.13256
H	2.51036	6.50361	-0.23191
C	-0.69365	7.67055	0.06433
C	0.69365	7.67055	-0.06433
H	-2.51036	6.50361	0.23191
H	-1.21168	8.61897	0.11246
H	1.21168	8.61897	-0.11246
C	4.51983	-1.85732	0.03687
C	4.74705	-3.24166	-0.08753
C	5.66611	-1.03613	0.11154
C	6.01091	-3.77629	-0.13883
H	3.89692	-3.91276	-0.14695
C	6.93468	-1.55075	0.06371
H	5.54378	0.03518	0.21743
H	6.14705	-4.84664	-0.23589
H	7.79242	-0.89178	0.12793
C	-4.51983	-1.85732	-0.03687
C	-4.74705	-3.24166	0.08753
C	-5.66611	-1.03613	-0.11154
C	-6.01091	-3.77629	0.13883
H	-3.89692	-3.91276	0.14695
C	-6.93468	-1.55075	-0.06371
H	-5.54378	0.03518	-0.21743
H	-6.14705	-4.84664	0.23588
H	-7.79242	-0.89178	-0.12793
C	-7.19224	-2.96198	0.06655
C	7.19224	-2.96198	-0.06655
O	-8.35902	-3.43965	0.11054
O	8.35902	-3.43965	-0.11054

[BPPhe-O_2]$^{2-}$-TS

E = –2255.76197780 Ha (one imaginary vibrational frequency)

C	-0.63518	1.49564	-0.31369
C	0.63518	1.49564	0.31369
C	-1.28464	2.71431	-0.63139
C	1.28464	2.71431	0.63139
C	-0.63477	3.87139	-0.30932
C	0.63477	3.87139	0.30932
H	2.24560	2.69383	1.12808

H	-2.24559	2.69383	-1.12808
C	1.30878	0.22185	0.59543
C	0.55015	-0.97801	0.83881
C	2.66621	-0.00399	0.37190
C	-1.30878	0.22185	-0.59542
C	-2.66621	-0.00399	-0.37190
C	-3.07964	-1.32508	-0.45976
H	-3.33832	0.78894	-0.07234
C	-0.55015	-0.97801	-0.83881
S	-1.70561	-2.33043	-0.88834
C	3.07964	-1.32508	0.45976
S	1.70561	-2.33043	0.88834
H	3.33832	0.78894	0.07234
C	0.63676	5.37490	0.31163
C	1.29275	6.52784	0.63330
C	0.61960	7.72930	0.30386
C	-0.61959	7.72930	-0.30386
C	-1.29274	6.52784	-0.63330
C	-0.63675	5.37490	-0.31163
H	2.26351	6.54826	1.10846
H	1.09109	8.67489	0.53534
H	-1.09109	8.67489	-0.53534
H	-2.26351	6.54826	-1.10846
C	-0.48858	-1.04094	1.93477
H	-1.10694	-1.93471	1.84238
H	0.00208	-1.06123	2.90790
H	-1.13510	-0.16690	1.89869
C	0.48858	-1.04094	-1.93477
H	1.10694	-1.93471	-1.84238
H	-0.00208	-1.06123	-2.90790
H	1.13510	-0.16690	-1.89869
C	-4.37764	-1.88976	-0.20179
C	-4.63139	-3.27197	-0.30922
C	-5.47431	-1.08081	0.16854
C	-5.87168	-3.81266	-0.06745
H	-3.82117	-3.93560	-0.59286
C	-6.71918	-1.60451	0.41108
H	-5.33299	-0.01084	0.26567
H	-6.02622	-4.88129	-0.16043
H	-7.53643	-0.95138	0.69417
C	4.37764	-1.88976	0.20179
C	4.63139	-3.27197	0.30922
C	5.47431	-1.08081	-0.16855
C	5.87168	-3.81266	0.06745

H	3.82117	-3.93560	0.59286
C	6.71918	-1.60451	-0.41109
H	5.33298	-0.01085	-0.26568
H	6.02622	-4.88129	0.16044
H	7.53643	-0.95138	-0.69418
C	7.00168	-3.01072	-0.30765
C	-7.00168	-3.01072	0.30765
O	8.14866	-3.49721	-0.52817
O	-8.14866	-3.49721	0.52817
